# The cicadas (Hemiptera: Cicadidae) of India, Bangladesh, Bhutan, Myanmar, Nepal and Sri Lanka: an annotated provisional catalogue, regional checklist and bibliography

**DOI:** 10.3897/BDJ.4.e8051

**Published:** 2016-07-20

**Authors:** Benjamin Wills Price, Elizabeth Louise Allan, Kiran Marathe, Vivek Sarkar, Chris Simon, Krushnamegh Kunte

**Affiliations:** ‡ Natural History Museum, London, United Kingdom; § Ecology and Evolutionary Biology, University of Connecticut, Storrs, United States of America; | National Centre for Biological Sciences, Tata Institute of Fundamental Research, Bangalore, India

**Keywords:** Biodiversity Inventory, Systematic Catalogues, Biodiversity Hotspots, Indian Subcontinent, Himalaya, Western Ghats

## Abstract

**Background:**

The cicadas of the Indian subcontinent, like many other insects in the region, have remained understudied since the early part of the 20th Century, and await modern taxonomic, systematic and phylogenetic treatment. This paper presents an updated systematic catalogue of cicadas (Hemiptera: Cicadidae) from India, Bangladesh, Bhutan, Myanmar, Nepal and Sri Lanka, the first in over a century.

**New information:**

This paper treats 281 species, including: India and Bangladesh (189 species), Bhutan (19 species), Myanmar (81 species), Nepal (46 species) and Sri Lanka (22 species). For each species all recognized junior synonyms are included with information on the type material and additional specimens where relevant. The global distributional range and notes on the taxonomy of each species are included where appropriate. Two lists are provided: (1) species known to occur in India and Bangladesh (treated as a geographic unit), Bhutan, Myanmar, Nepal and Sri Lanka; and (2) species previously listed from these countries in error. A bibliography of species descriptions is provided, with the papers containing the original descriptions provided where copyright allows.

## Introduction

We share our planet with an estimated 8.7 million species (Mora et al. 2011), of which approximately 80% await scientific description. Insects, although understudied, are known to account for over half the world’s described biodiversity (Foottit and Adler 2009), and likely constitute a much greater proportion of the undescribed biodiversity. The cicadas (Hemiptera: Cicadidae) of the Indian subcontinent, like many other insects in the region, have remained understudied since the early part of the 20th Century and await, for the most part, modern taxonomic, systematic and phylogenetic treatment.

**Figure 1.**
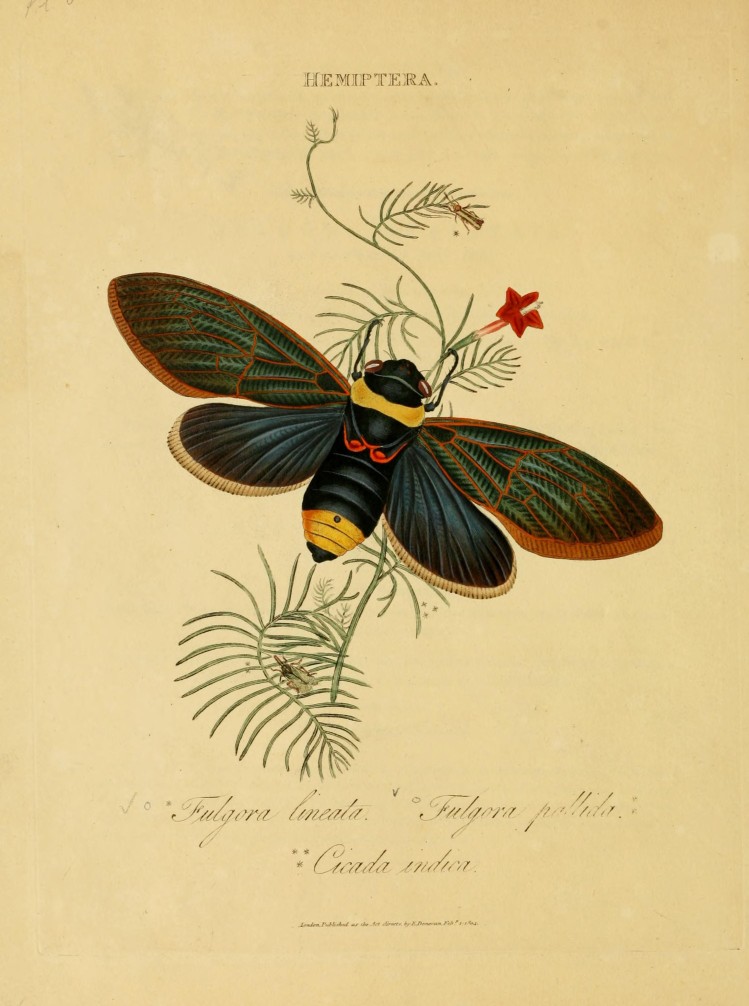
Donovan’s illustration of *Cicada indica* Donovan 1800. This stunning species is a junior synonym of *Tacua speciosa speciosa* (Illiger, 1800) and is not found on the Indian subcontinent, an example of the historical misattribution of species from the “East Indies” with India. Image source: https://www.flickr.com/photos/biodivlibrary/7138659445/​ Publication source: http://biodiversitylibrary.org/page/25494957

The first illustrated publication dealing with the insect fauna of India was “*An epitome of the natural history of the insects of India and the islands in the Indian Seas*” by Donovan (1800), which included many superb illustrations made by the author. One cicada species (*Cicada indica* Donovan 1800) was described by Donovan in his epitome, although the illustration is dated as 1st of February 1804 (Fig. 1). The original description states that the specimen was collected in Bengal, a claim doubted by Distant: "According to Donovan, a single specimen of this species was found in Bengal by Mr. Fichtel, and deposited in the Imperial Cabinet at Vienna, but that habitat I consider liable to the greatest doubt." (Distant 1889a). Donovan may have confused Fabricius’ label “Indiis” as India, when it could refer to the West Indies, East Indies or occasionally to Africa. In the present study *Cicada indica* is listed as unlikely to be from the Indian subcontinent, being synonymised with Tacua speciosa speciosa (Illiger, 1800) which is found in Indonesia and Malaysia.

By far the most influential figure in the history of cicadas in India is William Lucas Distant (12 November 1845 – 4 February 1922). Distant worked at the Natural History Museum, then the British Museum (Natural History) from 1889 to 1920, during which time he described a tremendous number of insect species. Over 100 cicada species which occur on the Indian subcontinent and were described by Distant are currently recognised as valid. His most significant works relevant to this fauna were: “*A Monograph of Oriental Cicadidae*” (Distant 1889b, Distant 1891, Distant 1892c); “*The fauna of British India, including Ceylon and Burma*” (Distant 1906c); and “*A Synonymic Catalogue of Homoptera, Part I - Cicadidae*” (Distant 1906b). In particular “*The fauna of British India, including Ceylon and Burma*” (Distant 1906c) provided the first treatment of the region as a whole, with 148 cicada species recognised in 44 genera. This was followed by an appendix (Distant 1916) which listed an additional 24 species and 3 new genera for the region, bringing the total to 172 cicada species in 47 genera.

Apart from a few isolated species descriptions, the cicada fauna of the Indian subcontinent has not been subjected to a detailed taxonomic and systematic review since Distant (1906c), Distant (1916), hence, it is highly likely that many unrecognized and undescribed cicada species exist in this region.

This study serves as a modern reference point for the cicada fauna of India, Bangladesh, Bhutan, Myanmar, Nepal and Sri Lanka, and hopefully, provides a backbone to recording efforts and studies of the underlying taxonomy. Pakistan, formerly a part of “British India” has been excluded from full review in this catalogue as it has recently been updated by Ahmed and Sanborn (2010), however the fauna are briefly compared.

The checklists presented here are full of compromises but our guiding intention has been to present all the relevant information in a manner that is as compact and informative as possible. No doubt there are errors which have crept in, or which have not been discovered in the previous literature, and we welcome all suggestions to rectify any errors or omissions. To aide future studies the next incarnation will be digital, and the list of species is already in the process of migrating to a website, where updates will be maintained as and when changes occur. The website will also allow the community to share images and information on each species, providing an up-to-date resource, hopefully for many years to come.

**Figure 2.**
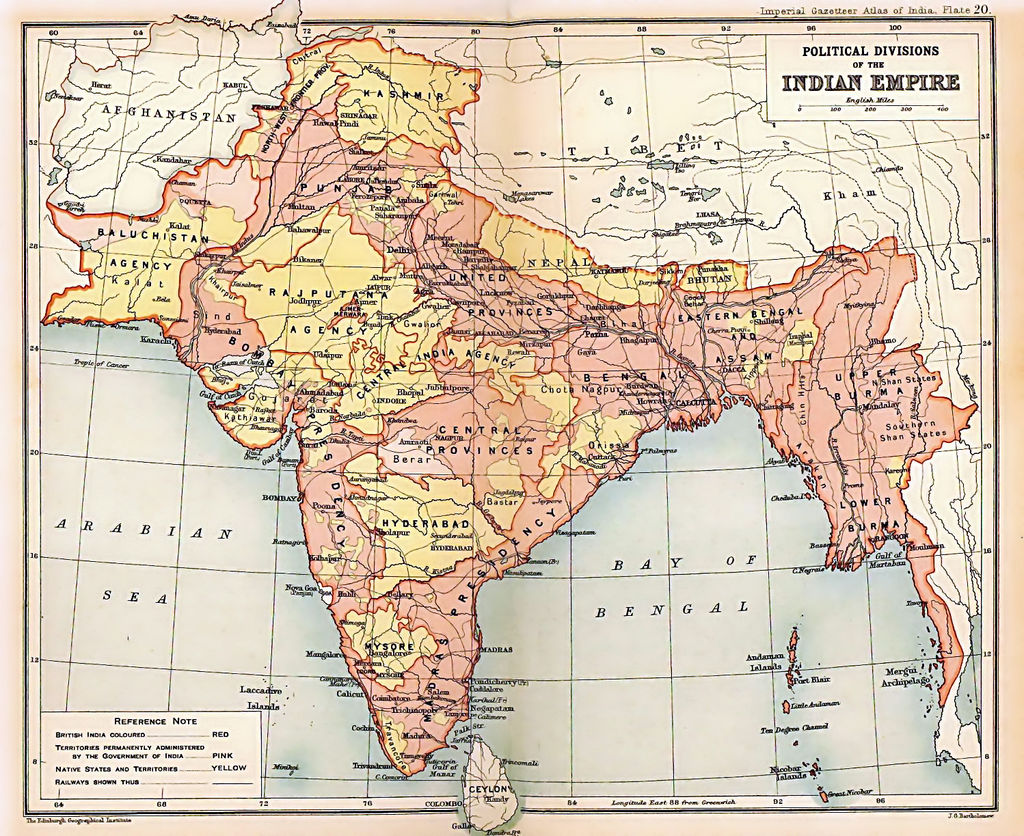
Map of the Indian subcontinent showing the historical extent of “British India” published in 1909. Map source Edinburgh Geographical Institute; J. G. Bartholomew and Sons. [Public domain], via Wikimedia Commons.

**Figure 3.**
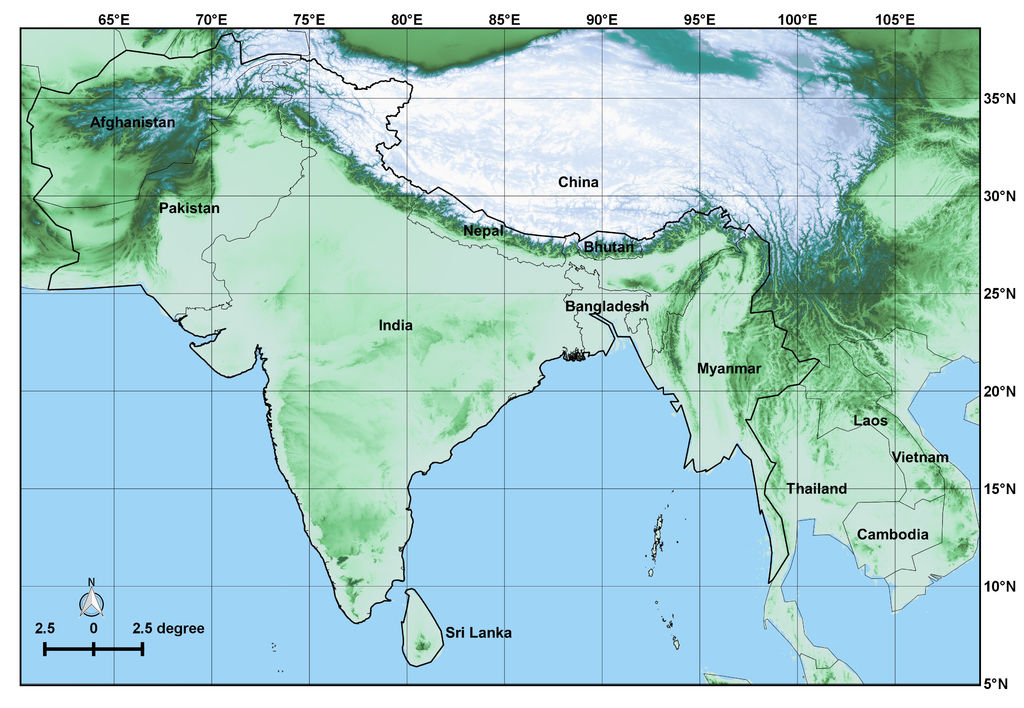
Map of the Indian subcontinent, including countries as politically delineated at present that were at one time considered part of “British India” and countries surrounding “British India” with which the fauna of Bangladesh, Bhutan, India, Myanmar, Nepal, Pakistan and to a lesser extent Sri Lanka likely overlaps.

Historically the regions making up the Indian subcontinent have had a complex nomenclature (Figs 2, 3), further compounded by different authors interpreting locality labels to mean different regions. In particular the confusion between East India, East Indies, India, Indo-China and Indonesia, will mean that some species included in this list are unlikely to occur in the focal region, but are included until further study proves otherwise.

## Materials and methods

### Checklists

The checklist was based on the “*The fauna of British India, including Ceylon and Burma*” (Distant 1906c) and its supplement (Distant 1916), the four catalogues which list the global cicada literature (Distant 1906b, Metcalf 1963, Duffels and van der Laan 1985, Sanborn 2014) and the material housed at the Natural History Museum, London. In addition species which have been described after 2010 were incorporated by searching Google Scholar (https://scholar.google.co.uk/) and ScienceDirect (http://www.sciencedirect.com/) using a combination of “India”, “Bangladesh”, “Bhutan”, “Myanmar”, “Nepal”, “Sri Lanka”, and “cicada” and “Cicadidae”. The checklist is based on either the locality of the type specimen, the locality of specimens whose identity has been verified or substantial literature evidence. This checklist follows the higher taxonomy of Sanborn (2014)​ with updated generic combinations including current information as of September 2015. Institutional abbreviations are summarized in Table 1.

Due to the significant gap since the last major reviews by Distant, and the dramatic changes in the geographic boundaries of the countries involved (Figs 2, 3), two lists have been developed:

(1) Species present in India and Bangladesh (treated here as a single unit), Bhutan, Myanmar, Nepal and Sri Lanka.

(2) Species historically included in the countries listed above, but on analysis are very unlikely to be present in these countries, with reasons for their exclusion listed in the notes.

We have not considered the cicada fauna of Pakistan as these species have recently been studied (Ahmed and Sanborn 2010).

### Specimen Images

All type specimens examined were photographed (dorsal and ventral) and are accessible at: http://www.indiancicadas.org/.

### Bibliography

For each species recorded from the Indian subcontinent the original description was sourced. In addition, any publication listing the focal region (as summarized by the four major catalogues) was sourced and confirmed. The citation of each authority is linked in this manuscript through the Notes field and in addition is publically accessible on Mendeley as the “Cicadas of the Indian subcontinent”: http://mnd.ly/1OFGZc1. Original species descriptions without copyright restrictions can be downloaded from the NHM data portal: http://dx.doi.org/10.5519/0028307.

## Data resources

The bibliography is publically accessible on Mendeley: http://mnd.ly/1OFGZc1

The species descriptions are available on the NHM data portal: http://dx.doi.org/10.5519/0028307

The checklist files for each individual country are available on the NHM data portal: http://dx.doi.org/10.5519/0028307

The above files and information, along with images of specimens examined are also available on the Cicadas of India website: http://www.indiancicadas.org/

## Checklists

### Checklist 1 - Species recorded from India, Bangladesh, Bhutan, Myanmar, Nepal and Sri Lanka

#### Abroma apicalis

Ollenbach, 1929

Abroma apicalis Ollenbach, 1929

##### Materials

- **Type status:** Holotype. **Occurrence:** individualCount: 1; sex: female; **Taxon:** scientificName: Abroma apicalis Ollenbach, 1929; **Location:** continent: Asia; country: India; locality: Dehra Dun; **Record Level:** institutionCode: IFRI; basisOfRecord: PreservedSpecimen

##### Distribution

[Metcalf, 1963] Uttar Pradesh; India.

##### Notes

Authority: Ollenbach 1929

#### Abroma bengalensis

Distant, 1906

Abroma bengalensis Distant 1906

##### Materials

- **Type status:** Holotype. **Occurrence:** catalogNumber: BMNH(E) 1009605; individualCount: 1; sex: male; **Taxon:** scientificName: Abroma bengalensis Distant, 1906; **Location:** continent: Asia; country: India; locality: Kurseong, Bengal; **Record Level:** institutionCode: NHMUK; basisOfRecord: PreservedSpecimen

##### Distribution

[Metcalf, 1963] Bengal; India. [Duffels and van der Laan, 1985] Nepal.

##### Notes

Authority: Distant 1906c

#### Abroma maculicollis

(Guérin-Méneville, 1838)

Cicada maculicollis Guérin-Méneville, 1838

##### Materials

- **Type status:** Holotype. **Taxon:** scientificName: Abroma maculicollis (Guérin-Méneville, 1838); **Location:** continent: Asia; country: India; locality: Bengal; **Record Level:** basisOfRecord: PreservedSpecimen
- **Type status:** Other material. **Occurrence:** catalogNumber: BMNH(E) 1009606; individualCount: 1; sex: female; **Taxon:** scientificName: Abroma maculicollis (Guérin-Méneville, 1838); **Location:** continent: Asia; country: Malaysia; locality: Kuala Lumpur; **Event:** eventDate: 13/04/1932; **Record Level:** institutionCode: NHMUK; basisOfRecord: PreservedSpecimen

##### Distribution

[Metcalf, 1963] Bengal; India; Malay Peninsula; Ceylon; Borneo; Malay States; Perak; Malacca; Singapore Island; Johore; Penang; Uttar Pradesh; Malaya. [Sanborn, 2014] Borneo, Sabah, Bengal, China, Sumatra, Java, India, Peninsular Malaysia, Sarawak, Manipur, West Bengal, Sri Lanka, Malay Peninsula.

##### Notes

Authority: Guérin-Méneville 1838

#### Abroma nubifurca

(Walker, 1858)

Cicada nubifurca Walker, 1858
Cicada apicalis Kirkaldy, 1891

##### Materials

- **Type status:** Holotype. **Occurrence:** catalogNumber: BMNH(E) 1009607; recordedBy: R. Templeton; sex: female; **Taxon:** scientificName: Abroma nubifurca (Walker, 1858); **Location:** continent: Asia; country: Sri Lanka; locality: Ceylon; **Record Level:** institutionCode: NHMUK; basisOfRecord: PreservedSpecimen

##### Distribution

[Metcalf, 1963] Ceylon. [Duffels and van der Laan, 1985] Ceylon. [Sanborn, 2014] Ceylon.

##### Notes

Authority: Walker 1858b

#### Aetanna tigroides

(Walker, 1858)

Dundubia tigroides Walker, 1858

##### Materials

- **Type status:** Holotype. **Occurrence:** catalogNumber: BMNH(E) 1009432; individualCount: 1; sex: male; **Taxon:** scientificName: Aetanna tigroides (Walker, 1858); **Location:** continent: Asia; country: India; locality: Hindostan; **Record Level:** institutionCode: NHMUK; basisOfRecord: PreservedSpecimen
- **Type status:** Other material. **Occurrence:** catalogNumber: BMNH(E) 1009433; recordedBy: P.W. Mackinnon; individualCount: 1; sex: male; **Taxon:** scientificName: Aetanna tigroides (Walker, 1858); **Location:** continent: Asia; country: India; locality: Dehra Dun; **Record Level:** institutionCode: NHMUK; basisOfRecord: PreservedSpecimen

##### Distribution

[Metcalf, 1963] Hindustan; Tenasserim; India; Laos; Borneo; Banguey.

##### Notes

Authority: Walker 1858a; Species description states female, however Distant 1889a later figured a male. The holotype is likely a male as there is only one specimen in the NHMUK collection.

#### Ambragaeana stellata stellata

(Walker, 1858)

Huechys stellata Walker, 1858

##### Materials

- **Type status:** Holotype. **Occurrence:** catalogNumber: BMNH(E) 1009594; individualCount: 1; sex: female; **Taxon:** scientificName: Ambragaeana stellata stellata (Walker, 1858); **Location:** continent: Asia; country: India; locality: Hindostan; **Record Level:** institutionCode: NHMUK; basisOfRecord: PreservedSpecimen
- **Type status:** Other material. **Occurrence:** catalogNumber: BMNH(E) 1009595; recordedBy: William Doherty; individualCount: 1; sex: male; **Taxon:** scientificName: Ambragaeana stellata stellata (Walker, 1858); **Location:** continent: Asia; country: India; locality: Margherita, Assam; **Record Level:** institutionCode: NHMUK; basisOfRecord: PreservedSpecimen

##### Distribution

[Distant, 1889/92] Continental India: Assam; Khasi Hills. [Metcalf, 1963] Hindustan; India; Assam; Northern India. [Sanborn, 2014] Thailand, Hindustan, India, China.

##### Notes

Authority: Walker 1858a

#### Ambragaeana stellatavar.a

(Distant, 1892)

Gaeana stellata var. a Distant, 1892

##### Materials

- **Type status:** Holotype. **Occurrence:** recordedBy: William Doherty; sex: female; **Taxon:** scientificName: Ambragaeana stellata var. a (Distant, 1892); **Location:** continent: Asia; country: India; locality: Margherita, Assam; **Record Level:** basisOfRecord: PreservedSpecimen

##### Distribution

[Metcalf, 1963] Assam; Northern India.

##### Notes

Authority: Distant 1892c

#### Angamiana aetherea

Distant, 1890

Angamiana aetherea Distant, 1890

##### Materials

- **Type status:** Syntype. **Occurrence:** catalogNumber: BMNH(E) 1009378; recordedBy: William Doherty; individualCount: 1; sex: male; **Taxon:** scientificName: Angamiana aetherea Distant, 1890; **Location:** continent: Asia; country: India; locality: Naga Hills; **Record Level:** institutionCode: NHMUK; basisOfRecord: PreservedSpecimen
- **Type status:** Syntype. **Occurrence:** catalogNumber: BMNH(E) 1009825; recordedBy: William Doherty; individualCount: 1; sex: female; **Taxon:** scientificName: Angamiana aetherea Distant, 1890; **Location:** continent: Asia; country: India; locality: Naga Hills; **Record Level:** institutionCode: NHMUK; basisOfRecord: PreservedSpecimen
- **Type status:** Syntype. **Occurrence:** catalogNumber: BMNH(E) 1009823; recordedBy: William Doherty; individualCount: 1; sex: female; **Taxon:** scientificName: Angamiana aetherea Distant, 1890; **Location:** continent: Asia; country: India; locality: Naga Hills; **Record Level:** institutionCode: NHMUK; basisOfRecord: PreservedSpecimen
- **Type status:** Syntype. **Occurrence:** recordedBy: William Doherty; individualCount: 1; sex: male; **Taxon:** scientificName: Angamiana aetherea Distant, 1890; **Location:** continent: Asia; country: India; locality: Naga Hills; **Record Level:** institutionCode: NHMUK; basisOfRecord: PreservedSpecimen

##### Distribution

[Metcalf, 1963] Assam; India. [Sanborn, 2014] Assam, Continental India.

##### Notes

Authority: Distant 1890b; Unsure of number of specimens in species description, NHMUK has 2 male and 2 female specimens matching type description.

#### Balinta delinenda

(Distant, 1888)

Gaeana delinenda Distant, 1888

##### Materials

- **Type status:** Syntype. **Occurrence:** catalogNumber: BMNH(E) 1009597; individualCount: 1; sex: male; **Taxon:** scientificName: Balinta delinenda (Distant, 1888); **Location:** continent: Asia; country: India; locality: Cochin; **Record Level:** institutionCode: NHMUK; basisOfRecord: PreservedSpecimen
- **Type status:** Syntype. **Occurrence:** individualCount: 1; sex: male; **Taxon:** scientificName: Balinta delinenda (Distant, 1888); **Location:** continent: Asia; country: Bangladesh; locality: Silhet; **Record Level:** institutionCode: NHMUK; basisOfRecord: PreservedSpecimen

##### Distribution

[Metcalf, 1963] Assam; Cochin; India; Cochin China; Indochina. [Sanborn, 2014] Vietnam, India.

##### Notes

Authority: Distant 1888a; Two specimens used in species description, however only one is present in the NHMUK collection, an additional specimen can be found under "Balinta sp" which may be the other syntype, however this requires additional study.

#### Balinta octonotata octonotata

(Westwood, 1845)

Cicada octonotata Westwood, 1845
Huechys picta Walker, 1858

##### Materials

- **Type status:** Syntype. **Occurrence:** recordedBy: Mr Robinson; sex: male; **Taxon:** scientificName: Balinta octonotata octonotata (Westwood, 1845); **Location:** continent: Asia; country: India; locality: Assam; **Record Level:** institutionCode: OUMNH; basisOfRecord: PreservedSpecimen
- **Type status:** Syntype. **Occurrence:** recordedBy: Mr Robinson; sex: female; **Taxon:** scientificName: Balinta octonotata octonotata (Westwood, 1845); **Location:** continent: Asia; country: India; locality: Assam; **Record Level:** institutionCode: OUMNH; basisOfRecord: PreservedSpecimen
- **Type status:** Other material. **Occurrence:** catalogNumber: BMNH(E) 1009598; recordedBy: Moller; individualCount: 1; sex: male; **Taxon:** scientificName: Balinta octonotata octonotata (Westwood, 1845); **Location:** continent: Asia; country: India; locality: Sikkim; **Event:** eventDate: ??/??/1905; **Record Level:** institutionCode: NHMUK; basisOfRecord: PreservedSpecimen

##### Distribution

[Distant, 1906] India: Sikkim; Margherita (Assam). Burma: Upper Regions. [Metcalf, 1963] Assam; Java; Sikkim; India; Burma. [Duffels and van der Laan, 1985] Bhutan; Nepal. [Sanborn, 2014] Assam.

##### Notes

Authority: Westwood 1845

#### Balinta octonotatavar.a

(Westwood, 1845)

Gaeana octonotata var. a Distant, 1892

##### Materials

- **Type status:** Syntype. **Occurrence:** recordedBy: William Doherty; **Taxon:** scientificName: Balinta octonotata var. a Distant, 1892; **Location:** continent: Asia; country: India; locality: Assam, Margherita; **Record Level:** basisOfRecord: PreservedSpecimen
- **Type status:** Syntype. **Occurrence:** recordedBy: C.T. Bingham; **Taxon:** scientificName: Balinta octonotata var. a Distant, 1892; **Location:** continent: Asia; country: Myanmar; locality: Burma; **Record Level:** basisOfRecord: PreservedSpecimen

##### Distribution

[Metcalf, 1963] Assam; Burma.

##### Notes

Authority: Westwood 1845

#### Balinta octonotatavar.b

(Westwood, 1845)

Gaeana octonotata var. b Distant, 1892

##### Materials

- **Type status:** Holotype. **Taxon:** scientificName: Balinta octonotata var. b Distant, 1892; **Location:** continent: Asia; country: Myanmar; locality: Upper Burma; **Record Level:** basisOfRecord: PreservedSpecimen

##### Distribution

[Metcalf, 1963] Upper Burma.

##### Notes

Authority: Westwood 1845

#### Balinta sanguiniventris

Ollenbach, 1929

Balinta sanguiniventris Ollenbach, 1929

##### Materials

- **Type status:** Holotype. **Occurrence:** recordedBy: H. Inglis; individualCount: 1; sex: male; **Taxon:** scientificName: Balinta sanguiniventris Ollenbach, 1929; **Location:** continent: Asia; country: India; locality: Murphuleni, Assam; **Record Level:** institutionCode: IFRI; basisOfRecord: PreservedSpecimen

##### Distribution

[Metcalf, 1963] Assam; India.

##### Notes

Authority: Ollenbach 1929

#### Balinta tenebricosa tenebricosa

(Distant, 1888)

Gaeana tenebricosa Distant, 1888

##### Materials

- **Type status:** Holotype. **Occurrence:** catalogNumber: NHMUK 010214244; recordedBy: Leonardo Fea; individualCount: 1; sex: male; **Taxon:** scientificName: Balinta tenebricosa tenebricosa (Distant, 1888); **Location:** continent: Asia; country: Myanmar; locality: Moolay River, Teinzo, Burma; **Event:** eventDate: ??/05/1886; **Record Level:** institutionCode: NHMUK; basisOfRecord: PreservedSpecimen
- **Type status:** Other material. **Occurrence:** catalogNumber: BMNH(E) 1009599; recordedBy: R.V. de Salvaza; individualCount: 1; sex: male; **Taxon:** scientificName: Balinta tenebricosa tenebricosa (Distant, 1888); **Location:** continent: Asia; country: Vietnam; locality: Tonkin; **Event:** eventDate: ??/06/1917; **Record Level:** institutionCode: NHMUK; basisOfRecord: PreservedSpecimen

##### Distribution

[Metcalf, 1963] Burma; Laos. [Sanborn, 2014] China, Guangxi, Burma, Thailand, Laos, Vietnam.

##### Notes

Authority: Distant 1888d

#### Balinta tenebricosavar.a

(Distant, 1888)

Gaeana tenebricosa var. a Distant, 1892

##### Materials

- **Type status:** Holotype. **Taxon:** scientificName: Balinta tenebricosa var. a Distant, 1892; **Location:** continent: Asia; country: Myanmar; locality: Burma; **Record Level:** basisOfRecord: PreservedSpecimen

##### Distribution

[Metcalf, 1963] Burma.

##### Notes

Authority: Distant 1888d

#### Basa singularis

(Walker, 1858)

Dundubia singularis Walker, 1858

##### Materials

- **Type status:** Holotype. **Occurrence:** catalogNumber: BMNH(E) 1009538; sex: male; **Taxon:** scientificName: Basa singularis (Walker, 1858); **Location:** continent: Asia; country: India; locality: E. India, Hindostan; **Record Level:** institutionCode: NHMUK; basisOfRecord: PreservedSpecimen
- **Type status:** Other material. **Occurrence:** catalogNumber: BMNH(E) 1009539; recordedBy: J.G. Pilcher; individualCount: 1; sex: male; **Taxon:** scientificName: Basa singularis (Walker, 1858); **Location:** continent: Asia; country: India; locality: Sikkim; verbatimElevation: 4000 ft; **Event:** eventDate: ??/07/1895; **Record Level:** institutionCode: NHMUK; basisOfRecord: PreservedSpecimen

##### Distribution

[Distant, 1889/92] India: Darjeeling. [Metcalf, 1963] Hindustan; India; Bengal; Sikkim. [Sanborn, 2014] India.

##### Notes

Authority: Walker 1858b

#### Bijaurana sita

Distant, 1912

Bijaurana sita Distant, 1912

##### Materials

- **Type status:** Holotype. **Occurrence:** catalogNumber: BMNH(E) 1009608; individualCount: 1; sex: male; **Taxon:** scientificName: Bijaurana sita Distant, 1912; **Location:** continent: Asia; country: India; locality: United Provinces; **Record Level:** institutionCode: NHMUK; basisOfRecord: PreservedSpecimen

##### Distribution

[Metcalf, 1963] United Provinces; India.

##### Notes

Authority: Distant 1912c

#### Bijaurana typica

Distant, 1912

Bijaurana typica Distant, 1912

##### Materials

- **Type status:** Syntype. **Occurrence:** catalogNumber: BMNH(E) 1009609; sex: male; **Taxon:** scientificName: Bijaurana typica Distant, 1912; **Location:** continent: Asia; country: Nepal; locality: Bijaura; **Event:** eventDate: 28-29/04/1907; **Record Level:** institutionCode: NHMUK; basisOfRecord: PreservedSpecimen
- **Type status:** Syntype. **Taxon:** scientificName: Bijaurana typica Distant, 1912; **Location:** continent: Asia; country: Nepal; locality: Nepal; **Record Level:** institutionCode: NZSI; basisOfRecord: PreservedSpecimen

##### Distribution

[Metcalf, 1963] Nepal; Natal. [Duffels and van der Laan, 1985] Nepal.

##### Notes

Authority: Distant 1912c; Multiple specimens (number unknown) used in species description.

#### Calcagninus divaricatus

Bliven, 1964

Calcagninus divaricatus Bliven, 1964

##### Materials

- **Type status:** Holotype. **Occurrence:** recordedBy: P. Susai Nathan; individualCount: 1; sex: male; **Taxon:** scientificName: Calcagninus divaricatus Bliven, 1964; **Location:** continent: Asia; country: India; locality: Singara, Nilgiri Hills, South India; verbatimElevation: 3500 ft; **Event:** eventDate: 8/05/1963; **Record Level:** institutionCode: CAS; basisOfRecord: PreservedSpecimen

##### Distribution

[Duffels and van der Laan, 1985] South India.

##### Notes

Authority: Bliven 1964

#### Calcagninus nilgirensis

(Distant, 1887)

Leptopsaltria nilgirensis Distant, 1887

##### Materials

- **Type status:** Holotype. **Occurrence:** catalogNumber: BMNH(E) 1009564; recordedBy: G.F. Hampson; individualCount: 1; sex: female; **Taxon:** scientificName: Calcagninus nilgirensis (Distant, 1887); **Location:** continent: Asia; country: India; locality: Nilgiri Hills, northern slopes (Madras); verbatimElevation: 5000 ft; **Event:** eventDate: 2/06/1887; **Record Level:** institutionCode: NHMUK; basisOfRecord: PreservedSpecimen

##### Distribution

[Distant, 1889/92] Continental India: Neelgiri Hills (northern slopes, 5000 ft). [Metcalf, 1963] Madras; India.

##### Notes

Authority: Distant 1887b

#### Calcagninus picturatus

(Distant, 1888)

Leptopsaltria picturatus Distant, 1888

##### Materials

- **Type status:** Holotype. **Occurrence:** catalogNumber: BMNH(E) 1009563; recordedBy: G.F. Hampson; individualCount: 1; sex: male; **Taxon:** scientificName: Calcagninus picturatus (Distant, 1888); **Location:** continent: Asia; country: India; locality: Nilgiri Hills, northern slopes (Madras); verbatimElevation: 5000 ft; **Event:** eventDate: 28/06/1887; **Record Level:** institutionCode: NHMUK; basisOfRecord: PreservedSpecimen

##### Distribution

[Distant, 1889/92] Continental India: Neelgiri Hills (northern slopes, 5000 ft). [Metcalf, 1963] Madras; India.

##### Notes

Authority: Distant 1888c

#### Callogaeana annamensisvar.b

(Distant, 1892)

Gaeana annamensis var. b Distant, 1892
Gaeana festiva var. b Distant, 1892

##### Materials

- **Type status:** Holotype. **Occurrence:** recordedBy: A.W. Chennell; **Taxon:** scientificName: Callogaeana annamensis var. b (Distant, 1892); **Location:** continent: Asia; country: India; locality: south of Brahmapootra, Assam; **Record Level:** basisOfRecord: PreservedSpecimen

##### Distribution

[Metcalf, 1963] Assam.

##### Notes

Authority: Distant 1892c

#### Callogaeana festiva festiva

(Fabricius, 1803)

Tettigonia festiva Fabricius, 1803
Cicada thalassina Percheron, 1838 (nec Germar, 1830)
Cicada percheronii Guerin-Meneville, 1844

##### Materials

- **Type status:** Holotype. **Occurrence:** recordedBy: D. Daldorff; **Taxon:** scientificName: Callogaeana festiva festiva (Fabricius, 1803); **Location:** continent: Asia; country: Indonesia; locality: Sumatra; **Record Level:** institutionCode: MZLU; basisOfRecord: PreservedSpecimen
- **Type status:** Other material. **Occurrence:** catalogNumber: BMNH(E) 1009591; individualCount: 1; sex: female; **Taxon:** scientificName: Callogaeana festiva festiva (Fabricius, 1803); **Location:** continent: Asia; country: India; locality: Sikkim; **Record Level:** institutionCode: NHMUK; basisOfRecord: PreservedSpecimen

##### Distribution

[Distant, 1889/92] Continental India: Sikkim; Darjeeling. [Metcalf, 1963] Sumatra; India; Bengal; Malabar; Asia; Cuba (?); Eastern India; Assam; Sikkim; Molucca Islands; Laos; Amboina; Malay Archipelago; Amboina (?); Malay Peninsula; East Indies. [Duffels and van der Laan, 1985] Bhutan. [Sanborn, 2014] China, Hunan, Guangxi, Yunnan, Indonesia, Sikkim, India, Thailand, Sumatra, Laos, Malaysia, Indochina, Bhutan, Southeast Asia.

##### Notes

Authority: Fabricius 1803

#### Champaka spinosa

(Fabricius, 1787)

Tettigonia spinosa Fabricius, 1787
Tettigonia bispinosa Gmelin, 1789 (nom. nov. pro.)
Cosmopsaltria abdulla Distant, 1881
Platylomia maculata Liu, 1940

##### Materials

- **Type status:** Holotype. **Taxon:** scientificName: Champaka spinosa (Fabricius, 1787); **Location:** continent: Asia; country: Indonesia; locality: Sumatra; **Record Level:** institutionCode: NHMUK; basisOfRecord: PreservedSpecimen

##### Distribution

[Metcalf, 1963] Sumatra; New Guinea; Madras; Philippine Islands; Eastern India; Japan; Singapore Island; Penang; India; Malay Peninsula; Borneo; British North Borneo; Kyushu; Oriental Region; Sarawak; Burma; Assam; Sikkim; Tenasserim; Northern Borneo; Malaya; Malacca; Perak; Johore; Southern Japan; Japan (?); Kyushu (?); Malay Archipelago. [Sanborn, 2014] Malaysia, Borneo, Indonesia, Krakataus, Sarawak, Sabah, Peninsular Malaysia, Sumatra, Philippines, Singapore, New Guinea, Thailand, West Malaysia, East Malaysia, Brunei, Kalimantan, Java, India?, New Guinea, Vietnam, Thailand, Luzon.

##### Notes

Authority: Fabricius 1787

#### Chremistica germana

(Distant, 1888)

Cicada germana Distant, 1888

##### Materials

- **Type status:** Holotype. **Occurrence:** recordedBy: Leonardo Fea; sex: male; **Taxon:** scientificName: Chremistica germana (Distant, 1888); **Location:** continent: Asia; country: Myanmar; locality: Teinzo, Burma; **Record Level:** institutionCode: MSNG; basisOfRecord: PreservedSpecimen

##### Distribution

[Metcalf, 1963] Burma; Siam; Malay States; Penang; Malay Peninsula; Borneo; Sumatra; Java; Mentawai Island; Siberut; India. [Sanborn, 2014] Sarawak, Burma, Peninsular Malaysia, Siam, Sumatra, India, Southeast Asia, Indonesia, Thailand.

##### Notes

Authority: Distant 1888d. Metcalf (1963) listed India in reference Burma being part of "British India". The type locality (Teinzo) is within modern day Myanmar and the species has not yet been recorded from modern day India, however the species may be present on the India/Myanmar border.

#### Chremistica mixta

(Kirby, 1891)

Dundubia mixta Kirby, 1891

##### Materials

- **Type status:** Holotype. **Occurrence:** individualCount: 1; sex: female; **Taxon:** scientificName: Chremistica mixta (Kirby, 1891); **Location:** locality: Locality unknown; **Record Level:** basisOfRecord: PreservedSpecimen

##### Distribution

[Metcalf, 1963] Ceylon. [Duffels and van der Laan, 1985] Ceylon. [Sanborn, 2014] Sri Lanka.

##### Notes

Authority: Kirby 1891

#### Chremistica ribhoi

Hajong & Yaakop, 2013

Chremistica ribhoi Hajong & Yaakop, 2013

##### Materials

- **Type status:** Holotype. **Occurrence:** individualCount: 1; sex: male; **Taxon:** scientificName: Chremistica ribhoi Hajong & Yaakop, 2013; **Location:** continent: Asia; country: India; locality: Near Siden village, North-East India; verbatimElevation: 432 m; **Event:** eventDate: 9/06/2006; **Record Level:** institutionCode: NZSI; basisOfRecord: PreservedSpecimen
- **Type status:** Paratype. **Occurrence:** individualCount: 23; sex: male; **Taxon:** scientificName: Chremistica ribhoi Hajong & Yaakop, 2013; **Location:** continent: Asia; country: India; locality: Lailad, near Nongkyllem Wildlife Sanctuary; verbatimElevation: 416; **Record Level:** institutionCode: ERC-ZSI; basisOfRecord: PreservedSpecimen
- **Type status:** Paratype. **Occurrence:** individualCount: 7; sex: female; **Taxon:** scientificName: Chremistica ribhoi Hajong & Yaakop, 2013; **Location:** continent: Asia; country: India; locality: Lailad, near Nongkyllem Wildlife Sanctuary; verbatimElevation: 416; **Record Level:** institutionCode: ERC-ZSI; basisOfRecord: PreservedSpecimen

##### Distribution

[Sanborn, 2014] Cooch Behar, Alipurduar, Kalingpong, N. Paschimbanga, NE India; Guwahati, Assam, NE India; Ri Bhoi District, Meghalaya, NE India.

##### Notes

Authority: Hajong and Yaakop 2013; Species description included 1 male holotype, 23 male and 7 female paratypes.

#### Chremistica seminiger

(Distant, 1909)

Rihana seminiger Distant, 1909

##### Materials

- **Type status:** Holotype. **Occurrence:** recordedBy: H.L. Andrewes; individualCount: 1; sex: male; **Taxon:** scientificName: Chremistica seminiger (Distant, 1909); **Location:** continent: Asia; country: India; locality: Nilgiri Hills; **Record Level:** institutionCode: NHMUK; basisOfRecord: PreservedSpecimen

##### Distribution

[Metcalf, 1963] Madras; Nilgiri Hills; India. [Sanborn, 2014] India.

##### Notes

Authority: Distant 1909

#### Cicada conspurcata

(Fabricius, 1777)

Tettigonia conspurcata Fabricius, 1777

##### Materials

- **Type status:** Holotype. **Occurrence:** recordedBy: Dr Fothergill; **Taxon:** scientificName: Cicada conspurcata (Fabricius, 1777); **Location:** continent: Asia; country: India; **Record Level:** basisOfRecord: PreservedSpecimen

##### Distribution

[Metcalf, 1963] India.

##### Notes

Authority: Fabricius 1777

#### Cicada olivierana

Metcalf, 1963

Cicada olivierana Metcalf, 1963
Cicada ferruginea Olivier, 1790 (nec Fabricius, 1787)

##### Materials

- **Type status:** Holotype. **Taxon:** scientificName: Cicada olivierana Metcalf, 1963; **Location:** continent: Asia; locality: Indes Orientales; **Record Level:** basisOfRecord: PreservedSpecimen

##### Distribution

[Metcalf, 1963] East Indies; India.

##### Notes

Authority: Metcalf 1963; type distribution only states "indes Orientales" (i.e. East Indies) and as this encompassed a large area of Asia it cannot be confirmed or denied as being from India. Metcalf (1963) stated India in reference to various lists and catalogues which all referred to the type specimen as from India.

#### Cicadatra acberi

(Distant, 1888)

Tibicen acberi Distant, 1888

##### Materials

- **Type status:** Holotype. **Occurrence:** catalogNumber: BMNH(E) 1009573; recordedBy: Leech; individualCount: 1; sex: male; **Taxon:** scientificName: Cicadatra acberi (Distant, 1888); **Location:** continent: Asia; country: India; locality: Cashmere Valley; verbatimElevation: 6300 ft; **Record Level:** institutionCode: NHMUK; basisOfRecord: PreservedSpecimen

##### Distribution

[Metcalf, 1963] Kashmir, India. [Sanborn, 2014] Pakistan, India.

##### Notes

Authority: Distant 1888c

#### Cicadatra anoea

(Walker, 1850)

Cicada anoea Walker, 1850

##### Materials

- **Type status:** Holotype. **Occurrence:** sex: male; **Taxon:** scientificName: Cicadatra anoea (Walker, 1850); **Location:** continent: Asia; country: India; locality: North Bengal; **Record Level:** basisOfRecord: PreservedSpecimen

##### Distribution

[Metcalf, 1963] Bengal; India; Northern Bengal; Afghanistan.

##### Notes

Authority: Walker 1850

#### Cicadatra gingat

China, 1926

Cicadatra gingat China, 1926

##### Materials

- **Type status:** Holotype. **Occurrence:** recordedBy: E.B. Howell; individualCount: 1; sex: male; **Taxon:** scientificName: Cicadatra gingat China, 1926; **Location:** continent: Asia; country: Pakistan; locality: Razmak, Waziristan; verbatimElevation: 6000 - 7000 ft; **Event:** eventDate: ??/06/1926; **Record Level:** basisOfRecord: PreservedSpecimen

##### Distribution

[Metcalf, 1963] Northwestern Frontier Province; Northwestern India. [Sanborn, 2014] Pakistan, India.

##### Notes

Authority: China 1926

#### Cicadatra inconspicua

Distant, 1912

Cicadatra inconspicua Distant, 1912

##### Materials

- **Type status:** Holotype. **Occurrence:** catalogNumber: BMNH(E) 1009572; individualCount: 1; sex: male; **Taxon:** scientificName: Cicadatra inconspicua Distant, 1912; **Location:** continent: Asia; country: India; locality: Mhow; **Event:** eventDate: ??/??/1905; **Record Level:** institutionCode: NHMUK; basisOfRecord: PreservedSpecimen

##### Distribution

[Metcalf, 1963] Central Provinces; India.

##### Notes

Authority: Distant 1912a

#### Cicadatra raja

Distant, 1906

Cicadatra raja Distant, 1906

##### Materials

- **Type status:** Holotype. **Occurrence:** catalogNumber: BMNH(E) 1009568; recordedBy: P.W. Mackinnon; individualCount: 1; sex: male; **Taxon:** scientificName: Cicadatra raja Distant, 1906; **Location:** continent: Asia; country: India; locality: Aglar Valley (Jumna River), Masuri; verbatimElevation: 3000 ft; **Event:** eventDate: 18/06/1904; **Record Level:** institutionCode: NHMUK; basisOfRecord: PreservedSpecimen

##### Distribution

[Metcalf, 1963] Northwestern India. [Sanborn, 2014] Pakistan, India.

##### Notes

Authority: Distant 1906d

#### Cicadatra sankara

(Distant, 1904)

Tibicen sankara Distant, 1904

##### Materials

- **Type status:** Syntype. **Occurrence:** catalogNumber: BMNH(E) 1009569; recordedBy: P.W. Mackinnon; sex: male; **Taxon:** scientificName: Cicadatra sankara (Distant, 1904); **Location:** continent: Asia; country: India; locality: Chamasari; verbatimElevation: 5000 ft; **Event:** eventDate: ??/05/1903; **Record Level:** institutionCode: NHMUK; basisOfRecord: PreservedSpecimen
- **Type status:** Syntype. **Occurrence:** recordedBy: P.W. Mackinnon; sex: female; **Taxon:** scientificName: Cicadatra sankara (Distant, 1904); **Location:** continent: Asia; country: India; locality: Chamasari; verbatimElevation: 5000 ft; **Event:** eventDate: ??/05/1903; **Record Level:** basisOfRecord: PreservedSpecimen

##### Distribution

[Metcalf, 1963] India; Baluchistan; Oriental Region; Northern India; Uttar Pradesh. [Sanborn, 2014] Pakistan, India.

##### Notes

Authority: Distant 1904b

#### Cicadatra walkeri

Metcalf, 1963

Cicadatra walkeri Metcalf, 1963
Cicada striata Walker, 1850 (nec Linnaeus, 1758)

##### Materials

- **Type status:** Holotype. **Occurrence:** catalogNumber: BMNH(E) 1009570; sex: female; **Taxon:** scientificName: Cicadatra walkeri Metcalf, 1963; **Location:** locality: Locality unknown; **Record Level:** institutionCode: NHMUK; basisOfRecord: PreservedSpecimen

##### Distribution

[Distant, 1889/92] India: Quetta; North Bengal. [Metcalf, 1963] India; Quetta; Bengal; Pakistan; Oriental Region; Baluchistan. [Sanborn, 2014] Pakistan, India.

##### Notes

Authority: Metcalf 1963

#### Cicadatra xantes

(Walker, 1850)

Cicada xantes Walker, 1850
Cicada subvenosa Walker, 1858

##### Materials

- **Type status:** Holotype. **Occurrence:** catalogNumber: BMNH(E) 1009566; individualCount: 1; sex: male; **Taxon:** scientificName: Cicadatra xantes (Walker, 1850); **Location:** continent: Asia; country: India; locality: N. India; **Record Level:** institutionCode: NHMUK; basisOfRecord: PreservedSpecimen

##### Distribution

[Metcalf, 1963] India; Hindustan; Northern India; Oriental Region; Sind; Peshawar. [Sanborn, 2014] Pakistan, India.

##### Notes

Authority: Walker 1850

#### Cicadetta intermedia

(Ollenbach, 1929)

Melampsalta intermedia Ollenbach, 1929

##### Materials

- **Type status:** Holotype. **Occurrence:** recordedBy: B.M. Bhatia; individualCount: 1; sex: female; **Taxon:** scientificName: Cicadetta intermedia (Ollenbach, 1929); **Location:** continent: Asia; country: India; locality: Kotkhai, Simla Hill States, Punjab; **Event:** eventDate: ??/05/1924; **Record Level:** institutionCode: IFRI; basisOfRecord: PreservedSpecimen

##### Distribution

[Metcalf, 1963] Punjab; India.

##### Notes

Authority: Ollenbach 1929

#### Cicadetta minuta

(Ollenbach, 1929)

Melampsalta minuta Ollenbach, 1929

##### Materials

- **Type status:** Syntype. **Occurrence:** recordedBy: C.F.C. Beeson; individualCount: 1; sex: male; **Taxon:** scientificName: Cicadetta minuta (Ollenbach, 1929); **Location:** continent: Asia; country: India; locality: Kanasar, Chakrata, United Provinces; verbatimElevation: 5000 - 7000 ft; **Event:** eventDate: ??/06/1923; **Record Level:** institutionCode: IFRI; basisOfRecord: PreservedSpecimen
- **Type status:** Syntype. **Occurrence:** recordedBy: C.F.C. Beeson; individualCount: 1; sex: female; **Taxon:** scientificName: Cicadetta minuta (Ollenbach, 1929); **Location:** continent: Asia; country: India; locality: Kanasar, Chakrata, United Provinces; verbatimElevation: 5000 - 7000 ft; **Event:** eventDate: ??/06/1923; **Record Level:** institutionCode: IFRI; basisOfRecord: PreservedSpecimen

##### Distribution

[Metcalf, 1963] United Provinces; India; Uttar Pradesh.

##### Notes

Authority: Ollenbach 1929

#### Cryptotympana aquila

(Walker, 1850)

Fidicina aquila Walker, 1850

##### Materials

- **Type status:** Holotype. **Occurrence:** sex: female; **Taxon:** scientificName: Cryptotympana aquila (Walker, 1850); **Location:** continent: Asia; country: Korea; locality: Corea; **Record Level:** basisOfRecord: PreservedSpecimen

##### Distribution

[Metcalf, 1963] Korea, Borneo, Sumatra, Malay Peninsula, Tonkin, Malay States, Japan, Perak, Malacca, Singapore Island, Johore, Penang, Hong Kong Island, Oriental Region, Java, Sarawak, Malaya, Korea (?), Formosa, Philippine Islands. [Duffels and van der Laan, 1985] Taiwan, Malaysia, Korea. [Sanborn, 2014] Sumatra, Malay Peninsula, Borneo, Indo-China, Borneo, Thailand, Burma, Laos, Vietnam, Malaysia, Sarawak, China, Brunei, Korea, Sabah, Southeast Asia.

##### Notes

Authority: Walker 1850

#### Cryptotympana auropilosa

Hayashi, 1987

Cryptotympana auropilosa Hayashi, 1987

##### Materials

- **Type status:** Holotype. **Occurrence:** recordedBy: Archbald; individualCount: 1; sex: female; **Taxon:** scientificName: Cryptotympana auropilosa Hayashi, 1987; **Location:** continent: Asia; country: Myanmar; locality: Rangoon; **Record Level:** institutionCode: NHMUK; basisOfRecord: PreservedSpecimen

##### Distribution

[Sanborn, 2014] Burma.

##### Notes

Authority: Hayashi 1987a

#### Cryptotympana corvus

(Walker, 1850)

Fidicina corvus Walker, 1850
Fidicina invarians Walker, 1858

##### Materials

- **Type status:** Holotype. **Occurrence:** catalogNumber: BMNH(E) 1009382; individualCount: 1; sex: male; **Taxon:** scientificName: Cryptotympana corvus (Walker, 1850); **Location:** continent: Asia; country: Bangladesh; locality: Silhet; **Record Level:** institutionCode: NHMUK; basisOfRecord: PreservedSpecimen
- **Type status:** Other material. **Occurrence:** individualCount: 1; sex: male; **Taxon:** scientificName: Cryptotympana corvus (Walker, 1850); **Location:** continent: Asia; country: India; locality: Sikkim; **Record Level:** institutionCode: NHMUK; basisOfRecord: PreservedSpecimen

##### Distribution

[Distant, 1889/92] Continental India: Sikkim; Darjeeling; Assam; Sylhet; Naga Hills; Neelgiri Hills (southern slopes). [Metcalf, 1963] Assam; Hindustan; East Bengal; India; Tonkin; Sikkim; Bengal; Eastern Himalayas; Madras; Uttar Pradesh. [Duffels and van der Laan, 1985] Himalaya. [Sanborn, 2014] China, Indo-China, Himalayas, India, Bangladesh, Bhutan, Nepal.

##### Notes

Authority: Walker 1850; Species description states female, however the labelled type specimen examined in the NHMUK is male.

#### Cryptotympana edwardsi

Kirkaldy, 1902

Cryptotympana edwardsi Kirkaldy, 1902

##### Materials

- **Type status:** Holotype. **Occurrence:** recordedBy: Edwards; sex: male; **Taxon:** scientificName: Cryptotympana edwardsi Kirkaldy, 1902; **Location:** continent: Asia; country: India; **Record Level:** basisOfRecord: PreservedSpecimen

##### Distribution

[Metcalf, 1963] India. [Sanborn, 2014] India.

##### Notes

Authority: Kirkaldy 1902

#### Cryptotympana exalbida

Distant, 1891

Cryptotympana exalbida Distant, 1891

##### Materials

- **Type status:** Syntype. **Occurrence:** catalogNumber: BMNH(E) 1009387; occurrenceRemarks: Voucher number corresponds to one specimen.; recordedBy: G. Hampson; individualCount: 4; sex: female; **Taxon:** scientificName: Cryptotympana exalbida Distant, 1891; **Location:** continent: Asia; country: India; locality: Nilgiri N. Slopes; verbatimElevation: 3500 ft; **Event:** eventDate: 25/05/1888; **Record Level:** institutionCode: NHMUK; basisOfRecord: PreservedSpecimen
- **Type status:** Other material. **Occurrence:** catalogNumber: BMNH(E) 1009386; occurrenceRemarks: Specimen not part of type series, see taxon notes.; individualCount: 1; sex: male; **Taxon:** scientificName: Cryptotympana exalbida Distant, 1891; **Location:** continent: Asia; country: Sri Lanka; locality: Oduchuddan; **Event:** eventDate: ??/05/1910; **Record Level:** institutionCode: NHMUK; basisOfRecord: PreservedSpecimen

##### Distribution

[Metcalf, 1963] India; Sikkim; Ceylon; Madras. [Duffels and van der Laan, 1985] Ceylon. [Sanborn, 2014] India, Sri Lanka.

##### Notes

Authority: Distant 1891; In the NHMUK there is a male specimen bearing a type label and a note from Broomfield dated 1978. This male specimen was described later by Distant (1913a). The type series includes four females from "Continental India: Neelgiri Hills, northern slopes, 3500 feet (Hampson-coll. Dist.)".

#### Cryptotympana insularis

Distant, 1887

Cryptotympana insularis Distant, 1887

##### Materials

- **Type status:** Lectotype. **Occurrence:** catalogNumber: BMNH(E) 1009379; recordedBy: Meldola; individualCount: 1; sex: male; **Taxon:** scientificName: Cryptotympana insularis Distant, 1887; **Location:** continent: Asia; country: India; locality: Port Blair, Andaman Islands; **Record Level:** institutionCode: NHMUK; basisOfRecord: PreservedSpecimen
- **Type status:** Paralectotype. **Occurrence:** catalogNumber: BMNH(E) 1009380; individualCount: 1; sex: female; **Taxon:** scientificName: Cryptotympana insularis Distant, 1887; **Location:** continent: Asia; country: India; locality: Port Blair, Andaman Islands; **Record Level:** institutionCode: NHMUK; basisOfRecord: PreservedSpecimen

##### Distribution

[Metcalf, 1963] Andaman Islands. [Sanborn, 2014] Andaman Islands.

##### Notes

Authority: Distant 1887a; Lectotype designated by Hayashi (1987b).

#### Cryptotympana intermedia

(Signoret, 1849)

Cicada intermedia Signoret, 1849

##### Materials

- **Type status:** Holotype. **Occurrence:** recordedBy: Patrie; sex: male; **Taxon:** scientificName: Cryptotympana intermedia (Signoret, 1849); **Location:** continent: Asia; country: Indonesia; locality: Java; **Record Level:** basisOfRecord: PreservedSpecimen
- **Type status:** Other material. **Occurrence:** individualCount: 1; sex: male; **Taxon:** scientificName: Cryptotympana intermedia (Signoret, 1849); **Location:** continent: Asia; country: India; locality: N. Bengal; **Record Level:** institutionCode: NHMUK; basisOfRecord: PreservedSpecimen

##### Distribution

[Metcalf, 1963] Java; Bengal; Tenasserim; Japan; China; India; Ceylon; Mysore; Malay States; Honshu; Kyushu; Ryukyu Islands; Oriental Region; Formosa; Sikkim; Malaya; Java (?); Ceylon (?); Northern Bengal; Madras; United Providences. [Duffels and van der Laan, 1985] Ceylon; Nepal. [Sanborn, 2014] India, Nepal, Burma, Himalayas.

##### Notes

Authority: Signoret 1849 Not likely to be found in Sri Lanka.

#### Cryptotympana limborgi

Distant, 1888

Cryptotympana limborgi Distant, 1888

##### Materials

- **Type status:** Holotype. **Occurrence:** catalogNumber: BMNH(E) 1009385; individualCount: 1; sex: male; **Taxon:** scientificName: Cryptotympana limborgi Distant, 1888; **Location:** continent: Asia; country: Myanmar; locality: Tenasserim; **Record Level:** institutionCode: NHMUK; basisOfRecord: PreservedSpecimen

##### Distribution

[Metcalf, 1963] Tenasserim; East Bengal; Upper Tenasserim. [Sanborn, 2014] Burma.

##### Notes

Authority: Distant 1888a

#### Cryptotympana mandarina

Distant, 1891

Cryptotympana mandarina Distant, 1891
Fidicina operculata Walker, 1850 (nom. nud.)
Cryptotympana mimica Distant, 1917

##### Materials

- **Type status:** Holotype. **Occurrence:** sex: male; **Taxon:** scientificName: Cryptotympana mandarina Distant, 1891; **Location:** continent: Asia; country: China; **Record Level:** basisOfRecord: PreservedSpecimen

##### Distribution

[Lee, 2014] China: Fujian, Guangdong, Hunan, Guangxi, Hainan, Sichuan, Guizhou, Yunnan, Tibet. Vietnam; Laos; Cambodia; Thailand; Myanmar. [Metcalf, 1963] Northern India; China; Tonkin; Hong Kong Island; Tenasserim; Hainan; Indochina; Formosa; Japan; Kiangsu; Kwangsi; Sikang; Kiangsi. [Sanborn, 2014] Hongkong, Hainan, Tenasserim, Tonkin, Formosa, China, Hunan, Hubei, Jiangsu, Jiangxi, Zhejiang, Fujian, Taiwan, Guangdong, Guangxi, Guizhou, Sichuan, Yunnan, Laos, Vietnam, India, Southeast Asia, Thailand, Myanmar, Cambodia, Indochina.

##### Notes

Authority: Distant 1891; Metcalf (1963) lists Northern India in reference to Atkinson (1884), however according to Distant (1891) "Mr. Atkinson* states that "the Indian Museum possesses a specimen" of C. operculata, Carreno, but this is probably erroneous, as he writes that the species was recorded from N. India, though Walker perhaps the greatest obscurantist who has yet appeared in entomology gave no locality to the specimens he identified under Carreno's name, without any reference to the place or manner of their description."

#### Cryptotympana recta

(Walker, 1850)

Fidicina recta Walker, 1850

##### Materials

- **Type status:** Holotype. **Occurrence:** catalogNumber: BMNH(E) 1009381; individualCount: 1; sex: female; **Taxon:** scientificName: Cryptotympana recta (Walker, 1850); **Location:** continent: Asia; country: Bangladesh; locality: Silhet; **Record Level:** institutionCode: NHMUK; basisOfRecord: PreservedSpecimen

##### Distribution

[Distant, 1889/92] Continental India: Sylhet; North Khasi Hills; Neelgiri Hills (south slopes). [Metcalf, 1963] Assam; East Bengal; India; China; Tonkin. [Sanborn, 2014] Sumatra, Malay Peninsula, Borneo, India, Indo-China, Bangladesh, China, Vietnam, Laos, Thailand, Sunda, Asia, Guangxi, Cambodia.

##### Notes

Authority: Walker 1850; The species description was based on a single female type specimen and the male of the species was later described in Distant (1879).

#### Cryptotympana vesta

(Distant, 1904)

Cicada vesta Distant, 1904

##### Materials

- **Type status:** Syntype. **Occurrence:** catalogNumber: BMNH(E) 1009384; recordedBy: R.M. Dixon; sex: male; **Taxon:** scientificName: Cryptotympana vesta (Distant, 1904); **Location:** continent: Asia; country: India; locality: Bombay; **Record Level:** institutionCode: NHMUK; basisOfRecord: PreservedSpecimen
- **Type status:** Other material. **Occurrence:** catalogNumber: BMNH(E) 1009383; individualCount: 1; sex: male; **Taxon:** scientificName: Cryptotympana vesta (Distant, 1904); **Location:** continent: Asia; country: India; locality: Assam; **Record Level:** institutionCode: NHMUK; basisOfRecord: PreservedSpecimen
- **Type status:** Syntype. **Occurrence:** recordedBy: R.M. Dixon; sex: female; **Taxon:** scientificName: Cryptotympana vesta (Distant, 1904); **Location:** continent: Asia; country: India; locality: Bombay; **Record Level:** basisOfRecord: PreservedSpecimen

##### Distribution

[Metcalf, 1963] Bombay; India; Siam. [Sanborn, 2014] Thailand, India.

##### Notes

Authority: Distant 1904b

#### Distantalna splendida splendida

(Distant, 1878)

Tosena splendida Distant, 1878

##### Materials

- **Type status:** Syntype. **Occurrence:** catalogNumber: BMNH(E) 1009376; recordedBy: A.W. Chennell; sex: male; **Taxon:** scientificName: Distantalna splendida splendida (Distant, 1878); **Location:** continent: Asia; country: India; locality: Naga Hills; verbatimElevation: 2000 - 6000 ft; **Record Level:** institutionCode: NHMUK; basisOfRecord: PreservedSpecimen
- **Type status:** Other material. **Occurrence:** individualCount: 1; sex: male; **Taxon:** scientificName: Distantalna splendida splendida (Distant, 1878); **Location:** continent: Asia; country: Myanmar; locality: Akyab (Sittwe); **Record Level:** institutionCode: NHMUK; basisOfRecord: PreservedSpecimen
- **Type status:** Syntype. **Occurrence:** recordedBy: A.W. Chennell; sex: female; **Taxon:** scientificName: Distantalna splendida splendida (Distant, 1878); **Location:** continent: Asia; country: India; locality: Khasia Hills; verbatimElevation: 4500 - 6000 ft; **Record Level:** basisOfRecord: PreservedSpecimen

##### Distribution

[Metcalf, 1963] Assam; India; Burma; Indochina; Naga Hills. [Sanborn, 2014] China, Yunnan, India, Burma, Thailand, Assam, Southeast Asia, Vietnam, Myanmar, Indochina.

##### Notes

Authority: Distant 1878b

#### Dundubia emanatura

Distant, 1889

Dundubia emanatura Distant, 1889

##### Materials

- **Type status:** Syntype. **Occurrence:** catalogNumber: BMNH(E) 1009526; recordedBy: G. Hampson; sex: female; **Taxon:** scientificName: Dundubia emanatura Distant, 1889; **Location:** continent: Asia; country: India; locality: Nilgiris, southern slopes; verbatimElevation: 3000 ft; **Event:** eventDate: 12/04/1888; **Record Level:** institutionCode: NHMUK; basisOfRecord: PreservedSpecimen
- **Type status:** Syntype. **Occurrence:** catalogNumber: BMNH(E) 1009527; recordedBy: E.F.T. Atkinson; sex: male; **Taxon:** scientificName: Dundubia emanatura Distant, 1889; **Location:** continent: Asia; country: India; locality: Karwar; **Record Level:** institutionCode: NHMUK; basisOfRecord: PreservedSpecimen

##### Distribution

[Metcalf, 1963] Bombay; Madras; India. [Duffels and van der Laan, 1985] India; Andaman Islands; Nicobar Islands. [Duffels and van der Laan, 1985] India; Andaman Islands; Nicobar Islands.

##### Notes

Authority: Distant 1889b

#### Dundubia ensifera

Bloem & Duffels, 1976

Dundubia ensifera Bloem & Duffels, 1976

##### Materials

- **Type status:** Holotype. **Occurrence:** recordedBy: E.F.T. Atkinson; individualCount: 1; sex: male; **Taxon:** scientificName: Dundubia ensifera Bloem & Duffels, 1976; **Location:** continent: Asia; country: India; locality: Sibs.[agar], Assam; **Record Level:** institutionCode: NHMUK; basisOfRecord: PreservedSpecimen
- **Type status:** Paratype. **Occurrence:** individualCount: 1; sex: male; **Taxon:** scientificName: Dundubia ensifera Bloem & Duffels, 1976; **Location:** continent: Asia; country: India; locality: Mtn. Khasi, Chenell; verbatimElevation: 1500 - 3000 ft; **Record Level:** institutionCode: NHMUK; basisOfRecord: PreservedSpecimen
- **Type status:** Paratype. **Occurrence:** individualCount: 1; sex: male; **Taxon:** scientificName: Dundubia ensifera Bloem & Duffels, 1976; **Location:** continent: Asia; country: Bangladesh; locality: Silhet; **Record Level:** institutionCode: NHMUK; basisOfRecord: PreservedSpecimen

##### Distribution

[Duffels and van der Laan, 1985] Pakistan (?); India. [Sanborn, 2014] Bangladesh.

##### Notes

Authority: Bloem and Duffels 1976

#### Dundubia feae

(Distant, 1892)

Cosmopsaltria feae Distant, 1892
Dundubia longina Distant, 1917

##### Materials

- **Type status:** Paralectotype. **Occurrence:** catalogNumber: BMNH(E) 1009530; occurrenceRemarks: Not Lectotype specimen; recordedBy: Leonardo Fea; individualCount: 1; sex: male; **Taxon:** scientificName: Dundubia feae (Distant, 1892); **Location:** continent: Asia; country: Myanmar; locality: Carin, Asciuii Ghecu, Tennasserim; verbatimElevation: 1400 - 1500 m; **Event:** eventDate: ??/03-04/1888; **Record Level:** institutionCode: NHMUK; basisOfRecord: PreservedSpecimen
- **Type status:** Lectotype. **Occurrence:** recordedBy: Leonardo Fea; individualCount: 1; sex: male; **Taxon:** scientificName: Dundubia feae (Distant, 1892); **Location:** continent: Asia; country: Myanmar; locality: Carin, Asciuii Ghecu, Tennasserim; verbatimElevation: 1400 - 1500 m; **Record Level:** institutionCode: MSNG; basisOfRecord: PreservedSpecimen

##### Distribution

[Metcalf, 1963] Burma; Kiangsu; China. [Sanborn, 2014] China, Soochow, Karennee, Laos, Thailand, Vietnam, Jiangsu, Unnan, Sichuan, Burma, Japan, India, Tenasserim, Laos, Tonkin, Guangxi, Hainan, Myanmar.

##### Notes

Authority: Distant 1892c; Lectotype was designated by Beuk (1996). There are an additional 3 male (2 MSNG; 1 BMNH) and 1 female (MSNG) paralectotypes, however only the examined material is included in this paper. Not from India: Sanborn (2014) states India in reference to Hua (2000) but no other records from India exist, it is likely the result of a mistranslation of Indo-China. The type locality (Carin Asciuii Ghecu, Tennasserim) is not near the India/Myanmar border.

#### Dundubia hastata

(Moulton, 1923)

Cosmopsaltria hastata Moulton, 1923

##### Materials

- **Type status:** Lectotype. **Occurrence:** catalogNumber: BMNH(E) 1009528; individualCount: 1; sex: male; **Taxon:** scientificName: Dundubia hastata (Moulton, 1923); **Location:** continent: Asia; country: Thailand; locality: W. Coast Siam; **Record Level:** institutionCode: NHMUK; basisOfRecord: PreservedSpecimen
- **Type status:** Paralectotype. **Occurrence:** catalogNumber: BMNH(E) 1009529; individualCount: 1; sex: female; **Taxon:** scientificName: Dundubia hastata (Moulton, 1923); **Location:** continent: Asia; country: Thailand; locality: W. Coast Siam; **Record Level:** institutionCode: NHMUK; basisOfRecord: PreservedSpecimen

##### Distribution

[Moulton, 1923]Terutau Island, Siam; North Khasia Hills; Assam; Indo-China. [Metcalf, 1963] Siam; Indochina; Assam; Malay Archipelago; Malay Peninsula; Terutau Island. [Sanborn, 2014] Thailand, Indochina, India, Malaysia, Vietnam.

##### Notes

Authority: Moulton 1923; Lectotype designated by Beuk (1996).

#### Dundubia laterocurvata

Beuk, 1996

Dundubia laterocurvata Beuk, 1996

##### Materials

- **Type status:** Holotype. **Occurrence:** recordedBy: Cpt. L.C. Kuitert; individualCount: 1; sex: male; **Taxon:** scientificName: Dundubia laterocurvata Beuk, 1996; **Location:** continent: Asia; country: Myanmar; locality: Mogauag; **Event:** eventDate: 16330; **Record Level:** institutionCode: SEMC; basisOfRecord: PreservedSpecimen
- **Type status:** Paratype. **Occurrence:** individualCount: 2; sex: male; **Taxon:** scientificName: Dundubia laterocurvata Beuk, 1996; **Location:** continent: Asia; country: India; locality: Assam; **Record Level:** institutionCode: NHMUK; basisOfRecord: PreservedSpecimen
- **Type status:** Paratype. **Occurrence:** individualCount: 1; sex: male; **Taxon:** scientificName: Dundubia laterocurvata Beuk, 1996; **Location:** continent: Asia; country: India; locality: Assam; **Record Level:** institutionCode: ZMAN; basisOfRecord: PreservedSpecimen
- **Type status:** Paratype. **Occurrence:** recordedBy: Bemet; individualCount: 1; sex: male; **Taxon:** scientificName: Dundubia laterocurvata Beuk, 1996; **Location:** continent: Asia; country: India; locality: Darjiling District; verbatimElevation: 2000 ft; **Record Level:** institutionCode: NHMUK; basisOfRecord: PreservedSpecimen
- **Type status:** Paratype. **Occurrence:** individualCount: 1; sex: male; **Taxon:** scientificName: Dundubia laterocurvata Beuk, 1996; **Location:** continent: Asia; country: India; locality: Dilkoosha, Assam; **Record Level:** institutionCode: NHMUK; basisOfRecord: PreservedSpecimen
- **Type status:** Paratype. **Occurrence:** recordedBy: Foster; individualCount: 1; sex: male; **Taxon:** scientificName: Dundubia laterocurvata Beuk, 1996; **Location:** continent: Asia; country: India; locality: Nazeerah, Assam; **Record Level:** institutionCode: NHMUK; basisOfRecord: PreservedSpecimen
- **Type status:** Paratype. **Occurrence:** individualCount: 1; sex: male; **Taxon:** scientificName: Dundubia laterocurvata Beuk, 1996; **Location:** continent: Asia; country: India; locality: North India; **Record Level:** institutionCode: NHMUK; basisOfRecord: PreservedSpecimen
- **Type status:** Paratype. **Occurrence:** individualCount: 1; sex: male; **Taxon:** scientificName: Dundubia laterocurvata Beuk, 1996; **Location:** continent: Asia; country: India; locality: North Khasia; **Record Level:** institutionCode: ZMAN; basisOfRecord: PreservedSpecimen
- **Type status:** Paratype. **Occurrence:** recordedBy: Knyvett; individualCount: 1; sex: female; **Taxon:** scientificName: Dundubia laterocurvata Beuk, 1996; **Location:** continent: Asia; country: India; locality: Sikkim, E. Himalayas; **Record Level:** institutionCode: NHMUK; basisOfRecord: PreservedSpecimen
- **Type status:** Paratype. **Occurrence:** recordedBy: L.W. Middleton; individualCount: 1; sex: male; **Taxon:** scientificName: Dundubia laterocurvata Beuk, 1996; **Location:** continent: Asia; country: India; locality: Sonapur, Assam; **Record Level:** institutionCode: ZMAN; basisOfRecord: PreservedSpecimen

##### Distribution

[Sanborn, 2014] Northern India, Northern Burma.

##### Notes

Authority: Beuk 1996; An additional 21 male paratypes (9 SEM; 6 ZMA; 6 NHMUK) and 1 female paratype (NHMUK) were designated in the species description, however only the material from India is included here.

#### Dundubia myitkyinensis

Beuk, 1996

Dundubia myitkyinensis Beuk, 1996

##### Materials

- **Type status:** Holotype. **Occurrence:** recordedBy: Grant W. Miller; individualCount: 1; sex: male; **Taxon:** scientificName: Dundubia myitkyinensis Beuk, 1996; **Location:** continent: Asia; country: Myanmar; locality: Myitkyina, Burma; **Event:** eventDate: 20/05/1945; **Record Level:** institutionCode: SEMC; basisOfRecord: PreservedSpecimen

##### Distribution

[Sanborn, 2014] Burma.

##### Notes

Authority: Beuk 1996; An additional 1 female paratype (SEM) was designated in the species description.

#### Dundubia nagarasingna

Distant, 1881

Dundubia nagarasingna Distant, 1881
Cosmopsaltria fratercula Distant, 1912
Dundubia helena Distant, 1912

##### Materials

- **Type status:** Lectotype. **Occurrence:** catalogNumber: BMNH(E) 1009496; individualCount: 1; sex: male; **Taxon:** scientificName: Dundubia nagarasingna Distant, 1881; **Location:** continent: Asia; country: Myanmar; locality: N.W. Burma; **Record Level:** institutionCode: NHMUK; basisOfRecord: PreservedSpecimen
- **Type status:** Paralectotype. **Occurrence:** catalogNumber: BMNH(E) 1009494; individualCount: 1; sex: male; **Taxon:** scientificName: Dundubia nagarasingna Distant, 1881; **Location:** continent: Asia; country: Myanmar; locality: N.W. Burma; **Record Level:** institutionCode: NHMUK; basisOfRecord: PreservedSpecimen

##### Distribution

[Metcalf, 1963] Burma; Northwestern Burma; India; Tenasserim; Cochin China; Indochina; China; Siam; Japan. [Sanborn, 2014] Peninsular Malaysia, Thailand, China, Yunnan, India, Burma, Cambodia, Laos, Vietnam, Indochina, Tenasserim, Conchin China, Siam, Malay Peninsula, Myanmar, .

##### Notes

Authority: Distant 1881; Lectotype designated by Beuk (1996).

#### Dundubia oopaga

(Distant, 1881)

Cosmopsaltria oopaga Distant, 1881
Cosmopsaltria andersoni Distant, 1883

##### Materials

- **Type status:** Lectotype. **Occurrence:** catalogNumber: BMNH(E) 1009518; individualCount: 1; sex: male; **Taxon:** scientificName: Dundubia oopaga (Distant, 1881); **Location:** continent: Asia; country: Myanmar; locality: Burma; **Record Level:** institutionCode: NHMUK; basisOfRecord: PreservedSpecimen

##### Distribution

[Metcalf, 1963] Burma; India; Siam; Indochina. [Duffels and van der Laan, 1985] Thailand; Andaman Islands; Nicobar Islands. [Sanborn, 2014] China, Yunnan, Nanking, India, Burma, Peninsular Malaysia, Borneo, Sarawak, Cambodia, Laos, Thailand, Vietnam, Malay Peninsula, Sumatra, Conchin-China, Jiansu, Indochina, Sabah, Tenasserim, Siam, Indonesia, Myanmar.

##### Notes

Authority: Distant 1881; Lectotype designated by Beuk (1996).

#### Dundubia rufivena rufivena

Walker, 1850

Dundubia rufivena Walker, 1850
Dundubia intemerata Walker, 1857
Fidicina confinis Walker, 1870
Dundubia mellea Distant, 1889

##### Materials

- **Type status:** Syntype. **Occurrence:** individualCount: 2; **Taxon:** scientificName: Dundubia rufivena rufivena Walker, 1850; **Location:** continent: Asia; country: Indonesia; locality: Java; **Record Level:** basisOfRecord: PreservedSpecimen

##### Distribution

[Metcalf, 1963] Java; Malay Peninsula; Sumatra; Nias; Sumbawa; Borneo; Molucca Islands; British North Borneo; Malay States; Amboina; Malaya; North Borneo; Sarawak; Southeastern Borneo; Penang; Sulu Islands; Lombok; Timor; Palawan; Singapore Island; Perak; Johore; Western Borneo; Malacca; Malay Archipelago; Krakatau; Verlaten Island; New Guinea; Sebesi; Mentawei Islands; Siberut; Sipora; Christmas Island; Lang Island. [Duffels and van der Laan, 1985] SE Asia; Malaysia; Sabah; Borneo; Philippines. [Sanborn, 2014] Borneo, Sabah, Sarawak, Java, New Guinea, Krakatau, Verlaten, Sebesi, Nias, Mentawi, Ambiona, Sumatra, Siam, Peninsular Malaysia, Celebes, Singapore, Indonesia, Bantam Island, Sabah, Assam, Philippine Republic, Laos, Irian Jaya, Malacca, India, Myanmar, Malaysia, Elopura, Moluccas, Sumbawa.

##### Notes

Authority: Walker 1850

#### Dundubia terpsichore

(Walker, 1850)

Cephaloxys terpsichore Walker, 1850
Dundubia mannifera var. a Distant, 1892 (nom. nov. pro.)

##### Materials

- **Type status:** Holotype. **Occurrence:** catalogNumber: BMNH(E) 1009531; sex: female; **Taxon:** scientificName: Dundubia terpsichore (Walker, 1850); **Location:** continent: Asia; country: India; locality: E. India; **Record Level:** institutionCode: NHMUK; basisOfRecord: PreservedSpecimen

##### Distribution

[Metcalf, 1963] India; Eastern India; Assam; Perak; Malay Peninsula; Indochina; Malaya; Bengal. [Sanborn, 2014] China, Malaysia, Borneo, Sarawak, Sabah, Myanmar, Tenesserim, Assam, Indo-China, Thailand, Sumatra, Sunda, Asia, Vietnam.

##### Notes

Authority: Walker 1850

#### Dundubia vaginata vaginata

(Fabricius, 1787)

Tettigonia vaginata Fabricius, 1787
Cicada virescens Olivier, 1790
Dundubia immacula Walker, 1850
Dundubia sobria Walker, 1850
Dundubia mannifera Stål, 1866
Dundubia mannifera var. a Distant, 1889

##### Materials

- **Type status:** Holotype. **Taxon:** scientificName: Dundubia vaginata vaginata (Fabricius, 1787); **Location:** continent: Asia; country: Indonesia; locality: Sumatra; **Record Level:** institutionCode: NHMUK; basisOfRecord: PreservedSpecimen

##### Distribution

[Distant, 1889/92] Continental India: Sikkim and Naga Hills; Assam; Seebsagar; North Khasi Hills (1500-3000 ft). Burma: Bhamo; Moulmein. Tenasserim: Myitta-in-the-Valley. [Metcalf, 1963] Sumatra; Java; Assam; Australia; Tenasserim; Hong Kong Island; Borneo; Philippine Island; Morotai; Penang; Sikkim; India; Burma; Nias; Malay Peninsula; Celebes; China; Sinkep Island; Malacca; Palawan; Indochina; Malay States; Australia (?); Malayan Region; Oriental China; Tara bland; Mindanao; Sibuyan Island; Northern Australia; North Borneo; Banguey; Sarawak; Malaya; Malay Archipelago; Johore; Sulu Islands; Molucca Islands; Amboina; Lombok; Sumbawa; Timor; Singapore Island; Perak; Korea; British India; Kwangtang; Eastern China; Mentawai Islands; Pagi Islands; Jainan bland; Bengal; Oriental Region; Siberut. [Sanborn, 2014] China, Malaysia, Australia, Sikkim, Assam, Burma, Tenasserim, Sumatra, Java, Borneo, Celebes, Philippines, Irian Jaya, Sarawak, Sabah, Greater Sunda Islands, Southeast Asia, Peninsular Malaysia, India, Brunei, Hong Kong, Taiwan, Langkawi Island, Vietnam, Greater Sunda Islands, Philippine Republic, Japan, Malay Archipelago, Mindanao, southern China, Southern Myanmar, Panay, Luzon, Sibuyan, Palawan, Yunnan.

##### Notes

Authority: Fabricius 1787

#### Emathia aegrota

Stål, 1866

Emathia aegrota Stål, 1866
Tibicen aurengzebe Distant, 1881

##### Materials

- **Type status:** Holotype. **Occurrence:** sex: male; **Taxon:** scientificName: Emathia aegrota Stål, 1866; **Location:** continent: Asia; country: India; locality: Bombay; **Record Level:** institutionCode: NHRS; basisOfRecord: PreservedSpecimen

##### Distribution

[Distant, 1889/92] India: Bombay; Khandala. [Metcalf, 1963] Bombay; India.

##### Notes

Authority: Stål 1866a

#### Euterpnosia crowfooti

(Distant, 1912)

Terpnosia crowfooti Distant, 1912

##### Materials

- **Type status:** Holotype. **Occurrence:** catalogNumber: BMNH(E) 1009553; recordedBy: A.R. Crowfoot; individualCount: 1; sex: male; **Taxon:** scientificName: Euterpnosia crowfooti (Distant, 1912); **Location:** continent: Asia; country: India; locality: Badamtam, near Darjeeling; **Record Level:** institutionCode: NHMUK; basisOfRecord: PreservedSpecimen

##### Distribution

[Metcalf, 1963] Bengal; Northern India; Indochina; India. [Duffels and van der Laan, 1985] Nepal. [Sanborn, 2014] Vietnam, Nepal, India.

##### Notes

Authority: Distant 1912b

#### Euterpnosia madhava

(Distant, 1881)

Pomponia madhava Distant, 1881

##### Materials

- **Type status:** Holotype. **Occurrence:** catalogNumber: BMNH(E) 1009549; individualCount: 1; sex: male; **Taxon:** scientificName: Euterpnosia madhava (Distant, 1881); **Location:** continent: Asia; country: India; locality: Assam; **Record Level:** institutionCode: NHMUK; basisOfRecord: PreservedSpecimen

##### Distribution

[Metcalf, 1963] Assam; India; Indochina. [Duffels and van der Laan, 1985] Bhutan. [Sanborn, 2014] Vietnam, China, Xizang, Bhutan, India.

##### Notes

Authority: Distant 1881

#### Formotosena montivaga

(Distant, 1889)

Tosena montivaga Distant, 1889

##### Materials

- **Type status:** Syntype. **Occurrence:** individualCount: 1; sex: female; **Taxon:** scientificName: Formotosena montivaga (Distant, 1889); **Location:** continent: Asia; country: India; locality: Naga Hills; **Record Level:** institutionCode: NHMUK; basisOfRecord: PreservedSpecimen
- **Type status:** Syntype. **Occurrence:** catalogNumber: BMNH(E) 1009375; individualCount: 1; sex: male; **Taxon:** scientificName: Formotosena montivaga (Distant, 1889); **Location:** continent: Asia; country: India; locality: Naga Hills; **Record Level:** institutionCode: NHMUK; basisOfRecord: PreservedSpecimen
- **Type status:** Syntype. **Occurrence:** individualCount: 1; sex: male; **Taxon:** scientificName: Formotosena montivaga (Distant, 1889); **Location:** continent: Asia; country: India; locality: Naga Hills; **Record Level:** institutionCode: NHMUK; basisOfRecord: PreservedSpecimen
- **Type status:** Syntype. **Occurrence:** individualCount: 1; sex: male; **Taxon:** scientificName: Formotosena montivaga (Distant, 1889); **Location:** continent: Asia; country: India; locality: Naga Hills; **Record Level:** institutionCode: NHMUK; basisOfRecord: PreservedSpecimen

##### Distribution

[Metcalf, 1963] India; Assam; Fukien. [Sanborn, 2014] Thailand, India, Assam, Fukien.

##### Notes

Authority: Distant 1889a

#### Gaeana atkinsoni

Distant, 1889

Gaeana atkinsoni Distant, 1889

##### Materials

- **Type status:** Holotype. **Occurrence:** catalogNumber: NHMUK 010214245; recordedBy: E.F.T. Atkinson; individualCount: 1; sex: male; **Taxon:** scientificName: Gaeana atkinsoni Distant, 1889; **Location:** continent: Asia; country: India; locality: Karwar, India; **Record Level:** institutionCode: NHMUK; basisOfRecord: PreservedSpecimen

##### Distribution

[Metcalf, 1963] Bombay; India; Travancore; Northern India. [Sanborn, 2014] Western ghats, southern India; India).

##### Notes

Authority: Distant 1889b

#### Gaeana consors

Atkinson, 1884

Gaeana consors Atkinson, 1884
Gaeana maculata var. a Distant, 1892

##### Materials

- **Type status:** Syntype. **Taxon:** scientificName: Gaeana consors Atkinson, 1884; **Location:** continent: Asia; country: India; locality: Naga Hills; **Record Level:** institutionCode: NZSI; basisOfRecord: PreservedSpecimen
- **Type status:** Syntype. **Taxon:** scientificName: Gaeana consors Atkinson, 1884; **Location:** continent: Asia; country: India; locality: Samaguting, Assam; **Record Level:** institutionCode: NZSI; basisOfRecord: PreservedSpecimen

##### Distribution

[Distant, 1889/92] Continental India: Sikkim; Assam; Naga and Khasi Hills; Margherita. Burma: Carin Ghecu. [Metcalf, 1963] Assam; Sikkim; Burma; China; India. [Sanborn, 2014] China, Guangdong, Hunan, Guangxi, Thailand, Guizhou, Sichuan, Yunnan, Burma, Sikkim, India, Burma, Assam, Tonkin.

##### Notes

Authority: Atkinson 1884

#### Gaeana maculata maculata

(Drury, 1773)

Cicada maculata Drury, 1773

##### Materials

- **Type status:** Holotype. **Occurrence:** recordedBy: Dr Fothergill; **Taxon:** scientificName: Gaeana maculata maculata (Drury, 1773); **Location:** continent: Asia; country: China; **Record Level:** basisOfRecord: PreservedSpecimen
- **Type status:** Other material. **Occurrence:** catalogNumber: BMNH(E) 1009596; recordedBy: A.W. Chennell; individualCount: 1; sex: male; **Taxon:** scientificName: Gaeana maculata maculata (Drury, 1773); **Location:** continent: Asia; country: India; locality: Naga Hills; verbatimElevation: 2000 - 6000 ft; **Record Level:** institutionCode: NHMUK; basisOfRecord: PreservedSpecimen

##### Distribution

[Distant, 1889/92] Continental India: Sikkim; Naga Hills; Khasi Hills; Samagooting; Dhansiri Valley; (var consors) Margherita. [Metcalf, 1963] China; Syria; India; Asia; Sikkim; Burma; Hong Kong Island; Australia; South Australia; Tonkin; Assam; Northern Territory (?); Indochina; Kwangtung; Australia (?); Northern Territory; Japan; Kwangsi; Hainan Island; Fukien; Bengal. [Sanborn, 2014] China, Kwangsi, Sikhim, Assam, Naga Hills, Khasi Hills, Margherita, Samagooting Valley, Dhansiri Valley, Burma, Karenne, Tonkin, Hong Kong, Myanmar, India, Vietnam, Guangxi, Jiangxi, Fujian, Guangdong, Hainan, Guizhou, Sichuan, Japan, Indonesia, Malaysia, Burma, Bangladesh, Australia, Southeast Asia, Sri Lanka, North Vietnam, South China.

##### Notes

Authority: Drury 1773

#### Graptotettix guttatus

Stål, 1866

Graptotettix guttatus Stål, 1866

##### Materials

- **Type status:** Holotype. **Occurrence:** sex: female; **Taxon:** scientificName: Graptotettix guttatus Stål, 1866; **Location:** continent: Asia; country: India; locality: Himalaya; **Record Level:** institutionCode: NHRS; basisOfRecord: PreservedSpecimen
- **Type status:** Other material. **Occurrence:** catalogNumber: BMNH(E) 1009585; recordedBy: E.F.T. Atkinson; individualCount: 1; sex: male; **Taxon:** scientificName: Graptotettix guttatus Stål, 1866; **Location:** locality: Locality unknown; **Record Level:** institutionCode: NHMUK; basisOfRecord: PreservedSpecimen

##### Distribution

[Metcalf, 1963] India; Himalayas; Sikkim; China; Assam. [Duffels and van der Laan, 1985] Nepal. [Sanborn, 2014] China.

##### Notes

Authority: Stål 1866a

#### Graptotettix thoracicus

Distant, 1892

Graptotettix thoracicus Distant, 1892

##### Materials

- **Type status:** Holotype. **Occurrence:** catalogNumber: BMNH(E) 1009586; recordedBy: William Doherty; individualCount: 1; sex: male; **Taxon:** scientificName: Graptotettix thoracicus Distant, 1892; **Location:** continent: Asia; country: Myanmar; locality: Momeit [Mong Mit]; **Record Level:** institutionCode: NHMUK; basisOfRecord: PreservedSpecimen

##### Distribution

[Metcalf, 1963] Burma.

##### Notes

Authority: Distant 1892b

#### Gudaba maculata

Distant, 1912

Gudaba maculata Distant, 1912

##### Materials

- **Type status:** Syntype. **Occurrence:** catalogNumber: BMNH(E) 1009561; recordedBy: C.T. Bingham; individualCount: 1; sex: male; **Taxon:** scientificName: Gudaba maculata Distant, 1912; **Location:** continent: Asia; country: India; locality: Sikkim; **Event:** eventDate: ??/??/1907; **Record Level:** institutionCode: NHMUK; basisOfRecord: PreservedSpecimen
- **Type status:** Syntype. **Occurrence:** catalogNumber: BMNH(E) 1009617; recordedBy: N.C. Chatterji; individualCount: 1; sex: female; **Taxon:** scientificName: Gudaba maculata Distant, 1912; **Location:** continent: Asia; country: India; locality: Dehra Dun; **Event:** eventDate: 6/07/1912; **Record Level:** institutionCode: NHMUK; basisOfRecord: PreservedSpecimen

##### Distribution

[Metcalf, 1963] Sikkim; United Provinces; Northern India. [Sanborn, 2014] China, Yunnan, Hainan, Thailand, Sikkim, India.

##### Notes

Authority: Distant 1912b

#### Gudaba marginatus

(Distant, 1897)

Calcagninus marginatus Distant, 1897

##### Materials

- **Type status:** Holotype. **Occurrence:** catalogNumber: BMNH(E) 1009560; recordedBy: Watson; individualCount: 1; sex: male; **Taxon:** scientificName: Gudaba marginatus (Distant, 1897); **Location:** continent: Asia; country: Myanmar; locality: North Chin Hills; **Record Level:** institutionCode: NHMUK; basisOfRecord: PreservedSpecimen

##### Distribution

[Metcalf, 1963] Burma. [Sanborn, 2014] China, Burma.

##### Notes

Authority: Distant 1897; Metcalf (1963) listed India in reference to Mathur (1953) in which Burma was considerd a part of "British India". The type locality (Chin Hills) extends into India (Lushai Hills - Nagaland), thus this species may be recorded in Nagaland in future.

#### Haphsa bindusara

(Distant, 1881)

Pomponia bindusara Distant, 1881

##### Materials

- **Type status:** Holotype. **Occurrence:** catalogNumber: BMNH(E) 1009459; individualCount: 1; sex: male; **Taxon:** scientificName: Haphsa bindusara (Distant, 1881); **Location:** continent: Asia; country: Myanmar; locality: Upper Tenasserim; **Record Level:** institutionCode: NHMUK; basisOfRecord: PreservedSpecimen

##### Distribution

[Distant, 1906] Burma: Teinzo; Karen Hills. Upper Tenasserim. [Metcalf, 1963] Tenasserim; India; Burma; Laos; Indochina. [Sanborn, 2014] Pakistan (?), Burma, India, Yunnan, China, Laos, Thailand, Tenasserim, Indochina, North Vietnam, Myanmar, Vietnam, Bangladesh (?).

##### Notes

Authority: Distant 1881; Metcalf (1963) stated "India" in reference to Atkinson (1886) who was referring to the type specimen from Upper Tenasserim (Myanmar). Metcalf (1963) also listed India in reference to Mathur (1953) who stated "India.-Burma: Katha, Mohnyin Res." as a part of then "British India". Listings of India since then are likely to be in reference to published localities, most likely that from Metcalf (1963). In addition, Sanborn (2014) listed Pakistan and Bangladesh in reference to a specimen collected in Chittagong (former "East Pakistan" now Bangladesh), however the identification of the specimen from Chaudhry et al. (1970) (Aola sp.? bindusara) has not been confirmed thus the Bangladesh distribution is not conclusive at this point.

#### Haphsa durga

(Distant, 1881)

Cosmopsaltria durga Distant, 1881

##### Materials

- **Type status:** Holotype. **Occurrence:** catalogNumber: BMNH(E) 1009451; recordedBy: A.W. Chennell; individualCount: 1; sex: male; **Taxon:** scientificName: Haphsa durga (Distant, 1881); **Location:** continent: Asia; country: India; locality: N. Khasi Hills, British Assam; verbatimElevation: 1500 - 3000 ft; **Record Level:** institutionCode: NHMUK; basisOfRecord: PreservedSpecimen

##### Distribution

[Distant, 1889/92] India: Assam, North Khasi Hills (1500-3000 ft); Naga Hills. [Metcalf, 1963] Assam; India; Laos. [Sanborn, 2014] China, Yunnan, India, Laos, Thailand, Vietnam, Assam, Guangdong, Fujian.

##### Notes

Authority: Distant 1881

#### Haphsa karenensis

Ollenbach, 1929

Haphsa karenensis Ollenbach, 1929
Meimuna nauhkae Boulard, 2005

##### Materials

- **Type status:** Holotype. **Occurrence:** recordedBy: W.S. Wood; individualCount: 1; sex: male; **Taxon:** scientificName: Haphsa karenensis Ollenbach, 1929; **Location:** continent: Asia; country: Myanmar; locality: Karen Hills, Thandaung; **Record Level:** institutionCode: IFRI; basisOfRecord: PreservedSpecimen

##### Distribution

[Metcalf, 1963] Burma; India. [Sanborn, 2014] Thailand.

##### Notes

Authority: Ollenbach 1929; Metcalf (1963) listed India in reference Burma being part of "British India". The type locality (Thandaung) is within modern day Myanmar and the species has not yet been recorded from modern day India.

#### Haphsa nicomache

(Walker, 1850)

Dundubia nicomache Walker, 1850
Cicada delineata Walker, 1858

##### Materials

- **Type status:** Holotype. **Occurrence:** catalogNumber: BMNH(E) 1009463; sex: male; **Taxon:** scientificName: Haphsa nicomache (Walker, 1850); **Location:** continent: Asia; country: India; locality: N. India; **Record Level:** institutionCode: NHMUK; basisOfRecord: PreservedSpecimen

##### Distribution

[Distant, 1889/92] Continental India: North India; Sikkim; Margherita (Upper Assam); Naga Hills. [Metcalf, 1963] India; Hindustan; Northern India; Sikkim; Mysore; Assam; Bengal; Northern Bengal; Northern United Provinces; Uttar Pradesh. [Duffels and van der Laan, 1985] Nepal. [Sanborn, 2014] India, North India, Pakistan.

##### Notes

Authority: Walker 1850

#### Haphsa scitula

(Distant, 1888)

Pomponia scitula Distant, 1888

##### Materials

- **Type status:** Syntype. **Occurrence:** recordedBy: Leonardo Fea; sex: male; **Taxon:** scientificName: Haphsa scitula (Distant, 1888); **Location:** continent: Asia; country: Myanmar; locality: Meetan, Tenasserim; **Record Level:** institutionCode: MSNG; basisOfRecord: PreservedSpecimen
- **Type status:** Syntype. **Occurrence:** catalogNumber: BMNH(E) 1009407; sex: male; **Taxon:** scientificName: Haphsa scitula (Distant, 1888); **Location:** continent: Asia; country: Myanmar; locality: Teinzo, Burma; **Event:** eventDate: ??/??/1886; **Record Level:** institutionCode: NHMUK; basisOfRecord: PreservedSpecimen

##### Distribution

[Distant, 1889/92] Continental India: Margherita (Upper Assam). Burma: Teinzo. Tenasserim: Meetan. [Metcalf, 1963] Burma; Tenasserim; India; Tonkin; Assam; Indochina; Cambodia; Yunnan; Madras; China. [Sanborn, 2014] China, Yunnan, Burma, Annam, Cambodia, Pakistan, India, Xinjiang, Guangxi, Thailand, Tonkin, Asia, Vietnam, Indochina, Myanmar, Bangladesh.

##### Notes

Authority: Distant 1888d

#### Haphsa stellata

Lee, 2009

Haphsa stellata Lee, 2009

##### Materials

- **Type status:** Holotype. **Occurrence:** recordedBy: Y. J. Lee; individualCount: 1; sex: male; **Taxon:** scientificName: Haphsa stellata Lee, 2009; **Location:** continent: Asia; country: India; locality: Yercaud, Shevaroy Hills, South India; verbatimElevation: 4500 ft; **Event:** eventDate: ??/06/1997; **Record Level:** basisOfRecord: PreservedSpecimen
- **Type status:** Paratype. **Occurrence:** recordedBy: Y. J. Lee; individualCount: 1; sex: male; **Taxon:** scientificName: Haphsa stellata Lee, 2009; **Location:** continent: Asia; country: India; locality: Yercaud, Shevaroy Hills, South India; verbatimElevation: 4500 ft; **Event:** eventDate: ??/05/1999; **Record Level:** basisOfRecord: PreservedSpecimen

##### Distribution

[Lee, 2009] India. [Sanborn, 2014] India.

##### Notes

Authority: Lee 2009b

#### Huechys beata

Chou et al., 1997

Huechys beata Chou et al., 1997
Huechys sanguinea var. b Distant, 1892

##### Materials

- **Type status:** Syntype. **Occurrence:** recordedBy: A.W. Chennell; **Taxon:** scientificName: Huechys beata Chou et al., 1997; **Location:** continent: Asia; country: India; locality: Assam; **Record Level:** basisOfRecord: PreservedSpecimen
- **Type status:** Syntype. **Taxon:** scientificName: Huechys beata Chou et al., 1997; **Location:** continent: Asia; country: Indonesia; locality: Sumatra; **Record Level:** basisOfRecord: PreservedSpecimen

##### Distribution

[Metcalf, 1963] Assam; Sumatra; Malay Archipelago; India; Tonkin; Kwangsi; Fukien; China. [Sanborn, 2014] China, Guangxi, Yunnan, India, Malaysia, Thailand, Vietnam, Thailand, Indonesia, Assam, Sumatra, South China.

##### Notes

Authority: Chou et al. 1997; Originally Huechys sanguinea var. b Distant, 1892;

#### Huechys haematica

Distant, 1888

Huechys haematica Distant, 1888

##### Materials

- **Type status:** Holotype. **Taxon:** scientificName: Huechys haematica Distant, 1888; **Location:** continent: Asia; country: Myanmar; locality: Mt. Mooleyit, Tenasserim; verbatimElevation: 600 - 1200 m; **Record Level:** basisOfRecord: PreservedSpecimen

##### Distribution

[Distant, 1906] India; Tenasserim; Philippines. [Haupt 1924] Tenasserim (Mt. Mooleyit); Calcutta. [Metcalf, 1963] Tenasserim; India; Philippine Islands; Bengal; Kwangsi; China. [Sanborn, 2014] China, Guangxi, India, Philippines.

##### Notes

Authority: Distant 1888a

#### Huechys incarnata testacea

(Fabricius, 1787)

Tettigonia testacea Fabricius, 1787

##### Materials

- **Type status:** Holotype. **Taxon:** scientificName: Huechys incarnata testacea (Fabricius, 1787); **Location:** continent: Asia; country: India; locality: Tranquebariae (Tharangambadi); **Record Level:** basisOfRecord: PreservedSpecimen

##### Distribution

[Metcalf, 1963] Tranquebar; Madras; India; China; Asia; Bengal; Sumatra.

##### Notes

Authority: Fabricius 1787

#### Huechys sanguinea philaemata

(Fabricius, 1803)

Tettigonia philaemata Fabricius, 1803
Huechys sanguinea var. a Distant, 1892

##### Materials

- **Type status:** Holotype. **Occurrence:** recordedBy: Dru Drury; **Taxon:** scientificName: Huechys sanguinea philaemata (Fabricius, 1803); **Location:** continent: Asia; country: China; **Record Level:** basisOfRecord: PreservedSpecimen

##### Distribution

[Metcalf, 1963] China; India; Java; Assam; Philippine Islands; Bengal; Tenasserim; Northern Bengal; Silhet; Northern India; Burma; Hong Kong Island; Japan; Formosa; Borneo; Kiangsu; Chekiang; Anhwei; Shantung; Kiangsu (?); Eastern China; Kwangsi; Asia. [Duffels and van der Laan, 1985] Taiwan; China. [Sanborn, 2014] China, Haichos, Nanking, Soochow, Hangchow, Burma, Formosa, Anhui, Jiangsu, Jiangxi, Zhejiang, Taiwan, Hainan, Guangxi, Yunnan, India, Vietnam.

##### Notes

Authority: Fabricius 1803

#### Huechys sanguinea sanguinea

(De Geer, 1773)

Cicada sanguinea De Geer, 1773
Tettigonia sanguinolenta Fabricius, 1775
Huechys vesicatoria Smith, 1871
Huechys quadrispinosa Haupt, 1924
Huechys sanguinea philaemata albifascia Kato, 1927

##### Materials

- **Type status:** Holotype. **Taxon:** scientificName: Huechys sanguinea sanguinea (De Geer, 1773); **Location:** continent: Asia; country: China; **Record Level:** institutionCode: NHRS; basisOfRecord: PreservedSpecimen
- **Type status:** Other material. **Occurrence:** catalogNumber: BMNH(E) 1009587; recordedBy: Levick; individualCount: 1; sex: female; **Taxon:** scientificName: Huechys sanguinea sanguinea (De Geer, 1773); **Location:** continent: Asia; country: Indonesia; locality: Sumatra; **Record Level:** institutionCode: NHMUK; basisOfRecord: PreservedSpecimen

##### Distribution

[Distant, 1889/92] India: Sikkim; Assam; Calcutta. Burma: Kakhien Hills (Rangoon); Thagata (Tenasserim). [Metcalf, 1963] China; France; Sumatra; America; India; Bengal; Hong Kong Island; Asia; Java; Malacca; Timor; Tenasserim; Singapore Island; Sikkim; Calcutta; Japan; Sibsagar; Tonkin; Burma; Nias; Molucca Islands; Sumbawa; Borneo; Laos; Lombok; Assam; Malay Peninsula; Malay States; Macao; Hupeh; Formosa; Palawan; Tanimbar Islands; Philippine Islands; Sulu Island; Amboina; Perak; Johore; Penang; Oriental Region; Cochin China; Sunda Islands; Indochina; Malay Archipelago; Malaya; Sarawak; Fukien; Kwangtung; Southern Asia; Eastern India; Madras; Siam; Celebes; Southern China; Telo Island; Batu Islands; Kiangsu; Kwangsi; Lower Burma; Chekiang; Indonesia; Hainan. [Duffels and van der Laan, 1985] Taiwan; Andaman Islands; Nicobar Islands; Japan; China; Philippines. [Sanborn, 2014] China, Soochow, Kwangsi, Canton, Macao, Han-lik, Hangchow, Sikhim, Assam, Brahmaputra, Calcutta, Burma, Rangoon, Kakhein Hills, Tenasserim, Thagata, Myitta, Malay Peninsula, Sumatra, Borneo, Timor Laut, Tonkin, Than-Moi, Formosa, Pakistan, India, Hong Kong, Siam, Singapore, Hunan, Shaanxi, Henan, Hubei, Jiangsu, Zhejiang, Jiangxi, Fujian, Taiwan, Guangdong, Hainan, Guangxi, Sichuan, Yunnan, Indonesia, Kalimantan, Sikkim, Maymer, Andaman Island, Nicobar Island, Southeast Asia, Timor, South China, Palawan, Leyte.

##### Notes

Authority: De Geer 1773

#### Huechys thoracica

Distant, 1879

Huechys thoracica Distant, 1879

##### Materials

- **Type status:** Holotype. **Occurrence:** sex: female; **Taxon:** scientificName: Huechys thoracica Distant, 1879; **Location:** continent: Asia; country: Myanmar; locality: Upper Tenasserim; **Record Level:** basisOfRecord: PreservedSpecimen

##### Distribution

[Distant, 1889/92] Burma: Karen Hills; Ruby Mines; Myitta (Tenasserim). [Metcalf, 1963] Tenasserim; Hindustan; Burma; Upper Burma; Assam; Malaya Peninsula; Silhet; India. [Duffels and van der Laan, 1985] India. [Sanborn, 2014] China, Yunnan, Burma, Malaysia, Southheast Asia, Vietnam.

##### Notes

Authority: Distant 1879

#### Hyalessa expansa

(Walker, 1858)

Carineta expansa Walker, 1858

##### Materials

- **Type status:** Holotype. **Occurrence:** sex: male; **Taxon:** scientificName: Hyalessa expansa (Walker, 1858); **Location:** continent: Asia; country: India; locality: Hindostan; **Record Level:** basisOfRecord: PreservedSpecimen
- **Type status:** Other material. **Occurrence:** catalogNumber: BMNH(E) 1009465; individualCount: 1; sex: male; **Taxon:** scientificName: Hyalessa expansa (Walker, 1858); **Location:** continent: Asia; country: India; locality: Himalaya (eastern); **Record Level:** institutionCode: NHMUK; basisOfRecord: PreservedSpecimen

##### Distribution

[Distant, 1889/92] Continental India: Eastern Himalaya Mts; Sikkim. [Metcalf, 1963] Hindustan; India; China; Sikkim; Eastern Himalayas; Northern Bengal; Assam. [Duffels and van der Laan, 1985] Nepal.

##### Notes

Authority: Walker 1858a

#### Hyalessa mahoni

(Distant, 1906)

Oncotympana mahoni Distant, 1906

##### Materials

- **Type status:** Holotype. **Occurrence:** catalogNumber: BMNH(E) 1009466; recordedBy: Mahoni; individualCount: 1; sex: male; **Taxon:** scientificName: Hyalessa mahoni (Distant, 1906); **Location:** continent: Asia; country: India; locality: Mussooree; verbatimElevation: 5000 ft; **Event:** eventDate: 29/07/1905; **Record Level:** institutionCode: NHMUK; basisOfRecord: PreservedSpecimen

##### Distribution

[Metcalf, 1963] Mysore; India; Northwestern India; Uttar Pradesh.

##### Notes

Authority: Distant 1906d

#### Hyalessa melanoptera

(Distant, 1904)

Pomponia melanoptera Distant, 1904

##### Materials

- **Type status:** Syntype. **Occurrence:** catalogNumber: BMNH(E) 1009469; recordedBy: P.W. Mackinnon; sex: male; **Taxon:** scientificName: Hyalessa melanoptera (Distant, 1904); **Location:** continent: Asia; country: India; locality: Mussoorie; verbatimElevation: 4000 ft; **Event:** eventDate: ??/09/1903; **Record Level:** institutionCode: NHMUK; basisOfRecord: PreservedSpecimen
- **Type status:** Syntype. **Occurrence:** recordedBy: P.W. Mackinnon; sex: female; **Taxon:** scientificName: Hyalessa melanoptera (Distant, 1904); **Location:** continent: Asia; country: India; locality: Mussoorie; verbatimElevation: 4000 ft; **Event:** eventDate: ??/09/1903; **Record Level:** basisOfRecord: PreservedSpecimen

##### Distribution

[Metcalf, 1963] Mysore; India; Uttar Pradesh.

##### Notes

Authority: Distant 1904b

#### Hyalessa obnubila

(Distant, 1888)

Pomponia obnubila Distant, 1888

##### Materials

- **Type status:** Holotype. **Taxon:** scientificName: Hyalessa obnubila (Distant, 1888); **Location:** continent: Asia; country: India; locality: Simla; **Record Level:** institutionCode: NZSI; basisOfRecord: PreservedSpecimen
- **Type status:** Other material. **Occurrence:** catalogNumber: BMNH(E) 1009467; individualCount: 1; sex: male; **Taxon:** scientificName: Hyalessa obnubila (Distant, 1888); **Location:** continent: Asia; country: India; locality: Dharamsala, W. Himalaya; **Event:** eventDate: ??/??/1919; **Record Level:** institutionCode: NHMUK; basisOfRecord: PreservedSpecimen

##### Distribution

[Distant, 1906] India: Simla; Tehri-Garhwal. [Metcalf, 1963] Punjab; India; Uttar Pradesh. [Duffels and van der Laan, 1985] Bhutan; Nepal. [Sanborn, 2014] Pakistan, India, Bhutan, Nepal.

##### Notes

Authority: Distant 1888c; Distant (1906c) states that the type specimen is male.

#### Hyalessa stratoria

(Distant, 1905)

Oncotympana stratoria Distant, 1905

##### Materials

- **Type status:** Holotype. **Occurrence:** sex: male; **Taxon:** scientificName: Hyalessa stratoria (Distant, 1905); **Location:** continent: Asia; country: China; locality: Yunnan; **Record Level:** institutionCode: MNHN; basisOfRecord: PreservedSpecimen

##### Distribution

[Metcalf, 1963] Yunnan; China. [Duffels and van der Laan, 1985] Nepal; China. [Sanborn, 2014] China, Yunnan, Nepal.

##### Notes

Authority: Distant 1905e

#### Karenia ravida

Distant, 1888

Karenia ravida Distant, 1888

##### Materials

- **Type status:** Holotype. **Occurrence:** catalogNumber: BMNH(E) 1009612; recordedBy: Leonardo Fea; individualCount: 1; sex: male; **Taxon:** scientificName: Karenia ravida Distant, 1888; **Location:** continent: Asia; country: Myanmar; locality: Catcin Cauri, Kakhien Hills, Burma; **Event:** eventDate: ??/11/1886; **Record Level:** institutionCode: NHMUK; basisOfRecord: PreservedSpecimen

##### Distribution

[Metcalf, 1963] Burma. [Sanborn, 2014] China, Yunnan, Burma, Thailand.

##### Notes

Authority: Distant 1888a

#### Khimbya cuneata

(Distant, 1897)

Pomponia cuneata Distant, 1897

##### Materials

- **Type status:** Holotype. **Occurrence:** recordedBy: Watson; sex: male; **Taxon:** scientificName: Khimbya cuneata (Distant, 1897); **Location:** continent: Asia; country: Myanmar; locality: North Chin Hills; **Record Level:** basisOfRecord: PreservedSpecimen

##### Distribution

[Metcalf, 1963] Burma.

##### Notes

Authority: Distant 1897; Metcalf (1963) listed India in reference to Mathur (1953) in which Burma was considerd a part of "British India". The type locality (Chin Hills) extends into India (Lushai Hills - Nagaland), thus this species may be recorded in Nagaland in future.

#### Khimbya diminuta

(Walker, 1850)

Dundubia diminuta Walker, 1850

##### Materials

- **Type status:** Holotype. **Occurrence:** catalogNumber: BMNH(E) 1009452; individualCount: 1; sex: male; **Taxon:** scientificName: Khimbya diminuta (Walker, 1850); **Location:** locality: Locality unknown; **Record Level:** institutionCode: NHMUK; basisOfRecord: PreservedSpecimen

##### Distribution

[Distant, 1906] India: Bombay; Karwar. Burma: Thaungyin (Tenasserim). [Metcalf, 1963] India; Bombay; Tenasserim.

##### Notes

Authority: Walker 1850

#### Khimbya evanescens

(Walker, 1858)

Dundubia evanescens Walker, 1858

##### Materials

- **Type status:** Syntype. **Occurrence:** catalogNumber: BMNH(E) 1009455; sex: male; **Taxon:** scientificName: Khimbya evanescens (Walker, 1858); **Location:** continent: Asia; country: India; locality: Hindostan; **Record Level:** institutionCode: NHMUK; basisOfRecord: PreservedSpecimen

##### Distribution

[Metcalf, 1963] Hindustan; India; Burma; Uttar Pradesh. [Sanborn, 2014] Hindustan.

##### Notes

Authority: Walker 1858a; Multiple male specimens used in species description;

#### Khimbya immsi

Distant, 1912

Khimbya immsi Distant, 1912

##### Materials

- **Type status:** Lectotype. **Occurrence:** catalogNumber: BMNH(E) 1009456; individualCount: 1; sex: male; **Taxon:** scientificName: Khimbya immsi Distant, 1912; **Location:** continent: Asia; country: India; locality: Goalpara, Assam; **Record Level:** institutionCode: NHMUK; basisOfRecord: PreservedSpecimen

##### Distribution

[Metcalf, 1963] Assam; India.

##### Notes

Authority: Distant 1912c

#### Khimbya sita

(Distant, 1881)

Cosmopsaltria sita Distant, 1881

##### Materials

- **Type status:** Holotype. **Occurrence:** catalogNumber: BMNH(E) 1009453; individualCount: 1; sex: male; **Taxon:** scientificName: Khimbya sita (Distant, 1881); **Location:** continent: Asia; country: India; locality: Bombay Province; **Record Level:** institutionCode: NHMUK; basisOfRecord: PreservedSpecimen

##### Distribution

[Distant, 1889/92] India: Bombay Province; Karwar. [Metcalf, 1963] Southern India; Bombay (?); India; Tonkin. [Sanborn, 2014] China, Guangxi, india, Vietnam.

##### Notes

Authority: Distant 1881

#### Kumanga sandaracata

(Distant, 1888)

Baeturia sandaracata Distant, 1888

##### Materials

- **Type status:** Holotype. **Occurrence:** recordedBy: Leonardo Fea; individualCount: 1; sex: male; **Taxon:** scientificName: Kumanga sandaracata (Distant, 1888); **Location:** continent: Asia; country: Myanmar; locality: Teinzo, Burma; **Record Level:** institutionCode: MSNG; basisOfRecord: PreservedSpecimen

##### Distribution

[Metcalf, 1963] Burma.

##### Notes

Authority: Distant 1888d

#### Lahugada dohertyi

(Distant, 1891)

Pomponia dohertyi Distant, 1891

##### Materials

- **Type status:** Holotype. **Occurrence:** catalogNumber: BMNH(E) 1009462; recordedBy: W.M. Doherty; individualCount: 1; sex: male; **Taxon:** scientificName: Lahugada dohertyi (Distant, 1891); **Location:** continent: Asia; country: India; locality: Margherita, Upper Assam; **Record Level:** institutionCode: NHMUK; basisOfRecord: PreservedSpecimen

##### Distribution

[Metcalf, 1963] India; Assam. [Sanborn, 2014] India: Margherita, Manas Tiger Reserve, Assam; Cooch Behar, N. Paschimbanga.

##### Notes

Authority: Distant 1891

#### Lemuriana apicalis

(Germar, 1830)

Cicada apicalis Germar, 1830
Cicada semicinta Walker, 1850

##### Materials

- **Type status:** Holotype. **Occurrence:** recordedBy: Westermann; **Taxon:** scientificName: Lemuriana apicalis (Germar, 1830); **Location:** continent: Asia; country: India; locality: Bengalia; **Record Level:** basisOfRecord: PreservedSpecimen

##### Distribution

[Distant, 1906] India: Bombay; Karwar; Mussooree. [Metcalf, 1963] Bengal; India; Northern India; Mysore; Bombay; Indochina; Madras; United Provinces; Central Provinces; Uttar Pradesh. [Duffels and van der Laan, 1985] Nepal. [Sanborn, 2014] Vietnam, Nepal, India, Oriental Region.

##### Notes

Authority: Germar 1830

#### Leptopsaltria andamanensis

Distant, 1888

Leptopsaltria andamanensis Distant, 1888

##### Materials

- **Type status:** Holotype. **Occurrence:** catalogNumber: BMNH(E) 1009437; recordedBy: J. Wood-Mason; individualCount: 1; sex: male; **Taxon:** scientificName: Leptopsaltria andamanensis Distant, 1888; **Location:** continent: Asia; country: India; locality: Andaman Islands; **Record Level:** institutionCode: NHMUK; basisOfRecord: PreservedSpecimen

##### Distribution

[Metcalf, 1963] Andaman Islands. [Sanborn, 2014] Andaman Islands.

##### Notes

Authority: Distant 1888b

#### Leptopsaltria samia

(Walker, 1850)

Dundubia samia Walker, 1850

##### Materials

- **Type status:** Holotype. **Occurrence:** catalogNumber: BMNH(E) 1009438; individualCount: 1; sex: male; **Taxon:** scientificName: Leptopsaltria samia (Walker, 1850); **Location:** continent: Asia; country: India; locality: N. India; **Record Level:** institutionCode: NHMUK; basisOfRecord: PreservedSpecimen

##### Distribution

[Distant, 1889/92] Continental India: N. India; Sikkim. [Metcalf, 1963] India; Northern India; Tonkin; Sikkim; Assam. [Sanborn, 2014] China, Guangxi, Yunnan, India, Sikkim, Vietnam, Tonkin, Thailand, North Vietnam.

##### Notes

Authority: Walker 1850

#### Leptopsaltria tuberosa

(Signoret, 1847)

Cicada tuberosa Signoret, 1847

##### Materials

- **Type status:** Syntype. **Taxon:** scientificName: Leptopsaltria tuberosa (Signoret, 1847); **Location:** continent: Asia; country: Indonesia; locality: Java; **Record Level:** basisOfRecord: PreservedSpecimen
- **Type status:** Syntype. **Taxon:** scientificName: Leptopsaltria tuberosa (Signoret, 1847); **Location:** continent: Asia; country: Malaysia; locality: Malacca; **Record Level:** basisOfRecord: PreservedSpecimen

##### Distribution

[Distant, 1906; Distant, 1889/92] Continental India: Sikkim; Assam (Khasi Hills). [Metcalf, 1963] Java; Malacca; India; Japan; Sikkim; Assam; Daito Islands; Kyushu; Honshu; Ryukyu Islands; Formosa; Oriental Region; Japan (?). [Duffels and van der Laan, 1985] Taiwan; Java; India; Nepal. [Sanborn, 2014] Nepal, India.

##### Notes

Authority: Signoret 1847a

#### Lethama locusta

(Walker, 1850)

Cephaloxys locusta Walker, 1850

##### Materials

- **Type status:** Holotype. **Occurrence:** catalogNumber: BMNH(E) 1009519; individualCount: 1; sex: female; **Taxon:** scientificName: Lethama locusta (Walker, 1850); **Location:** continent: Asia; country: India; locality: E. India; **Record Level:** institutionCode: NHMUK; basisOfRecord: PreservedSpecimen
- **Type status:** Other material. **Occurrence:** catalogNumber: BMNH(E) 1009520; individualCount: 1; sex: male; **Taxon:** scientificName: Lethama locusta (Walker, 1850); **Location:** continent: Asia; country: India; locality: E. India; **Record Level:** institutionCode: NHMUK; basisOfRecord: PreservedSpecimen

##### Distribution

[Metcalf, 1963] India; Eastern India; Bombay. [Sanborn, 2014] India.

##### Notes

Authority: Walker 1850

#### Linguacicada continuata

(Distant, 1888)

Cicadetta continuata Distant, 1888

##### Materials

- **Type status:** Syntype. **Occurrence:** catalogNumber: BMNH(E) 1009613; sex: female; **Taxon:** scientificName: Linguacicada continuata (Distant, 1888); **Location:** continent: Asia; country: Pakistan; locality: Quetta, Baluchistan; **Record Level:** institutionCode: NHMUK; basisOfRecord: PreservedSpecimen

##### Distribution

[Metcalf, 1963] Baluchistan; India; Quetta; Pakistan; Northwestern India. [Sanborn, 2014] China, Xinjiang, Pakistan, India.

##### Notes

Authority: Distant 1888b; Multiple specimens (number unknown) used in species description.

#### Lycurgus subvittus

(Walker, 1850)

Cicada subvittus Walker, 1850
Cicada strigosa Walker, 1858

##### Materials

- **Type status:** Holotype. **Occurrence:** catalogNumber: BMNH(E) 1009611; sex: male; **Taxon:** scientificName: Lycurgus subvittus (Walker, 1850); **Location:** continent: Asia; country: India; locality: N. India; **Record Level:** institutionCode: NHMUK; basisOfRecord: PreservedSpecimen

##### Distribution

[Distant, 1889/92] India: Sikkim; N.W. Himalaya. [Metcalf, 1963] India; Hindustan; Northern India; Mysore; Sikkim; United Provinces; Northwestern Himalayas; China; Uttar Pradesh. [Duffels and van der Laan, 1985] China; India. [Sanborn, 2014] China, Mussooree, Himalya, Sikhim, India.

##### Notes

Authority: Walker 1850

#### Macrosemia assamensis

(Distant, 1905)

Platylomia assamensis Distant, 1905

##### Materials

- **Type status:** Holotype. **Occurrence:** catalogNumber: BMNH(E) 1009516; recordedBy: E.F.T. Atkinson; individualCount: 1; sex: male; **Taxon:** scientificName: Macrosemia assamensis (Distant, 1905); **Location:** continent: Asia; country: India; locality: Assam; **Record Level:** institutionCode: NHMUK; basisOfRecord: PreservedSpecimen

##### Distribution

[Metcalf, 1963] Assam; India; Uttar Pradesh. [Sanborn, 2014] China, Yunnan, India, Thailand, Vietnam.

##### Notes

Authority: Distant 1905a

#### Macrosemia beaudouini

(Boulard, 2003)

Orientopsaltria beaudouini Boulard, 2003

##### Materials

- **Type status:** Holotype. **Occurrence:** recordedBy: M. Boulard, S. Sulaiya, K Chueata; individualCount: 1; sex: male; **Taxon:** scientificName: Macrosemia beaudouini (Boulard, 2003); **Location:** continent: Asia; country: Thailand; locality: Houaynamgun, Chiang Mai; **Event:** eventDate: 2-15/09/2002; **Record Level:** institutionCode: MNHN; basisOfRecord: PreservedSpecimen

##### Distribution

[Sanborn, 2014] Thailand, Myanmar, Laos.

##### Notes

Authority: Boulard 2003; An additional 6 male paratypes (MNHN) were designated in the species description.

#### Macrosemia saturata saturata

(Walker, 1858)

Dundubia saturata Walker, 1858
Dundubia obtecta Walker, 1850

##### Materials

- **Type status:** Syntype. **Occurrence:** sex: male; **Taxon:** scientificName: Macrosemia saturata (Walker, 1858); **Location:** continent: Asia; country: India; locality: Sikkim, Himalaya, North India; **Record Level:** institutionCode: NHMUK; basisOfRecord: PreservedSpecimen
- **Type status:** Other material. **Occurrence:** catalogNumber: BMNH(E) 1009470; individualCount: 1; sex: male; **Taxon:** scientificName: Macrosemia saturata (Walker, 1858); **Location:** continent: Asia; country: India; locality: Himalaya; **Record Level:** institutionCode: NHMUK; basisOfRecord: PreservedSpecimen
- **Type status:** Syntype. **Occurrence:** sex: female; **Taxon:** scientificName: Macrosemia saturata (Walker, 1858); **Location:** continent: Asia; country: India; locality: Sikkim, Himalaya, North India; **Record Level:** institutionCode: NHMUK; basisOfRecord: PreservedSpecimen

##### Distribution

[Distant, 1889/92] Continental India: Nepal; Ranikhet; Sikkim; Assam; Sylhet (Bangladesh). [Metcalf, 1963] Nepal; Northern Bengal; Assam; Northern India; Java; Sikkim; India; Bengal; Indochina; Malay Peninsula; Uttar Pradesh. [Duffels and van der Laan, 1985] Nepal; Bhutan. [Sanborn, 2014] India, Manipur, Assam, Naga Hills, Utta Pradesh, West Bengal, Indochina, Malay Peninsula, Nepal, Pakistan, Vietnam, Indonesia, Java, Bangladesh, Bhutan.

##### Notes

Authority: Walker 1858b

#### Macrosemia saturatavar.a

(Distant, 1891)

Cosmopsaltria saturata var. a Distant, 1891

##### Materials

- **Type status:** Holotype. **Occurrence:** recordedBy: William Doherty; **Taxon:** scientificName: Macrosemia saturata var. a (Distant, 1891); **Location:** continent: Asia; country: India; locality: Naga Hills; **Record Level:** basisOfRecord: PreservedSpecimen

##### Distribution

[Metcalf, 1963] Assam.

##### Notes

Authority: Distant 1891

#### Macrosemia saturatavar.b

(Distant, 1891)

Cosmopsaltria saturata var. b Distant, 1891

##### Materials

- **Type status:** Holotype. **Occurrence:** recordedBy: William Doherty; **Taxon:** scientificName: Macrosemia saturata var. b (Distant, 1891); **Location:** continent: Asia; country: India; locality: Naga Hills; **Record Level:** basisOfRecord: PreservedSpecimen

##### Distribution

[Metcalf, 1963] Assam.

##### Notes

Authority: Distant 1891

#### Macrosemia tonkiniana

(Jacobi, 1905)

Cosmopsaltria tonkiniana Jacobi, 1905

##### Materials

- **Type status:** Syntype. **Occurrence:** individualCount: 3; sex: male; **Taxon:** scientificName: Macrosemia tonkiniana (Jacobi, 1905); **Location:** continent: Asia; country: Vietnam; locality: Chiem-hoa; **Record Level:** basisOfRecord: PreservedSpecimen

##### Distribution

[Metcalf, 1963] Tonkin, Indochina, China [Duffels, 1985] Thailand [Sanborn, 2014] Vietnam, China, Yunnan, Hainan, Laos, Thailand, Myanmar, India, Indochina, Tonkin, Hainan.

##### Notes

Authority: Jacobi 1905; Sanborn (2014) states "India" in reference to Hua (2000), which may have been a mistranslation of Indochina, and to Lee (2008), which may be a repeat of the record from Hua (2000).

#### Macrosemia umbrata

(Distant, 1888)

Cosmopsaltria umbrata Distant, 1888
Macrosemia chantrainei Boulard, 2003

##### Materials

- **Type status:** Holotype. **Occurrence:** catalogNumber: BMNH(E) 1009471; individualCount: 1; sex: male; **Taxon:** scientificName: Macrosemia umbrata (Distant, 1888); **Location:** continent: Asia; country: India; locality: Sikkim; **Record Level:** institutionCode: NHMUK; basisOfRecord: PreservedSpecimen

##### Distribution

[Distant, 1889/92] Continental India: Sikkim; Naga Hills. Burma: Bhamo. [Metcalf, 1963] Sikkim; Burma; India; Assam; Northern Bengal; Upper Burma; Uttar Pradesh. [Duffels and van der Laan, 1985] Himalaya; Thailand; Nepal. [Sanborn, 2014] China, Yunnan, Jiangxi, Hainan, Burma, Sikkim, Nepal, India, Thailand, Sri Lanka, Bhutan, Laos, Manipur, Assam, Meghalaya, Uttar Pradesh, West Bengal, Myanmar, Pakistan, Himalayas.

##### Notes

Authority: Distant 1888c

#### Manna tenuis

Lee & Emery, 2013

Manna tenuis Lee & Emery, 2013

##### Materials

- **Type status:** Holotype. **Occurrence:** individualCount: 1; sex: male; **Taxon:** scientificName: Manna tenuis Lee & Emery, 2013; **Location:** continent: Asia; country: Tibet; locality: Motuo Co 108 km Bomi; verbatimElevation: 900 m; **Event:** eventDate: 2-8/07/2012; **Record Level:** institutionCode: MNHN; basisOfRecord: PreservedSpecimen
- **Type status:** Paratype. **Occurrence:** recordedBy: Bretschneider; individualCount: 1; sex: female; **Taxon:** scientificName: Manna tenuis Lee & Emery, 2013; **Location:** continent: Asia; country: India; locality: Arunachal Pradesh Dist. along near Rapum; verbatimElevation: 2000 m; **Event:** eventDate: 6-12/07/2010; **Record Level:** institutionCode: MNHN; basisOfRecord: PreservedSpecimen

##### Distribution

[Lee and Emery, 2013] Tibet; India.

##### Notes

Authority: Lee and Emery 2013; An additional 2 male and 9 female paratypes (AMS) were designated in the species description, however only material from India is included here.

#### Mata kama

(Distant, 1881)

Pomponia kama Distant, 1881

##### Materials

- **Type status:** Holotype. **Occurrence:** catalogNumber: BMNH(E) 1009460; individualCount: 1; sex: male; **Taxon:** scientificName: Mata kama (Distant, 1881); **Location:** continent: Asia; country: India; locality: Darjeeling, North India; **Record Level:** institutionCode: NHMUK; basisOfRecord: PreservedSpecimen

##### Distribution

[Distant, 1889/92] India: Darjeeling (North India). [Metcalf, 1963] Northern India; Bengal; India; Java; Malay States; Malaya. [Duffels and van der Laan, 1985] Nepal. [Sanborn, 2014] India.

##### Notes

Authority: Distant 1881; Species description states female, however the type specimen is male (Distant 1891).

#### Mata rama

Distant, 1912

Mata rama Distant, 1912

##### Materials

- **Type status:** Syntype. **Occurrence:** catalogNumber: BMNH(E) 1009461; recordedBy: R. Oberthur; individualCount: 1; sex: male; **Taxon:** scientificName: Mata rama Distant, 1912; **Location:** continent: Asia; country: Bhutan; locality: Bhutan; **Event:** eventDate: ??/??/1900; **Record Level:** institutionCode: NHMUK; basisOfRecord: PreservedSpecimen
- **Type status:** Syntype. **Occurrence:** catalogNumber: NHMUK 010214246; individualCount: 1; sex: female; **Taxon:** scientificName: Mata rama Distant, 1912; **Location:** continent: Asia; country: Bhutan; locality: Bhutan; **Event:** eventDate: ??/??/1900; **Record Level:** institutionCode: NHMUK; basisOfRecord: PreservedSpecimen

##### Distribution

[Metcalf, 1963] Bhutan; China. [Sanborn, 2014] China, Xizang, Bhutan.

##### Notes

Authority: Distant 1912a

#### Maua quadrituberculata tavoyana

(Ollenbach, 1929)

Purana tavoyana Ollenbach, 1929
Maua quadrituberculata khunpaworensis Boulard, 2007

##### Materials

- **Type status:** Syntype. **Occurrence:** recordedBy: W.S. Wood; individualCount: 1; sex: male; **Taxon:** scientificName: Maua quadrituberculata tavoyana (Ollenbach, 1929); **Location:** continent: Asia; country: Myanmar; locality: Tavoy [now Dawei]; **Event:** eventDate: ??/09/1925; **Record Level:** institutionCode: IFRI; basisOfRecord: PreservedSpecimen
- **Type status:** Syntype. **Occurrence:** recordedBy: W.S. Wood; individualCount: 5; sex: female; **Taxon:** scientificName: Maua quadrituberculata tavoyana (Ollenbach, 1929); **Location:** continent: Asia; country: Myanmar; locality: Tavoy [now Dawei]; **Event:** eventDate: ??/09/1925; **Record Level:** institutionCode: IFRI; basisOfRecord: PreservedSpecimen

##### Distribution

[Metcalf, 1963] Burma; India. [Sanborn, 2014] Burma, Thailand.

##### Notes

Authority: Ollenbach 1929; Not from India: Metcalf (1963) listed India in reference to Mathur (1953), in which it states "India.-Burma: Tavoy" with Burma being considerd part of "British India".

#### Megapomponia intermedia

(Distant, 1905)

Pomponia intermedia Distant, 1905

##### Materials

- **Type status:** Holotype. **Occurrence:** catalogNumber: BMNH(E) 1009388; recordedBy: C.T. Bingham; individualCount: 1; sex: male; **Taxon:** scientificName: Megapomponia intermedia (Distant, 1905); **Location:** continent: Asia; country: Myanmar; locality: Thaungyang Valley, Tennasserim; **Record Level:** institutionCode: NHMUK; basisOfRecord: PreservedSpecimen

##### Distribution

[Metcalf, 1963] Tenasserim; Indochina; India; Burma. [Sanborn, 2014] Thailand, India, Burma, Indochina, Laos, Myanmar, Vietnam.

##### Notes

Authority: Distant 1905a; Not from India: Metcalf (1963) listed India in reference to Mathur (1953), in which it references a specimen "India.-Burma: Tavoy" with Burma being considerd part of "British India". The type locality (Thaungyang Valley, Tennasserim) and Mathur's locality Tavoy (now Dawei) are both in the far south of Myanmar thus this species has been removed from the Indian list. Subsequent "India" localities as stated in Sanborn (2014), in reference to Boulard (2001b), Sanborn et al. (2007) and Lee (2008), are likely to have resulted from this initial error. Hayashi (1993) excluded this species from India.

#### Meimuna cassandra

Distant, 1912

Meimuna cassandra Distant, 1912

##### Materials

- **Type status:** Holotype. **Occurrence:** catalogNumber: BMNH(E) 1009441; individualCount: 1; sex: male; **Taxon:** scientificName: Meimuna cassandra Distant, 1912; **Location:** continent: Asia; country: India; locality: near Dehra Dun, N. India; **Event:** eventDate: 28/06/1911; **Record Level:** institutionCode: NHMUK; basisOfRecord: PreservedSpecimen

##### Distribution

[Metcalf, 1963] United Provinces; Northern India.

##### Notes

Authority: Distant 1912c

#### Meimuna gamameda

(Distant, 1902)

Cosmopsaltria gamameda Distant, 1902

##### Materials

- **Type status:** Syntype. **Occurrence:** catalogNumber: BMNH(E) 1009476; recordedBy: E.E. Green; sex: male; **Taxon:** scientificName: Meimuna gamameda (Distant, 1902); **Location:** continent: Asia; country: Sri Lanka; locality: Pundalu-oya; **Event:** eventDate: ??/02/1902; **Record Level:** institutionCode: NHMUK; basisOfRecord: PreservedSpecimen
- **Type status:** Syntype. **Occurrence:** recordedBy: E.E. Green; sex: female; **Taxon:** scientificName: Meimuna gamameda (Distant, 1902); **Location:** continent: Asia; country: Sri Lanka; locality: Pundalu-oya; **Event:** eventDate: ??/02/1902; **Record Level:** basisOfRecord: PreservedSpecimen
- **Type status:** Other material. **Occurrence:** catalogNumber: BMNH(E) 1009443; individualCount: 1; sex: male; **Taxon:** scientificName: Meimuna gamameda (Distant, 1902); **Location:** continent: Asia; country: Sri Lanka; locality: Kandy, Ceylon; **Record Level:** institutionCode: NHMUK; basisOfRecord: PreservedSpecimen

##### Distribution

[Metcalf, 1963] Ceylon. [Duffels and van der Laan, 1985] Ceylon. [Sanborn, 2014] China, Yunnan, Sikkim, Sri Lanka.

##### Notes

Authority: Distant 1902; Sanborn (2014) listed locality including Sikkim in reference to Lei (1994). This may be in error as the species has been recorded from Sri Lanka previously.

#### Meimuna microdon

(Walker, 1850)

Dundubia microdon Walker, 1850
Meimuna omeinensis Chen, 1940

##### Materials

- **Type status:** Holotype. **Occurrence:** catalogNumber: BMNH(E) 1009440; recordedBy: Dr Wallich; sex: male; **Taxon:** scientificName: Meimuna microdon (Walker, 1850); **Location:** continent: Asia; country: India; locality: N. India; **Record Level:** institutionCode: NHMUK; basisOfRecord: PreservedSpecimen

##### Distribution

[Distant, 1889/92] India: North India; Sikkim. [Metcalf, 1963] India; Northern India; Tonkin; Sikkim; Indochina. [Sanborn, 2014] China, Yunnan, Tibet, India, Sikkim, Vietnam, South China, India, Hainan.

##### Notes

Authority: Walker 1850

#### Meimuna pallida

Ollenbach, 1929

Meimuna pallida Ollenbach, 1929

##### Materials

- **Type status:** Holotype. **Occurrence:** sex: male; **Taxon:** scientificName: Meimuna pallida Ollenbach, 1929; **Location:** continent: Asia; country: India; locality: Kumaon, Tarakhet, United Provinces; **Record Level:** institutionCode: IFRI; basisOfRecord: PreservedSpecimen

##### Distribution

[Metcalf, 1963] United Provinces; India; Uttar Pradesh.

##### Notes

Authority: Ollenbach 1929

#### Meimuna silhetana

(Distant, 1888)

Cosmopsaltria silhetana Distant, 1888

##### Materials

- **Type status:** Holotype. **Occurrence:** catalogNumber: BMNH(E) 1009450; individualCount: 1; sex: male; **Taxon:** scientificName: Meimuna silhetana (Distant, 1888); **Location:** continent: Asia; country: Bangladesh; locality: Silhet; **Record Level:** institutionCode: NHMUK; basisOfRecord: PreservedSpecimen

##### Distribution

[Metcalf, 1963] Assam; India; Kwangtung; China; Fukien. [Sanborn, 2014] China, Fujian, Guangdong, Sichuan, Yunnan, India, Hong Kong, Yunnan.

##### Notes

Authority: Distant 1888c

#### Meimuna tavoyana

(Distant, 1888)

Dundubia tavoyana Distant, 1888

##### Materials

- **Type status:** Holotype. **Taxon:** scientificName: Meimuna tavoyana (Distant, 1888); **Location:** continent: Asia; country: Myanmar; locality: Tavoy (now Dawei), Tenasserim; **Record Level:** institutionCode: NZSI; basisOfRecord: PreservedSpecimen
- **Type status:** Other material. **Occurrence:** catalogNumber: BMNH(E) 1009444; occurrenceRemarks: Specimen not part of type series, see taxon notes.; recordedBy: C.T. Bingham; individualCount: 1; sex: male; **Taxon:** scientificName: Meimuna tavoyana (Distant, 1888); **Location:** continent: Asia; country: Myanmar; locality: Burma; **Record Level:** institutionCode: NHMUK; basisOfRecord: PreservedSpecimen

##### Distribution

[Distant, 1889/92] Burma: Tavoy (Tenasserim). [Metcalf, 1963] Burma; Tenasserim; Malay Peninsula; Pahang; Thailand. [Sanborn, 2014] Thailand, Tenasserim, Burma, Peninsular Malaysia, Yunnan, Malaysia.

##### Notes

Authority: Distant 1888c; In the NHMUK there is a male specimen bearing a type label, and a note from Beuk dated 1995. This male was described later from Burma by Distant (1891). The type is in the NZSI and is from "Tavoy".

#### Meimuna tripurasura

(Distant, 1881)

Dundubia tripurasura Distant, 1881

##### Materials

- **Type status:** Holotype. **Occurrence:** catalogNumber: BMNH(E) 1009442; recordedBy: A.W. Chennell; individualCount: 1; sex: male; **Taxon:** scientificName: Meimuna tripurasura (Distant, 1881); **Location:** continent: Asia; country: India; locality: N. Khasi Hills, British Assam; verbatimElevation: 1500 - 3000 ft; **Record Level:** institutionCode: NHMUK; basisOfRecord: PreservedSpecimen

##### Distribution

[Distant, 1906] India: Sikkim; Assam, Margherita and N. Khasi Hills. [Metaclf, 1963] Assam; India; Tonkin; Sikkim; Bengal; Indochina; Kwangsi; China; Kwangtung; Madras; Northern Bengal. [Sanborn, 2014] China, Kwangtung, Kwangsi, Sikhim, Assam, Margherita, Khasi Hills, Sichuan, Yunnan, Sri Lanka, Vietnam, South China, India.

##### Notes

Authority: Distant 1881

#### Meimuna velitaris

(Distant, 1897)

Cosmopsaltria velitaris Distant, 1897

##### Materials

- **Type status:** Lectotype. **Occurrence:** catalogNumber: BMNH(E) 1009458; recordedBy: Watson; individualCount: 1; sex: male; **Taxon:** scientificName: Meimuna velitaris (Distant, 1897); **Location:** continent: Asia; country: Myanmar; locality: North Chin Hills; **Record Level:** institutionCode: NHMUK; basisOfRecord: PreservedSpecimen

##### Distribution

[Metcalf, 1963] Burma; India. [Sanborn, 2014] Pakistan, Burma.

##### Notes

Authority: Distant 1897; Metcalf (1963) listed India in reference to Mathur (1953) in which Burma was considerd a part of "British India". The type locality (Chin Hills) extends into India (Lushai Hills - Nagaland), thus this species may be recorded in Nagaland in future.

#### Melampsalta literata

(Distant, 1888)

Cicadetta literata Distant, 1888

##### Materials

- **Type status:** Holotype. **Occurrence:** catalogNumber: BMNH(E) 1009614; recordedBy: Leech; individualCount: 1; sex: female; **Taxon:** scientificName: Melampsalta literata (Distant, 1888); **Location:** continent: Asia; country: India; locality: Cashmere Valley; verbatimElevation: 6300 ft; **Record Level:** institutionCode: NHMUK; basisOfRecord: PreservedSpecimen

##### Distribution

[Metcalf, 1963] Kashmir; India; Uttar Pradesh. [Sanborn, 2014] Pakistan, India.

##### Notes

Authority: Distant 1888d

#### Mogannia aurea

Fraser, 1942

Mogannia aurea Fraser, 1942

##### Materials

- **Type status:** Holotype. **Occurrence:** recordedBy: Lindgren; individualCount: 1; sex: male; **Taxon:** scientificName: Mogannia aurea Fraser, 1942; **Location:** continent: Asia; country: India; locality: Kurseong, Sikkim; **Record Level:** basisOfRecord: PreservedSpecimen

##### Distribution

[Metcalf, 1963] Sikkim.

##### Notes

Authority: Fraser 1942

#### Mogannia conica conica

(Germar, 1830)

Cicada conica Germar, 1830
Mogannia conica conica (Germar, 1830)
Mogannia illustrata Amyot & Audinet-Serville, 1843
Cephaloxys hemelytra Signoret, 1847
Mogannia indicans Walker, 1850
Mogannia conica conica Mogannia incidans var. β Walker, 1850
Mogannia ignifera Walker, 1850
Mogannia avicula Walker, 1850
Mogannia recta Walker, 1858
Mogannia histrionica Uhler, 1861

##### Materials

- **Type status:** Holotype. **Occurrence:** recordedBy: Westermann; **Taxon:** scientificName: Mogannia conica conica (Germar, 1830); **Location:** continent: Asia; country: Indonesia; locality: Java; **Record Level:** institutionCode: NHMW; basisOfRecord: PreservedSpecimen

##### Distribution

[Metcalf, 1963] Java; Philippine Island; Hindustan; India; Tenasserim; China; Sumatra; Assam; Malay Archipelago; Hong Kong Island; Palawan; Malaya; Indochina; Kwangtung; Tonkin. [Duffels and van der Laan, 1985] Nepal; China. [Sanborn, 2014] China, Assam, Khasi Hills, Margherita, Tenasserim, Thaga, Java, Sumatra, Philippines, Tonkin, Malaysia, Guangdong, Hong Kong, Yunnan, Xizang, Indonesia, Vietnam, India, Nepal, Southeast Asia, Thailand, Philippine Republic, Malayan Archipelago, North Vietnam, Palawan.

##### Notes

Authority: Germar 1830

#### Mogannia cyanea

Walker, 1858

Mogannia cyanea Walker, 1858
Mogannia cyanea Walker, 1858
Mogannia nigrocyanea Matsumura, 1913
Mogannia nigrocyanea flavofascia Kato, 1925
Mogannia bella Kato, 1927
Mogannia chekingensis Ouchi, 1938
Mogannia chinensis Ouchi, 1938 (nec Stål, 1865)
Mogannia tienmushana Chen, 1957
Mogannia ouchii Metcalf, 1963

##### Materials

- **Type status:** Holotype. **Occurrence:** catalogNumber: BMNH(E) 1009578; sex: male; **Taxon:** scientificName: Mogannia cyanea Walker, 1858; **Location:** continent: Asia; country: China; locality: North China; **Record Level:** institutionCode: NHMUK; basisOfRecord: PreservedSpecimen

##### Distribution

[Distant, 1889/92]: India: Naga Hills and Margherita (Assam). Burma: Ruby Mines. [Metcalf, 1963]: Northern China, India Burma; China; Assam; Formosa; Oriental Region; Japan; Indochina; Kwangtung; Chekiang; Eastern China; Kwangsi; Fukien. [Duffels and van der Laan, 1985] Taiwan; China; India; Assam; Burma. [Sanborn, 2014] India: Assam, Margherita, Naga Hills; China: Hunan, Zhejiang, Liaoning, Shanxi, Gansu, Hubei, Jiangsu, Jiangxi, Fujian, Guangdong, Guangxi, Sichuan, Yunnan; Burma: , Arisan, Taiwan (Formosa); Hong Kong; Korea, Thailand, Indochina, Southeast Asia, Vietnam.

##### Notes

Authority: Walker 1858b

#### Mogannia effecta effecta

Distant, 1892

Mogannia effecta Distant, 1892

##### Materials

- **Type status:** Holotype. **Occurrence:** catalogNumber: BMNH(E) 1009579; sex: male; **Taxon:** scientificName: Mogannia effecta effecta Distant, 1892; **Location:** continent: Asia; country: India; locality: N.E. India; **Record Level:** institutionCode: NHMUK; basisOfRecord: PreservedSpecimen

##### Distribution

[Distant, 1889/92] India: Sikkim; Darjeeling; Naga Hills. [Metcalf, 1963] Sumatra; Northeastern India; India; Sikkim; Assam; Bengal; Northern Bengal; Sumatra (?); Uttar Pradesh. [Duffels and van der Laan, 1985] Nepal. [Sanborn, 2014] China, Vietnam, Nepal, India, Indonesia.

##### Notes

Authority: Distant 1892b

#### Mogannia effectavar.a

Distant, 1892

Mogannia effecta var. a Distant, 1892

##### Materials

- **Type status:** Holotype. **Taxon:** scientificName: Mogannia effecta var. a Distant, 1892; **Location:** continent: Asia; country: Indonesia; locality: Sumatra; **Record Level:** basisOfRecord: PreservedSpecimen

##### Distribution

[Distant, 1889/92] India: Sikkim; Darjeeling; Naga Hills. [Metcalf, 1963] Sumatra; Northeastern India; India; Sikkim; Assam; Bengal; Northern Bengal; Sumatra (?); Uttar Pradesh.

##### Notes

Authority: Distant 1892b

#### Mogannia effectavar.b

Distant, 1892

Mogannia effecta var. b Distant, 1892

##### Materials

- **Type status:** Holotype. **Taxon:** scientificName: Mogannia effecta var. b Distant, 1892; **Location:** continent: Asia; country: Indonesia; locality: Sumatra; **Record Level:** basisOfRecord: PreservedSpecimen

##### Distribution

[Distant, 1889/92] India: Sikkim; Darjeeling; Naga Hills. [Metcalf, 1963] Sumatra; Northeastern India; India; Sikkim; Assam; Bengal; Northern Bengal; Sumatra (?); Uttar Pradesh.

##### Notes

Authority: Distant 1892b

#### Mogannia funebris

Stål, 1865

Mogannia funebris Stål, 1865

##### Materials

- **Type status:** Holotype. **Occurrence:** sex: female; **Taxon:** scientificName: Mogannia funebris Stål, 1865; **Location:** continent: Asia; country: Bangladesh; locality: Silhet; **Record Level:** institutionCode: NHRS; basisOfRecord: PreservedSpecimen
- **Type status:** Other material. **Occurrence:** catalogNumber: BMNH(E) 1009580; recordedBy: R.V. de Salvaza; individualCount: 1; sex: male; **Taxon:** scientificName: Mogannia funebris Stål, 1865; **Location:** continent: Asia; country: Laos; locality: Xieng Khomang; **Event:** eventDate: 18/05/1919; **Record Level:** institutionCode: NHMUK; basisOfRecord: PreservedSpecimen

##### Distribution

[Metcalf, 1963] Assam; Silhet; India; Burma; Tonkin; British India; Indochina; Uttar Pradesh. [Sanborn, 2014] Vietnam, Myanmar, Bangladesh, India.

##### Notes

Authority: Stål 1865

#### Mogannia funebrisvar.a

Distant, 1892

Mogannia funebris var. a Distant, 1892

##### Materials

- **Type status:** Holotype. **Occurrence:** recordedBy: Leonardo Fea; **Taxon:** scientificName: Mogannia funebris var. a Distant, 1892; **Location:** continent: Asia; country: Myanmar; locality: Bhamo, Burma; **Record Level:** institutionCode: MSNG; basisOfRecord: PreservedSpecimen

##### Distribution

[Metcalf, 1963] Burma.

##### Notes

Authority: Distant 1892a

#### Mogannia nasalis nasalis

(White, 1844)

Cicada nasalis White, 1844
Mogannia chinensis Stål, 1865
Mogannia kikowensis Ouchi, 1938
Mogannia kikowensis var. a Ouchi, 1938
Mogannia kikowensis var. b Ouchi, 1938
Mogannia kikowensis var. c Ouchi, 1938

##### Materials

- **Type status:** Holotype. **Occurrence:** recordedBy: J.C. Bowring; **Taxon:** scientificName: Mogannia nasalis nasalis (White, 1844); **Location:** continent: Asia; country: China; locality: Hong Kong; **Record Level:** basisOfRecord: PreservedSpecimen

##### Distribution

[Metcalf, 1963] Hong Kong Island; Fukien; China; Northern China; Formosa; Macao Island; Oriental Region; Japan; Southern China; Kwangtung; Assam; Anhwei. [Duffels and van der Laan, 1985] China. [Sanborn, 2014] China, Macao, Hongkong, Assam, Formosa, Anhui, Zhejiang, Fujian, Taiwan, Hunan, Guangxi, India.

##### Notes

Authority: White 1844

#### Mogannia obliqua

Walker, 1858

Mogannia obliqua Walker, 1858

##### Materials

- **Type status:** Syntype. **Occurrence:** catalogNumber: BMNH(E) 1009581; recordedBy: J.C. Bowring; sex: female; **Taxon:** scientificName: Mogannia obliqua Walker, 1858; **Location:** continent: Asia; country: Indonesia; locality: Java (E. Indies); **Record Level:** institutionCode: NHMUK; basisOfRecord: PreservedSpecimen
- **Type status:** Other material. **Occurrence:** catalogNumber: BMNH(E) 1009582; recordedBy: Archbald; individualCount: 1; sex: female; **Taxon:** scientificName: Mogannia obliqua Walker, 1858; **Location:** continent: Asia; country: India; locality: Karen Hill Tracts; **Event:** eventDate: ??/05-06/1923; **Record Level:** institutionCode: NHMUK; basisOfRecord: PreservedSpecimen
- **Type status:** Syntype. **Occurrence:** sex: female; **Taxon:** scientificName: Mogannia obliqua Walker, 1858; **Location:** continent: Asia; country: India; locality: Hindostan; **Record Level:** basisOfRecord: PreservedSpecimen

##### Distribution

[Distant, 1889/92] India: Sikkim; Assam; Naga Hills; Margherita; Mungpoo (Bengal). Burma: Momeit; Rangoon; Charin. [Metcalf, 1963] Hindustan; Java; India; Burma; Sikkim; Assam; Bengal; Malay States; Malaya; Indochina; Malay Peninsula; East Indies; Tenasserim; Northern Bengal; Upper Burma; Indo-Malay Peninsula. [Sanborn, 2014] China, Borneo, Sabah, Thailand, India, Malaysian Archipelago, Java, Bengal, Burma, Malaysia, Vietnam, Indonesia, Myanmar, Vietnam.

##### Notes

Authority: Walker 1858b

#### Mogannia venutissimavar.a

Stål, 1865

Mogannia venutissima var. a Stål, 1865

##### Materials

- **Type status:** Holotype. **Taxon:** scientificName: Mogannia venutissima var. a Stål, 1865; **Location:** continent: Asia; locality: India orientalis; **Record Level:** basisOfRecord: PreservedSpecimen

##### Distribution

[Metcalf, 1963] Eastern India.

##### Notes

Authority: Stål 1865

#### Mogannia venutissimavar.b

Stål, 1865

Mogannia venutissima var. b Stål, 1865

##### Materials

- **Type status:** Holotype. **Taxon:** scientificName: Mogannia venutissima var. b Stål, 1865; **Location:** continent: Asia; locality: India orientalis; **Record Level:** basisOfRecord: PreservedSpecimen

##### Distribution

[Metcalf, 1963] Eastern India.

##### Notes

Authority: Stål 1865

#### Mogannia venutissima venutissima

Stål, 1865

Mogannia venutissima Stål, 1865

##### Materials

- **Type status:** Syntype. **Occurrence:** sex: male; **Taxon:** scientificName: Mogannia venutissima venutissima Stål, 1865; **Location:** continent: Asia; locality: India orientalis; **Record Level:** institutionCode: NHRS; basisOfRecord: PreservedSpecimen
- **Type status:** Syntype. **Occurrence:** sex: female; **Taxon:** scientificName: Mogannia venutissima venutissima Stål, 1865; **Location:** continent: Asia; locality: India orientalis; **Record Level:** institutionCode: NHRS; basisOfRecord: PreservedSpecimen

##### Distribution

[Metcalf, 1963] Eastern India; India; Malaya; Malay Archipelago; Sikkim; Malay Peninsula; Krakatau Islands. [Sanborn, 2014] Indonesia, Krakatau.

##### Notes

Authority: Stål 1865

#### Mogannia viridis

(Signoret, 1847)

Cephaloxys viridis Signoret, 1847
Cephaloxys rostrata Walker, 1850
Mogannia distinguenda Distant, 1920

##### Materials

- **Type status:** Holotype. **Occurrence:** sex: male; **Taxon:** scientificName: Mogannia viridis (Signoret, 1847); **Location:** continent: Asia; country: Indonesia; locality: Java; **Record Level:** basisOfRecord: PreservedSpecimen

##### Distribution

[Distant, 1889/92] India: Mungpoo (Bengal); Naga Hills and Margherita (Assam). Burma: Momeit. [Metcalf, 1963] Java; India; Burma; Malay Peninsula; Bengal; Assam; Malay States; Banguey Island; Malaya; Perak; Borneo; Malacca; Singapore Island; Johore; Penang; Tenasserim; Indochina; Philippine Islands; Sikkm; Sumatra; Upper Burma; Malay Archipelago. [Sanborn, 2014] Borneo, Sarawak, Java, India, Burma, Peninsular Malaysia, Banguey Island, Philippines, Sabah, Banguey Island, Vietnam, Indonesia, Sumatra, Myanmar.

##### Notes

Authority: Signoret 1847b

#### Mosaica irregularis

Lee & Emery, 2013

Mosaica irregularis Lee & Emery, 2013

##### Materials

- **Type status:** Holotype. **Occurrence:** recordedBy: Bretschneider; individualCount: 1; sex: male; **Taxon:** scientificName: Mosaica irregularis Lee & Emery, 2013; **Location:** continent: Asia; country: India; locality: Arunachal Pradesh Dist. along near Rapum; verbatimElevation: 2000 m; **Event:** eventDate: 6-12/07/2010; **Record Level:** institutionCode: MNHN; basisOfRecord: PreservedSpecimen

##### Distribution

[Lee and Emery, 2013] India.

##### Notes

Authority: Lee and Emery 2013

#### Tanna thalia

(Walker, 1850)

Dundubia thalia Walker, 1850
Cicada sphinx Walker, 1850
Pomponia horsfieldi Distant, 1893

##### Materials

- **Type status:** Syntype. **Occurrence:** catalogNumber: BMNH(E) 1009406; occurrenceRemarks: Voucher number corresponds to one specimen.; individualCount: 3; sex: male; **Taxon:** scientificName: Tanna thalia (Walker, 1850); **Location:** locality: Locality unknown; **Record Level:** institutionCode: NHMUK; basisOfRecord: PreservedSpecimen

##### Distribution

[Distant, 1906] Continental India: Sikkim; Mussoore; Darjeeling. [Metcalf, 1963] Borneo; India; Java; Sikkim; Mysore; Bengal; Tibet; Sarawak; British India; Yunnan; Northern India; China; Assam; Uttar Pradesh. [Duffels and van der Laan, 1985] Tibet; China; Nepal; China. [Sanborn, 2014] China, Yunna, Tibet, India, Sikkim, Darjiling, Yunnan, Sichuan, Xizang, Indonesia, Java.

##### Notes

Authority: Walker 1850

#### Neoterpnosia donghai

Lee & Emery, 2014

Neoterpnosia donghai Lee & Emery, 2014

##### Materials

- **Type status:** Holotype. **Occurrence:** catalogNumber: MNHN (EH) 16438; recordedBy: G. Bretschneider; individualCount: 1; sex: male; **Taxon:** scientificName: Neoterpnosia donghai Lee & Emery, 2014; **Location:** continent: Asia; country: India; locality: Monigong, Lungte, 1225m, Arunachal Pradesh; verbatimElevation: 1225 m; **Record Level:** institutionCode: MNHN; basisOfRecord: PreservedSpecimen
- **Type status:** Paratype. **Occurrence:** recordedBy: G. Bretschneider; individualCount: 2; sex: male; **Taxon:** scientificName: Neoterpnosia donghai Lee & Emery, 2014; **Location:** continent: Asia; country: India; locality: Monigong, Lungte, 1225m, Arunachal Pradesh; verbatimElevation: 1225 m; **Record Level:** institutionCode: AMS; basisOfRecord: PreservedSpecimen
- **Type status:** Paratype. **Occurrence:** recordedBy: G. Bretschneider; individualCount: 1; sex: female; **Taxon:** scientificName: Neoterpnosia donghai Lee & Emery, 2014; **Location:** continent: Asia; country: India; locality: Monigong, Lungte, 1225m, Arunachal Pradesh; verbatimElevation: 1225 m; **Record Level:** institutionCode: AMS; basisOfRecord: PreservedSpecimen

##### Distribution

[Lee and Emery, 2014] India.

##### Notes

Authority: Lee and Emery 2014a

#### Neoterpnosia oberthuri

(Distant, 1912)

Terpnosia oberthuri Distant, 1912

##### Materials

- **Type status:** Holotype. **Occurrence:** catalogNumber: BMNH(E) 1009550; recordedBy: Mr Durel (R. Oberthur coll.); individualCount: 1; sex: male; **Taxon:** scientificName: Neoterpnosia oberthuri (Distant, 1912); **Location:** continent: Asia; country: Bhutan; locality: Maria-Basti; **Event:** eventDate: ??/??/1898; **Record Level:** institutionCode: NHMUK; basisOfRecord: PreservedSpecimen

##### Distribution

[Metcalf, 1963] Bhutan; China.

##### Notes

Authority: Distant 1912a

#### Neoterpnosia versicolor

(Distant, 1912)

Terpnosia versicolor Distant, 1912

##### Materials

- **Type status:** Holotype. **Occurrence:** catalogNumber: BMNH(E) 1009551; recordedBy: William Doherty; individualCount: 1; sex: male; **Taxon:** scientificName: Neoterpnosia versicolor (Distant, 1912); **Location:** continent: Asia; country: Myanmar; locality: Ruby Mines, Burma; **Record Level:** institutionCode: NHMUK; basisOfRecord: PreservedSpecimen

##### Distribution

[Metcalf, 1963] Burma. [Duffels and van der Laan, 1985] Nepal.

##### Notes

Authority: Distant 1912a

#### Oxypleura atkinsoni

(Distant, 1912)

Platypleura atkinsoni Distant, 1912

##### Materials

- **Type status:** Holotype. **Occurrence:** catalogNumber: BMNH(E) 1009501; recordedBy: E.F.T. Atkinson; individualCount: 1; sex: female; **Taxon:** scientificName: Oxypleura atkinsoni (Distant, 1912); **Location:** continent: Asia; country: Myanmar; locality: Tenasserim; **Record Level:** institutionCode: NHMUK; basisOfRecord: PreservedSpecimen

##### Distribution

[Metcalf, 1963] Tenasserim.

##### Notes

Authority: Distant 1912d

#### Paharia lacteipennis

(Walker, 1850)

Cephaloxys lacteipennis Walker, 1850

##### Materials

- **Type status:** Holotype. **Occurrence:** catalogNumber: BMNH(E) 1009602; individualCount: 1; sex: male; **Taxon:** scientificName: Paharia lacteipennis (Walker, 1850); **Location:** continent: Asia; country: India; locality: N. India; **Record Level:** institutionCode: NHMUK; basisOfRecord: PreservedSpecimen

##### Distribution

[Metcalf, 1963] India; Northern India; Afghanistan; Baluchistan. [Duffels and van der Laan, 1985] Afghanistan; Kazakhstan (U.S.S.R.); Kashmir. [Sanborn, 2014] Pakistan, Kazakhstan, Afghanistan, India.

##### Notes

Authority: Walker 1850; Walker (1850) specified the type as a male in the species description, Distant (1892a) later states, in error, that the type was a female.

#### Panka simulata

Distant, 1905

Panka simulata Distant, 1905
Tibicen nubifurca Distant, 1892 (nec Walker, 1858)

##### Materials

- **Type status:** Holotype. **Taxon:** scientificName: Panka simulata Distant, 1905; **Location:** continent: Asia; country: Sri Lanka; locality: Ceylon; **Record Level:** basisOfRecord: PreservedSpecimen

##### Distribution

[Metcalf, 1963] Ceylon. [Duffels and van der Laan, 1985] Ceylon. [Sanborn, 2014] Ceylon.

##### Notes

Authority: Distant 1905d

#### Paranosia andersoni

(Distant, 1892)

Terpnosia andersoni Distant, 1892

##### Materials

- **Type status:** Holotype. **Occurrence:** catalogNumber: BMNH(E) 1009545; recordedBy: Dr Anderson; individualCount: 1; sex: male; **Taxon:** scientificName: Paranosia andersoni (Distant, 1892); **Location:** continent: Asia; country: China; locality: W. Yunnan, China; **Record Level:** institutionCode: NHMUK; basisOfRecord: PreservedSpecimen

##### Distribution

[Metcalf, 1963] China; Yunnan; Sikkim; Burma; Tenasserim; Western Yunnan; Kiangsu; India. [Duffels and van der Laan, 1985] Thailand; Nepal; Bhutan; China. [Sanborn, 2014] China, Jiangsu, Jiangxi, Yunnan, Burma, India, Nepal, Bhutan, Thailand, Laos, Southeast Asia.

##### Notes

Authority: Distant 1892c

#### Paratanna parata

Lee, 2012

Paratanna parata Lee, 2012

##### Materials

- **Type status:** Holotype. **Occurrence:** individualCount: 1; sex: male; **Taxon:** scientificName: Paratanna parata Lee, 2012; **Location:** continent: Asia; country: India; locality: Shembaganur, Madura, Indes anglaises; **Record Level:** institutionCode: IRSNB; basisOfRecord: PreservedSpecimen
- **Type status:** Paratype. **Occurrence:** individualCount: 1; sex: male; **Taxon:** scientificName: Paratanna parata Lee, 2012; **Location:** continent: Asia; country: India; locality: Shembaganur, Indes anglaises; **Record Level:** institutionCode: IRSNB; basisOfRecord: PreservedSpecimen
- **Type status:** Paratype. **Occurrence:** individualCount: 1; sex: male; **Taxon:** scientificName: Paratanna parata Lee, 2012; **Location:** continent: Asia; country: India; locality: Shamba, ganur; **Record Level:** institutionCode: IRSNB; basisOfRecord: PreservedSpecimen
- **Type status:** Paratype. **Occurrence:** recordedBy: R. De Buck; individualCount: 2; sex: female; **Taxon:** scientificName: Paratanna parata Lee, 2012; **Location:** continent: Asia; country: India; locality: Indes anglaises, Madura, Shembaganur; **Record Level:** institutionCode: IRSNB; basisOfRecord: PreservedSpecimen
- **Type status:** Paratype. **Occurrence:** individualCount: 1; sex: female; **Taxon:** scientificName: Paratanna parata Lee, 2012; **Location:** continent: Asia; country: India; locality: Shembaganur, Madura, Indes; **Record Level:** institutionCode: IRSNB; basisOfRecord: PreservedSpecimen

##### Distribution

[Lee, 2012] India.

##### Notes

Authority: Lee 2012a

#### Pauropsalta exaequata

(Distant, 1892)

Melampsalta exaequata Distant, 1892

##### Materials

- **Type status:** Holotype. **Occurrence:** catalogNumber: BMNH(E) 1009616; recordedBy: William Doherty; individualCount: 1; sex: female; **Taxon:** scientificName: Pauropsalta exaequata (Distant, 1892); **Location:** continent: Asia; country: India; locality: Naga Hills; **Record Level:** institutionCode: NHMUK; basisOfRecord: PreservedSpecimen

##### Distribution

[Metcalf, 1963] India; Assam. [Sanborn, 2014] India.

##### Notes

Authority: Distant 1892c

#### Platylomia amicta

(Distant, 1889)

Dundubia amicta Distant, 1889

##### Materials

- **Type status:** Holotype. **Occurrence:** catalogNumber: BMNH(E) 1009473; recordedBy: E.F.T. Atkinson; individualCount: 1; sex: male; **Taxon:** scientificName: Platylomia amicta (Distant, 1889); **Location:** continent: Asia; country: India; locality: Karwar (Karnataka); **Record Level:** institutionCode: NHMUK; basisOfRecord: PreservedSpecimen

##### Distribution

[Metcalf, 1963] Bombay; India.

##### Notes

Authority: Distant 1889a

#### Platylomia brevis

Distant, 1912

Platylomia brevis Distant, 1912

##### Materials

- **Type status:** Holotype. **Occurrence:** catalogNumber: BMNH(E) 1009478; individualCount: 1; sex: male; **Taxon:** scientificName: Platylomia brevis Distant, 1912; **Location:** continent: Asia; country: India; locality: N. India; **Record Level:** institutionCode: NHMUK; basisOfRecord: PreservedSpecimen

##### Distribution

[Metcalf, 1963] Northern India; Himalayas. [Duffels and van der Laan, 1985] Nepal.

##### Notes

Authority: Distant 1912d

#### Platylomia ficulnea

(Distant, 1892)

Cosmopsaltria ficulnea Distant, 1892

##### Materials

- **Type status:** Lectotype. **Occurrence:** recordedBy: Leonardo Fea; individualCount: 1; sex: male; **Taxon:** scientificName: Platylomia ficulnea (Distant, 1892); **Location:** continent: Asia; country: Myanmar; locality: Carin Ghecu; verbatimElevation: 1300 - 1400 m; **Record Level:** institutionCode: MSNG; basisOfRecord: PreservedSpecimen
- **Type status:** Paralectotype. **Occurrence:** catalogNumber: BMNH(E) 1009484; occurrenceRemarks: Not Lectotype, see taxon notes.; recordedBy: William Doherty; individualCount: 1; sex: male; **Taxon:** scientificName: Platylomia ficulnea (Distant, 1892); **Location:** continent: Asia; country: Myanmar; locality: Karen Hills; **Record Level:** institutionCode: NHMUK; basisOfRecord: PreservedSpecimen

##### Distribution

[Distant, 1906] India: Assam. Burma: Karennee; Karen Hills. [Metcalf, 1963] Burma; Assam. [Sanborn, 2014] Burma, India.

##### Notes

Authority: Distant 1892c; Beuk (1998) designated a male Lectotype: "Lectotype [male] (here desginated) of Cosmopsaltria ficulnea Distant: 'Carin Ghecu / 1300-1400 m / L. Fea II-III.88' [printed] and 'Cosmopsaltria / ficulnea / type Dist.' [Distant's handwriting]". Hence the specimen labelled as the Lectotype in NHMUK is actually the Paralectotype. An additional 2 male (1MSNG, 1 NHMUK) paralectotypes were designated.

#### Platylomia insignis

Distant, 1912

Platylomia insignis Distant, 1912

##### Materials

- **Type status:** Holotype. **Occurrence:** catalogNumber: BMNH(E) 1009472; recordedBy: Mr Durel (R. Oberthur coll.); individualCount: 1; sex: male; **Taxon:** scientificName: Platylomia insignis Distant, 1912; **Location:** continent: Asia; country: Bhutan; locality: Maria-Basti; **Event:** eventDate: ??/??/1898; **Record Level:** institutionCode: NHMUK; basisOfRecord: PreservedSpecimen

##### Distribution

[Metcalf, 1963] Bhutan; China.

##### Notes

Authority: Distant 1912d

#### Platylomia larus

(Walker, 1858)

Dundubia larus Walker, 1858

##### Materials

- **Type status:** Holotype. **Occurrence:** catalogNumber: BMNH(E) 1009477; recordedBy: R. Templeton; sex: female; **Taxon:** scientificName: Platylomia larus (Walker, 1858); **Location:** continent: Asia; country: Sri Lanka; **Record Level:** institutionCode: NHMUK; basisOfRecord: PreservedSpecimen
- **Type status:** Other material. **Occurrence:** recordedBy: Leith; individualCount: 1; **Taxon:** scientificName: Platylomia larus (Walker, 1858); **Location:** continent: Asia; country: India; locality: Bombay; **Record Level:** basisOfRecord: PreservedSpecimen
- **Type status:** Other material. **Occurrence:** recordedBy: G. Hampson; individualCount: 1; **Taxon:** scientificName: Platylomia larus (Walker, 1858); **Location:** continent: Asia; country: India; locality: Neelgiri Hills, Koonoor; verbatimElevation: 600 ft; **Record Level:** basisOfRecord: PreservedSpecimen
- **Type status:** Other material. **Occurrence:** recordedBy: R. Templeton; individualCount: 1; **Taxon:** scientificName: Platylomia larus (Walker, 1858); **Location:** continent: Asia; country: Sri Lanka; locality: Ceylon; **Record Level:** institutionCode: NHMUK; basisOfRecord: PreservedSpecimen

##### Distribution

[Distant, 1889/92] Continental India: Bombay; Neelgiri Hills (Koonoor, 6000 ft). Ceylon. [Metcalf, 1963] Ceylon; India; Eastern India; Bombay; Kiangsu. [Duffels and van der Laan, 1985] Ceylon; China. [Sanborn, 2014] China, Jiangsu, India, Sri Lanka.

##### Notes

Authority: Walker 1858b

#### Platylomia malickyi

Beuk, 1998

Platylomia malickyi Beuk, 1998

##### Materials

- **Type status:** Holotype. **Occurrence:** recordedBy: Chantaramongkol and Malicky; individualCount: 1; sex: male; **Taxon:** scientificName: Platylomia malickyi Beuk, 1998; **Location:** continent: Asia; country: Thailand; locality: Changmai Zoo Lichtfalle; verbatimElevation: 400 m; **Event:** eventDate: 24-30/04/1989; **Record Level:** institutionCode: IZUI; basisOfRecord: PreservedSpecimen
- **Type status:** Paratype. **Occurrence:** individualCount: 1; sex: female; **Taxon:** scientificName: Platylomia malickyi Beuk, 1998; **Location:** continent: Asia; country: Myanmar; locality: Maymyo; **Event:** eventDate: 29/04/1901; **Record Level:** institutionCode: NHMUK; basisOfRecord: PreservedSpecimen
- **Type status:** Paratype. **Occurrence:** recordedBy: H.L. Andrewes; individualCount: 1; sex: female; **Taxon:** scientificName: Platylomia malickyi Beuk, 1998; **Location:** continent: Asia; country: Myanmar; locality: Maymyo; **Event:** eventDate: ??/05/1910; **Record Level:** institutionCode: NHMUK; basisOfRecord: PreservedSpecimen

##### Distribution

[Sanborn, 2014] Burma, Laos, Thailand, Vietnam, Southern China, Yunnan, China, Myanmar, Cambodia.

##### Notes

Authority: Beuk 1998; An additional 30 male paratypes (9 IZUI; 2 BPBM; 10 SUU; 1 MNHN; 4 NHMUK; 2 ZMAN; 1 USNM; 1 CASC) and 5 female paratypes (1 BPBM; 2 SUU; 2 NHMUK) were designated in the species description, however only the material from Myanmar is included here.

#### Platylomia operculata

Distant, 1913

Platylomia operculata Distant, 1913

##### Materials

- **Type status:** Lectotype. **Occurrence:** catalogNumber: BMNH(E) 1009486; recordedBy: R.V. de Salvaza; individualCount: 1; sex: male; **Taxon:** scientificName: Platylomia operculata Distant, 1913; **Location:** continent: Asia; locality: Indo-China; **Record Level:** institutionCode: NHMUK; basisOfRecord: PreservedSpecimen

##### Distribution

[Metcalf, 1963] Indochina. [Sanborn, 2014] Thailand, Burma, Laos, India, Indochina, Vietnam, China, Yunnan, Guangxi, Jiangxi, Hainan, Laos, Cambodia, Myanmar.

##### Notes

Authority: Distant 1913b; Female described by Boulard (2005). Sanborn (2014) listed India in reference to Boulard (2005). This species was described from Indo-China and there are currently no records of this species known from the Indian region, however it is present in the surrounding countries.

#### Platylomia radha

(Distant, 1881)

Dundubia radha Distant, 1881
Dundubia similis Distant, 1882

##### Materials

- **Type status:** Lectotype. **Occurrence:** individualCount: 1; sex: male; **Taxon:** scientificName: Platylomia radha (Distant, 1881); **Location:** continent: Asia; country: India; locality: Masuri Hills; **Record Level:** institutionCode: NHMUK; basisOfRecord: PreservedSpecimen
- **Type status:** Other material. **Occurrence:** catalogNumber: BMNH(E) 1009517; individualCount: 1; sex: female; **Taxon:** scientificName: Platylomia radha (Distant, 1881); **Location:** continent: Asia; country: India; locality: Sikkim; **Record Level:** institutionCode: NHMUK; basisOfRecord: PreservedSpecimen

##### Distribution

[Distant, 1889/92] Continental India: Madras Presidency; Naga Hills; Sikkim; Assam. Burma: Teinzo. [Metcalf, 1963] Madras; India; Burma; Siam; Thailand; Sikkim; Assam; Indochina; Bengal. [Duffels and van der Laan, 1985] Nepal. [Sanborn, 2014] India, China, Yunnan, Burma, Sikkim, Nepal, Thailand, Peninsular Malaysia, Assam, Bhutan, Nepal, Cambodia, Laos, Vietnam, China Hainan, Sichuan, yunnan, Indo-China, Peninsular Thailand, Taiwan, Guangxi, Jingxi, Hainan, Southeast Asia, Vietnam.

##### Notes

Authority: Distant 1881; Lectotype designated by Beuk (1998).

#### Platylomia vibrans

(Walker, 1850)

Dundubia vibrans Walker, 1850
Dundubia lateralis Walker, 1850

##### Materials

- **Type status:** Holotype. **Occurrence:** catalogNumber: BMNH(E) 1009481; sex: male; **Taxon:** scientificName: Platylomia vibrans (Walker, 1850); **Location:** continent: Asia; country: Bangladesh; locality: Silhet; **Record Level:** institutionCode: NHMUK; basisOfRecord: PreservedSpecimen

##### Distribution

[Distant, 1889/92] Continental India: Margherita (Assam); Naga Hills; Sylhet (Bangladesh). [Metcalf, 1963] India; Assam; East Bengal; Ceram; Malay Peninsula; Malacca.

##### Notes

Authority: Walker 1850

#### Platypleura affinis affinis

(Fabricius, 1803)

Tettigonia affinis Fabricius, 1803
Platypleura nicobarica Butler, 1877 (nec Atkinson, 1884)

##### Materials

- **Type status:** Holotype. **Occurrence:** individualCount: 1; **Taxon:** scientificName: Platypleura affinis affinis (Fabricius, 1803); **Location:** continent: Asia; country: India; locality: India Orientali; **Record Level:** institutionCode: MZLU; basisOfRecord: PreservedSpecimen
- **Type status:** Other material. **Occurrence:** catalogNumber: BMNH(E) 1009510; individualCount: 1; sex: male; **Taxon:** scientificName: Platypleura affinis affinis (Fabricius, 1803); **Location:** continent: Asia; country: India; locality: Nicobar Islands; **Record Level:** institutionCode: NHMUK; basisOfRecord: PreservedSpecimen

##### Distribution

[Metcalf, 1963] India; Cape of Good Hope (?); Eastern India; Nicobar Islands. [Sanborn, 2014] India Oriental.

##### Notes

Authority: Fabricius 1803

#### Platypleura affinis distincta

Atkinson, 1884

Platypleura affinis distincta Atkinson, 1884
Platypleura nicobarica Atkinson, 1884 (nec Butler, 1877)
Poecilopsaltria nicobarica var. a Distant, 1889

##### Materials

- **Type status:** Holotype. **Taxon:** scientificName: Platypleura affinis distincta Atkinson, 1884; **Location:** continent: Asia; country: India; locality: Nicobar Islands; **Record Level:** institutionCode: NZSI; basisOfRecord: PreservedSpecimen

##### Distribution

[Metcalf, 1963] Nicobar Islands.

##### Notes

Authority: Atkinson 1884

#### Platypleura andamana

Distant, 1878

Platypleura andamana Distant, 1878
Platypleura roepstorffii Atkinson, 1884

##### Materials

- **Type status:** Holotype. **Occurrence:** catalogNumber: BMNH(E) 1009513; individualCount: 1; sex: female; **Taxon:** scientificName: Platypleura andamana Distant, 1878; **Location:** continent: Asia; country: India; locality: Andaman Islands; **Record Level:** institutionCode: NHMUK; basisOfRecord: PreservedSpecimen

##### Distribution

[Metcalf, 1963] Andaman Islands; India. [Sanborn, 2014] Andaman Islands.

##### Notes

Authority: Distant 1878b

#### Platypleura assamensis

Atkinson, 1884

Platypleura assamensis Atkinson, 1884
Platypleura repanda var. a Distant, 1889

##### Materials

- **Type status:** Syntype. **Taxon:** scientificName: Platypleura assamensis Atkinson, 1884; **Location:** continent: Asia; country: India; locality: Sibsagar; **Record Level:** institutionCode: NZSI; basisOfRecord: PreservedSpecimen
- **Type status:** Other material. **Occurrence:** catalogNumber: BMNH(E) 1009507; recordedBy: William Doherty; individualCount: 1; sex: female; **Taxon:** scientificName: Platypleura assamensis Atkinson, 1884; **Location:** continent: Asia; country: India; locality: Margherita, Upper Assam; **Record Level:** institutionCode: NHMUK; basisOfRecord: PreservedSpecimen
- **Type status:** Syntype. **Taxon:** scientificName: Platypleura assamensis Atkinson, 1884; **Location:** continent: Asia; country: India; locality: Naga Hills; **Record Level:** institutionCode: NZSI; basisOfRecord: PreservedSpecimen

##### Distribution

[Distant, 1889/92] Continental India: Daejeeling; Assam: Seebsager and Naga Hills; Khasi Hills .; [Metcalf, 1963] Assam; Sibsagar; India; Uttar Pradesh. [Duffels and van der Laan, 1985] Bhutan. [Sanborn, 2014] China.

##### Notes

Authority: Atkinson 1884

#### Platypleura badia

Distant, 1888

Platypleura badia Distant, 1888
Platypleura fasuayensis Boulard, 2005

##### Materials

- **Type status:** Holotype. **Occurrence:** recordedBy: Leonardo Fea; sex: female; **Taxon:** scientificName: Platypleura badia Distant, 1888; **Location:** continent: Asia; country: Myanmar; locality: Houngdarau Valley, Tenasserim; **Record Level:** institutionCode: MSNG; basisOfRecord: PreservedSpecimen
- **Type status:** Other material. **Occurrence:** catalogNumber: BMNH(E) 1009506; occurrenceRemarks: Specimen not part of type series, see taxon notes.; recordedBy: G.C. Clarence; individualCount: 1; sex: male; **Taxon:** scientificName: Platypleura badia Distant, 1888; **Location:** continent: Asia; country: Myanmar; locality: Toungoo Dist., Lower Burma; **Event:** eventDate: ??/??/1913; **Record Level:** institutionCode: NHMUK; basisOfRecord: PreservedSpecimen

##### Distribution

[Metcalf, 1963] Tenasserim; Lower Burma; Indo-China; Perak; Burma; Tonkin; Malay Penninsula. [Sanborn, 2014] China, Vietnam, Thailand, India, Burma, Vietnam, Tenasserim, Burma, North Vietnam, Yunnan, Malaysia, Myanmar.

##### Notes

Authority: Distant 1888c; In the NHMUK there is a male specimen bearing a type label, however this male was described later by Distant (1913b). Holotype is a female from "Tenasserim: Houngdarau Valley" (MSNG). Not from India: Sanborn (2014) states India in reference to Sanborn et al. (2007) and Lee (2008), however their inclusion of India is in reference to previous distributions that may be incorrect, having resulted from Indo-China being included as India. Further evidence is required before this species can be included in the fauna of this region.

#### Platypleura basialba

(Walker, 1850)

Oxypleura basialba Walker, 1850

##### Materials

- **Type status:** Holotype. **Occurrence:** catalogNumber: BMNH(E) 1009491; individualCount: 1; sex: male; **Taxon:** scientificName: Platypleura basialba (Walker, 1850); **Location:** continent: Asia; country: India; locality: N. Bengal; **Record Level:** institutionCode: NHMUK; basisOfRecord: PreservedSpecimen
- **Type status:** Other material. **Occurrence:** catalogNumber: BMNH(E) 1009490; individualCount: 1; sex: female; **Taxon:** scientificName: Platypleura basialba (Walker, 1850); **Location:** locality: Locality unknown; **Event:** eventDate: ??/04/1919; **Record Level:** institutionCode: NHMUK; basisOfRecord: PreservedSpecimen

##### Distribution

[Metcalf, 1963] Bengal; India; Northern Bengal; British India; Uttar Pradesh. [Sanborn, 2014] Pakistan, India.

##### Notes

Authority: Walker 1850

#### Platypleura basiviridis

Walker, 1850

Platypleura basiviridis Walker, 1850

##### Materials

- **Type status:** Holotype. **Occurrence:** catalogNumber: BMNH(E) 1009373; individualCount: 1; sex: male; **Taxon:** scientificName: Platypleura basiviridis Walker, 1850; **Location:** locality: Locality unknown; **Record Level:** institutionCode: NHMUK; basisOfRecord: PreservedSpecimen

##### Distribution

[Distant, 1889/92] Continental India: Karwar. [Metcalf, 1963] India; Bombay; Madras; Mysore; Coorg. [Sanborn, 2014] India.

##### Notes

Authority: Walker 1850

#### Platypleura bufo

(Walker, 1850)

Oxypleura bufo Walker, 1850

##### Materials

- **Type status:** Holotype. **Occurrence:** catalogNumber: BMNH(E) 1009502; recordedBy: R.H. Inglis; individualCount: 1; sex: male; **Taxon:** scientificName: Platypleura bufo (Walker, 1850); **Location:** continent: Asia; country: India; locality: E. India; **Record Level:** institutionCode: NHMUK; basisOfRecord: PreservedSpecimen

##### Distribution

[Metcalf, 1963] India; Eastern India; British India.

##### Notes

Authority: Walker 1850

#### Platypleura capitata

(Olivier, 1790)

Cicada capitata Olivier, 1790
Oxypleura subrufa Walker, 1850

##### Materials

- **Type status:** Holotype. **Taxon:** scientificName: Platypleura capitata (Olivier, 1790); **Location:** continent: Asia; country: Sri Lanka; **Record Level:** basisOfRecord: PreservedSpecimen
- **Type status:** Other material. **Occurrence:** catalogNumber: BMNH(E) 1009492; recordedBy: H.A. Latham; individualCount: 1; sex: male; **Taxon:** scientificName: Platypleura capitata (Olivier, 1790); **Location:** continent: Asia; country: India; locality: Chitleri Hills, Salem District, Madras; **Record Level:** institutionCode: NHMUK; basisOfRecord: PreservedSpecimen

##### Distribution

[Metcalf, 1963] Ceylon; Java; India; Hyderabad; Madras; Central Provinces; United Provinces; Japan; Madhya Pradesh; Uttar Pradesh. [Duffels and van der Laan, 1985] Ceylon. [Sanborn, 2014] Sri Lanka, India.

##### Notes

Authority: Olivier 1790

#### Platypleura cervina

Walker, 1850

Platypleura cervina Walker, 1850
Platypleura straminea Walker, 1850

##### Materials

- **Type status:** Holotype. **Occurrence:** catalogNumber: BMNH(E) 1009500; individualCount: 1; sex: female; **Taxon:** scientificName: Platypleura cervina Walker, 1850; **Location:** continent: Asia; country: India; locality: N. Bengal; **Record Level:** institutionCode: NHMUK; basisOfRecord: PreservedSpecimen

##### Distribution

[Metcalf, 1963] Bengal; Northern India; India; Northern Bengal.

##### Notes

Authority: Walker 1850

#### Platypleura coelebs

Stål, 1863

Platypleura coelebs Stål, 1863

##### Materials

- **Type status:** Holotype. **Occurrence:** catalogNumber: BMNH(E) 1009508; individualCount: 1; sex: male; **Taxon:** scientificName: Platypleura coelebs Stål, 1863; **Location:** continent: Asia; country: India; locality: E. India; **Record Level:** institutionCode: NHMUK; basisOfRecord: PreservedSpecimen

##### Distribution

[Distant, 1889/92] India: Dekhan. [Metcaf, 1963] Eastern India; Northern India; India; China; Deccan; Central China; Oriental Region; Chusan Archipelago; Tonkin; Chusan Islands. [Sanborn, 2014] China, Chusan, Guangdong, Guangxi, Zhejiang, Vietnam, India.

##### Notes

Authority: Stål 1863

#### Platypleura hampsoni

(Distant, 1887)

Poecilopsaltria hampsoni Distant, 1887

##### Materials

- **Type status:** Holotype. **Occurrence:** catalogNumber: BMNH(E) 1009374; recordedBy: G.F. Hampson; individualCount: 1; sex: female; **Taxon:** scientificName: Platypleura hampsoni (Distant, 1887); **Location:** continent: Asia; country: India; locality: Nilgiri Hills, northern slopes; verbatimElevation: 5000 ft; **Event:** eventDate: 29/05/1887; **Record Level:** institutionCode: NHMUK; basisOfRecord: PreservedSpecimen

##### Distribution

[Distant, 1889/92] India: Neelgiri Hills, n. slopes, 3500 and 5000 ft. [Metcalf, 1963] Madras; India. [Sanborn, 2014] India.

##### Notes

Authority: Distant 1887a

#### Platypleura inglisi

Ollenbach, 1929

Platypleura inglisi Ollenbach, 1929

##### Materials

- **Type status:** Holotype. **Occurrence:** recordedBy: H. Inglis; individualCount: 1; sex: female; **Taxon:** scientificName: Platypleura inglisi Ollenbach, 1929; **Location:** continent: Asia; country: India; locality: Darjeeling, Kurseong; verbatimElevation: 4000 ft; **Record Level:** institutionCode: IFRI; basisOfRecord: PreservedSpecimen

##### Distribution

[Metcalf, 1963] Bengal; India.

##### Notes

Authority: Ollenbach 1929

#### Platypleura insignis

Distant, 1879

Platypleura insignis Distant, 1879

##### Materials

- **Type status:** Holotype. **Occurrence:** recordedBy: Limborg; individualCount: 1; sex: male; **Taxon:** scientificName: Platypleura insignis Distant, 1879; **Location:** continent: Asia; country: Myanmar; locality: Upper Tenasserim; **Record Level:** institutionCode: NZSI; basisOfRecord: PreservedSpecimen
- **Type status:** Other material. **Occurrence:** catalogNumber: BMNH(E) 1009505; recordedBy: William Doherty; individualCount: 1; sex: female; **Taxon:** scientificName: Platypleura insignis Distant, 1879; **Location:** continent: Asia; country: Myanmar; locality: Karen Hills; **Record Level:** institutionCode: NHMUK; basisOfRecord: PreservedSpecimen

##### Distribution

[Distant, 1906] India: Upper Tenasserim. Burma: Karen Hills. [Metcalf, 1963] Tenasserim; Hindustan; Burma; India. [Sanborn, 2014] Indochina, Thailand, Tenasserim.

##### Notes

Authority: Distant 1879; Metcalf (1963) lists "India", however the type was collected from Upper Tenasserim (southern Myanmar), which was formerly a part of "British India" but is quite distant from the India/Myanmar border. No records have been found in modern India at present.

#### Platypleura intermedia

Liu, 1940

Platypleura intermedia Liu, 1940

##### Materials

- **Type status:** Holotype. **Occurrence:** individualCount: 1; sex: male; **Taxon:** scientificName: Platypleura intermedia Liu, 1940; **Location:** continent: Asia; country: Sri Lanka; locality: Poffua, Ceylon; **Record Level:** institutionCode: MCZ; basisOfRecord: PreservedSpecimen

##### Distribution

[Liu, 1940] Ceylon: Poffua.

##### Notes

Authority: Liu 1940

#### Platypleura mackinnoni

Distant, 1904

Platypleura mackinnoni Distant, 1904

##### Materials

- **Type status:** Syntype. **Occurrence:** catalogNumber: BMNH(E) 1009503; recordedBy: P.W. Mackinnon; individualCount: 1; sex: male; **Taxon:** scientificName: Platypleura mackinnoni Distant, 1904; **Location:** continent: Asia; country: India; locality: Dehra Dun, Mussooree; **Event:** eventDate: 18/06/1903; **Record Level:** institutionCode: NHMUK; basisOfRecord: PreservedSpecimen
- **Type status:** Syntype. **Occurrence:** catalogNumber: NHMUK 010214248; recordedBy: P.W. Mackinnon; individualCount: 1; sex: female; **Taxon:** scientificName: Platypleura mackinnoni Distant, 1904; **Location:** continent: Asia; country: India; locality: Dehra Dun, Mussooree; **Event:** eventDate: 1259; **Record Level:** institutionCode: NHMUK; basisOfRecord: PreservedSpecimen

##### Distribution

[Metcalf, 1963] Mysore; India; Bihar; Uttar Pradesh; Northern India. [Duffels and van der Laan, 1985] India. [Sanborn, 2014] Nepal, Pakistan, India.

##### Notes

Authority: Distant 1904b

#### Platypleura nobilis nobilis

(Germar, 1830)

Cicada nobilis Germar, 1830
Cicada hemiptera Guérin-Méneville, 1843
Platypleura gemina Walker, 1850
Platypleura semilucida Walker, 1850

##### Materials

- **Type status:** Holotype. **Taxon:** scientificName: Platypleura nobilis nobilis (Germar, 1830); **Location:** continent: Asia; country: Indonesia; locality: Java; **Record Level:** basisOfRecord: PreservedSpecimen

##### Distribution

[Distant, 1906] India: Garo Hills; Samagooting; Munjpoor. Burma: Tavoy. [Metcalf, 1963] Java; Sumatra; Malay Peninsula; Borneo; Hindustan; Tenasserim; Singapore Island; India; Assam; Burma; Sarawak; Malaya; Malacea; Perak; Penang; Johore; Sarawak (?); Siam; Manipur. [Duffels and van der Laan, 1985] Andaman Islands; Nicobar Islands; Philippines. [Sanborn, 2014] China, Java, Malaysia, Peninsular Malaysia, Borneo, Sabah, Sarawak, Thailand, Philippine Republic, Indochina, Java, Sumatra, Assam, India, Burma, Siam.

##### Notes

Authority: Germar 1830

#### Platypleura octoguttata octoguttata

(Fabricius, 1798)

Tettigonia octoguttata Fabricius, 1798
Oxypleura sanguiflua Walker, 1850

##### Materials

- **Type status:** Holotype. **Taxon:** scientificName: Platypleura octoguttata octoguttata (Fabricius, 1798); **Location:** continent: South America; country: French Guiana; locality: Cajennae; **Record Level:** basisOfRecord: PreservedSpecimen
- **Type status:** Other material. **Occurrence:** catalogNumber: BMNH(E) 1009482; recordedBy: A. Berritt; individualCount: 1; sex: female; **Taxon:** scientificName: Platypleura octoguttata octoguttata (Fabricius, 1798); **Location:** continent: Asia; country: India; locality: Jubbulpore; **Event:** eventDate: ??/??/1936; **Record Level:** institutionCode: NHMUK; basisOfRecord: PreservedSpecimen

##### Distribution

[Distant, 1889/92] India: Panjab (Wazeerabad); North Bengal; Naini Tal; Rajpootana (Mount Aboo); Jodhpoor; Sambalpoor; Karachi; Bombay; Karwar; Coimbatore; Neelgiri Hills (Southern Slopes); Shivarai Hills.; [Metcalf, 1963] French Guiana [?]; India; Bengal; Eastern India; Surinam [?]; Punjab; Northern Bengal; Northern India; Southern India; Calcutta; Central Provinces; Bombay; Ceylon; Mysore; Madras; Central India; Rajpatana; United Provinces; Uttar Pradesh. [Duffels and van der Laan, 1985] Ceylon; India. [Sanborn, 2014] India, Thailand, Pakistan.

##### Notes

Authority: Fabricius 1798; The type locality was stated as "Cajennae" [French Guiana], however all other specimens have been collected from the Indian region.

#### Platypleura polita polita

(Walker, 1850)

Oxypleura polita Walker, 1850

##### Materials

- **Type status:** Holotype. **Occurrence:** catalogNumber: BMNH(E) 1009504; individualCount: 1; sex: female; **Taxon:** scientificName: Platypleura polita polita (Walker, 1850); **Location:** locality: Locality unknown; **Record Level:** institutionCode: NHMUK; basisOfRecord: PreservedSpecimen

##### Distribution

[Distant, 1889/92] Continental India: Karwar, Canara, Trevandrum. [Metcalf, 1963] India; Bombay; British India; Travancore; Madras. [Sanborn, 2014] India.

##### Notes

Authority: Walker 1850

#### Platypleura politavar.a

(Distant, 1889)

Poecilopsaltria polita var. a Distant, 1889

##### Materials

- **Type status:** Holotype. **Taxon:** scientificName: Platypleura polita var. a (Distant, 1889); **Location:** locality: Locality unknown; **Record Level:** basisOfRecord: PreservedSpecimen

##### Distribution

[Distant, 1889/92] Continental India: Karwar, Canara, Trevandrum. [Metcalf, 1963] India; Bombay; British India; Travancore; Madras.

##### Notes

Authority: Distant 1889a

#### Platypleura sphinx

Walker, 1850

Platypleura sphinx Walker, 1850

##### Materials

- **Type status:** Syntype. **Occurrence:** catalogNumber: BMNH(E) 1009371; individualCount: 1; sex: male; **Taxon:** scientificName: Platypleura sphinx Walker, 1850; **Location:** continent: Asia; country: India; locality: N. India; **Record Level:** institutionCode: NHMUK; basisOfRecord: PreservedSpecimen
- **Type status:** Syntype. **Occurrence:** catalogNumber: NHMUK 010214247; individualCount: 1; sex: female; **Taxon:** scientificName: Platypleura sphinx Walker, 1850; **Location:** continent: Asia; country: India; locality: North Bengal; **Record Level:** institutionCode: NHMUK; basisOfRecord: PreservedSpecimen

##### Distribution

[Distant, 1906] India: North Bengal, Mhow. [Metcalf, 1963] Northern Bengal; Northern India; India; Bengal.

##### Notes

Authority: Walker 1850

#### Platypleura watsoni

(Distant, 1897)

Poecilopsaltria watsoni Distant, 1897
Platypleura mokensis Boulard, 2003

##### Materials

- **Type status:** Holotype. **Occurrence:** catalogNumber: BMNH(E) 1009372; recordedBy: Watson; individualCount: 1; sex: female; **Taxon:** scientificName: Platypleura watsoni (Distant, 1897); **Location:** continent: Asia; country: Myanmar; locality: North Chin Hills; **Record Level:** institutionCode: NHMUK; basisOfRecord: PreservedSpecimen

##### Distribution

[Metcalf, 1963] Burma; Tenasserim. [Sanborn, 2014] Thailand, India, Myanmar, Siam.

##### Notes

Authority: Distant 1897; Sanborn (2014) lists India in reference to Sanborn et al. (2007) whose distribution list was generated from Metcalf (1963), which only states Burma and Tenasserim. The type locality (Chin Hills) extends into India (Lushai Hills - Nagaland), thus this species may be recorded in Nagaland in future.

#### Platypleura westwoodi

Stål, 1863

Platypleura westwoodi Stål, 1863

##### Materials

- **Type status:** Holotype. **Occurrence:** individualCount: 1; sex: female; **Taxon:** scientificName: Platypleura westwoodi Stål, 1863; **Location:** continent: Asia; country: Sri Lanka; **Record Level:** institutionCode: NHRS; basisOfRecord: PreservedSpecimen
- **Type status:** Other material. **Occurrence:** catalogNumber: BMNH(E) 1009512; individualCount: 1; sex: male; **Taxon:** scientificName: Platypleura westwoodi Stål, 1863; **Location:** continent: Asia; country: Sri Lanka; **Record Level:** institutionCode: NHMUK; basisOfRecord: PreservedSpecimen

##### Distribution

[Metcalf, 1963] Ceylon. [Duffels and van der Laan, 1985] Ceylon.

##### Notes

Authority: Stål 1863

#### Polyneura ducalis

Westwood, 1840

Polyneura ducalis Westwood, 1840

##### Materials

- **Type status:** Holotype. **Occurrence:** recordedBy: Hardwicke; **Taxon:** scientificName: Polyneura ducalis Westwood, 1840; **Location:** continent: Asia; country: India; **Record Level:** basisOfRecord: PreservedSpecimen
- **Type status:** Other material. **Occurrence:** recordedBy: Jones; individualCount: 1; sex: male; **Taxon:** scientificName: Polyneura ducalis Westwood, 1840; **Location:** continent: Asia; country: India; locality: Simla; **Event:** eventDate: ??/??/1930; **Record Level:** institutionCode: NHMUK; basisOfRecord: PreservedSpecimen

##### Distribution

[Distant, 1889/92 and Distant, 1906] Continental India: Nepal; North-Western Province, Mussooree; Ranikhet; Sikkim and Assam; Darjeeling. Burma: Rangoon, Se-Tchouen; Tibet.; [Metcalf, 1963] India; Nepal; East Indies; Sikkim; Assam; Burma; Mysore; United Provinces; Szechwan; China; Tibet; Oriental Region; North Western Province; Cochin China; Central China; Sikang; Uttar Pradesh. [Duffels and van der Laan, 1985] China; Nepal. [Sanborn, 2014] India, China, Szechuan, Tibet, Assam, Sikhim, Hunan, Guangxi, Sichuan, Yunnan, Xizang, Indonesia, Burma, Nepal, South Vietnam,, Tibet, Myanmar.

##### Notes

Authority: Westwood 1840

#### Polyneura laevigata

Chou & Yao, 1986

Polyneura laevigata Chou & Yao, 1986

##### Materials

- **Type status:** Holotype. **Occurrence:** individualCount: 1; sex: male; **Taxon:** scientificName: Polyneura laevigata Chou & Yao, 1986; **Location:** continent: Asia; country: China; **Event:** eventDate: ??/09/1958; **Record Level:** basisOfRecord: PreservedSpecimen

##### Distribution

[Sanborn, 2014] China, Jiangxi, Nepal.

##### Notes

Authority: Chou and Yao 1986

#### Pomponia cinctimanus

(Walker, 1850)

Dundubia cinctimanus Walker, 1850

##### Materials

- **Type status:** Holotype. **Occurrence:** catalogNumber: BMNH(E) 1009389; sex: male; **Taxon:** scientificName: Pomponia cinctimanus (Walker, 1850); **Location:** continent: Asia; country: Bangladesh; locality: Silhet; **Record Level:** institutionCode: NHMUK; basisOfRecord: PreservedSpecimen

##### Distribution

[Metcalf, 1963] Assam; Silhet. [Sanborn, 2014] Sylhet, Vietnam.

##### Notes

Authority: Walker 1850

#### Pomponia cyanea

Fraser, 1948

Pomponia cyanea Fraser, 1948

##### Materials

- **Type status:** Syntype. **Occurrence:** catalogNumber: BMNH(E) 1009394; recordedBy: F.C. Fraser; individualCount: 1; sex: male; **Taxon:** scientificName: Pomponia cyanea Fraser, 1948; **Location:** continent: Asia; country: India; locality: Munnar, Travancore, Eastern Outlet; verbatimElevation: 4000 ft; **Event:** eventDate: ??/05/??; **Record Level:** institutionCode: NHMUK; basisOfRecord: PreservedSpecimen
- **Type status:** Syntype. **Occurrence:** catalogNumber: BMNH(E) 1009395; recordedBy: F.C. Fraser; individualCount: 1; sex: male; **Taxon:** scientificName: Pomponia cyanea Fraser, 1948; **Location:** continent: Asia; country: India; locality: Munnar, Travancore; **Event:** eventDate: ??/??/1933; **Record Level:** institutionCode: NHMUK; basisOfRecord: PreservedSpecimen
- **Type status:** Syntype. **Occurrence:** catalogNumber: BMNH(E) 1009396; recordedBy: F.C. Fraser; individualCount: 1; sex: male; **Taxon:** scientificName: Pomponia cyanea Fraser, 1948; **Location:** continent: Asia; country: India; locality: Munnar, Travancore; **Event:** eventDate: ??/??/1933; **Record Level:** institutionCode: NHMUK; basisOfRecord: PreservedSpecimen
- **Type status:** Syntype. **Occurrence:** catalogNumber: BMNH(E) 1009397; recordedBy: F.C. Fraser; individualCount: 1; sex: male; **Taxon:** scientificName: Pomponia cyanea Fraser, 1948; **Location:** continent: Asia; country: India; locality: Munnar, Travancore; **Event:** eventDate: ??/??/1933; **Record Level:** institutionCode: NHMUK; basisOfRecord: PreservedSpecimen
- **Type status:** Syntype. **Occurrence:** catalogNumber: BMNH(E) 1009398; individualCount: 1; sex: male; **Taxon:** scientificName: Pomponia cyanea Fraser, 1948; **Location:** continent: Asia; country: India; locality: Munnar, Travancore; **Record Level:** institutionCode: NHMUK; basisOfRecord: PreservedSpecimen

##### Distribution

[Metcalf, 1963] Coorg; Travancore. [Sanborn, 2014] India.

##### Notes

Authority: Fraser 1948

#### Pomponia linearis

(Walker, 1850)

Dundubia linearis Walker, 1850

##### Materials

- **Type status:** Holotype. **Occurrence:** catalogNumber: BMNH(E) 1009401; sex: male; **Taxon:** scientificName: Pomponia linearis (Walker, 1850); **Location:** locality: Locality unknown; **Record Level:** institutionCode: NHMUK; basisOfRecord: PreservedSpecimen
- **Type status:** Other material. **Occurrence:** individualCount: 1; sex: female; **Taxon:** scientificName: Pomponia linearis (Walker, 1850); **Location:** continent: Asia; country: India; locality: Tenmalai, Travancore, S. India; verbatimElevation: 500 - 800 ft; **Event:** eventDate: 11-17/10/1938; **Record Level:** institutionCode: NHMUK; basisOfRecord: PreservedSpecimen

##### Distribution

[Distant, 1889/92] Continental India: Margherita, Assam; Naga Hills; Seebsagar; Neelgiri Hills (north slopes); Sylhet and Cachar (Bangladesh). [Metcalf, 1963] Sumatra; Bengal; Assam; India; Silhet; Java; Philippine Islands; Aru Islands; Hindustan; Malay Peninsula; Japan; Malacca; Tonkin; Malay Archipelago; Palaearctic Japan; Formosa; Ryukyu Islands; China; Singapore Island; Perak; Johore; Penang; British India; Sunda Islands; Indochina; Kyushu (?); Kwangtung; East Indies; Siam; Selangor; New Guinea; East Indies (?); Eastern India; Japan (?); Loochoo Islands; Sarawak; Ishigaki Island; Kwangsi; Jehol; Naga Hills; Madras; Yaeyama Islands; Chekiang; Fukien; Sikang; Burma; Uttar Pradesh; Borneo; Malaya. [Duffels and van der Laan, 1985] Taiwan; Ryukyu Islands; Japan; China; Malaysia; India; Yaeyama Islands (Japan); Nepal; Thailand. [Sanborn, 2014] Japan, Malaysia, China, Hunan, Taiwan, Indonesia, Kratatau, Borneo, Sabah, Peninsular Malaysia, Indo-China, New Guinea, Java, Sarawak, Shaanxi, Anhui, Jiangxi, Zhejiang, Fujian, Taiwan, Guangdong, Guangxi, Sichuan, Yunnan, Xizang, Philippines, Indonesia, Malaysia, India, Thailand, Malay Peninsula, Borneo, Taiwan, Vietnam, Laos, Cambodia, Singapore, Indonesia, Myanmar, Bangladesh, Nepal, Manipur, Assam, Margherita, Naga Hills, Nilgiri Hills, Sibsagar, Sylhet, Philippines, Bhutan, Philippine Republic, Southeast Asia, Laos, Bangladesh.

##### Notes

Authority: Walker 1850

#### Pomponia picta

(Walker, 1870)

Dundubia picta Walker, 1870
Cicada fusca Olivier, 1790 (nec Müller, 1776)
Pomponia fusca Boulard, 2001 (nec Olivier, 1790)

##### Materials

- **Type status:** Holotype. **Occurrence:** sex: female; **Taxon:** scientificName: Pomponia picta (Walker, 1870); **Location:** continent: Asia; country: Indonesia; locality: Sumatra; **Record Level:** institutionCode: NHMUK; basisOfRecord: PreservedSpecimen

##### Distribution

[Distant, 1916] Ceylon: Pattipola [Metcalf, 1963] Sumatra; Borneo; Java; Sarawak; Ceylon; Malay Peninsula; New Guinea. [Duffels and van der Laan, 1985] Ceylon. [Sanborn, 2014] China, Han-lik, Canton, Kwangsi, Sylhet, Assam, Margherita, Naga Hills, Sibsagar, Cachar, Nilgiri Hils, Malay Peninsula, Java, Philippines, Japan, Malaysia, Borneo, Sabah, Borneo, Sarawak, Sumatra, Java, New Guinea, Taiwan, Indonesia, Sri Lanka, Sumatra, Nias Island, Thailand, Philippine Republic, Malay Archipelago, Japan, Malacca, Indochina, Vietnam, Nepal.

##### Notes

Authority: Walker 1870

#### Pomponia ramifera

(Walker, 1850)

Dundubia ramifera Walker, 1850

##### Materials

- **Type status:** Holotype. **Occurrence:** catalogNumber: BMNH(E) 1009390; sex: male; **Taxon:** scientificName: Pomponia ramifera (Walker, 1850); **Location:** continent: Asia; country: Bangladesh; locality: Silhet; **Record Level:** institutionCode: NHMUK; basisOfRecord: PreservedSpecimen

##### Distribution

[Metcalf, 1963] Assam; Silhet. [Sanborn, 2014] Sylhet, Vietnam.

##### Notes

Authority: Walker 1850

#### Pomponia secreta

Hayashi, 1978

Pomponia secreta Hayashi, 1978

##### Materials

- **Type status:** Holotype. **Occurrence:** recordedBy: T. Aoki and S. Yamaguchi; individualCount: 1; sex: male; **Taxon:** scientificName: Pomponia secreta Hayashi, 1978; **Location:** continent: Asia; country: Nepal; locality: Tatopani, C. Nepal; verbatimElevation: 1200 m; **Event:** eventDate: 11/05/1974; **Record Level:** institutionCode: NSMT; basisOfRecord: PreservedSpecimen

##### Distribution

[Duffels and van der Laan, 1985] Nepal.

##### Notes

Authority: Hayashi 1978

#### Pomponia solitaria

Distant, 1888

Pomponia solitaria Distant, 1888

##### Materials

- **Type status:** Holotype. **Occurrence:** catalogNumber: BMNH(E) 1009404; individualCount: 1; sex: male; **Taxon:** scientificName: Pomponia solitaria Distant, 1888; **Location:** continent: Asia; country: India; locality: Narkondam, Andaman Islands; **Record Level:** institutionCode: NHMUK; basisOfRecord: PreservedSpecimen

##### Distribution

[Metcalf, 1963] Andaman Islands; Narkondam Island. [Duffels and van der Laan, 1985] Andaman Islands; Nicobar Islands.

##### Notes

Authority: Distant 1888b

#### Pomponia surya

Distant, 1904

Pomponia surya Distant, 1904

##### Materials

- **Type status:** Syntype. **Occurrence:** catalogNumber: BMNH(E) 1009405; recordedBy: P.W. Mackinnon; sex: male; **Taxon:** scientificName: Pomponia surya Distant, 1904; **Location:** continent: Asia; country: India; locality: Mussoorie; verbatimElevation: 5000 ft; **Event:** eventDate: 30/05/1903; **Record Level:** institutionCode: NHMUK; basisOfRecord: PreservedSpecimen
- **Type status:** Syntype. **Occurrence:** recordedBy: P.W. Mackinnon; sex: female; **Taxon:** scientificName: Pomponia surya Distant, 1904; **Location:** continent: Asia; country: India; locality: Mussoorie (Masuri); verbatimElevation: 5000 ft; **Event:** eventDate: 30/05/1903; **Record Level:** basisOfRecord: PreservedSpecimen

##### Distribution

[Metcalf, 1963] Mysore; India; Uttar Pradesh. [Duffels and van der Laan, 1985] Bhutan.

##### Notes

Authority: Distant 1904b

#### Pomponia urania

(Walker, 1850)

Dundubia urania Walker, 1850

##### Materials

- **Type status:** Holotype. **Occurrence:** catalogNumber: BMNH(E) 1009393; sex: male; **Taxon:** scientificName: Pomponia urania (Walker, 1850); **Location:** continent: Asia; country: India; locality: E. India; **Record Level:** institutionCode: NHMUK; basisOfRecord: PreservedSpecimen

##### Distribution

[Metcalf, 1963] India. [Sanborn, 2014] East India?, Vietnam.

##### Notes

Authority: Walker 1850

#### Pomponia zebra

Bliven, 1964

Pomponia zebra Bliven, 1964

##### Materials

- **Type status:** Holotype. **Occurrence:** recordedBy: P. Susai Nathan; individualCount: 1; sex: male; **Taxon:** scientificName: Pomponia zebra Bliven, 1964; **Location:** continent: Asia; country: India; locality: Kadamparai, Anamalai Hills; verbatimElevation: 3500 ft; **Event:** eventDate: ??/05/1963; **Record Level:** institutionCode: CAS; basisOfRecord: PreservedSpecimen

##### Distribution

[Duffels and van der Laan, 1985] Southern India.

##### Notes

Authority: Bliven 1964

#### Proarna hilaris

(Germar, 1834)

Cicada hilaris Germar, 1834
Cicada subtincta Walker, 1850
Cicada albiflos Walker, 1850
Cicada tomentosa Walker, 1858

##### Materials

- **Type status:** Holotype. **Occurrence:** recordedBy: Hope; **Taxon:** scientificName: Proarna hilaris (Germar, 1834); **Location:** continent: Oceania; locality: Australasia; **Record Level:** basisOfRecord: PreservedSpecimen

##### Distribution

[Metcalf, 1963] Australia [?]; Assam [?]; Cuba; Saint Thomas Island; Jamaica; East Bengal [?]; Santo Domingo; Philippine Islands [?]; Molucca Islands [?]; Mexico; Antilles; Dominica; South America; Saint Croix Island; Porto Rico; West Indies. [Sanborn, 2014] Puerto Rico, Dominican Republic, French Antilles, Guadeloupe, Martinique, Guana Island, British West Indies, Jamaica, Mexico, Caribbean Islands, Venezuela, Hispaniola.

##### Notes

Authority: Germar 1834; A junior synonym (*Cicada subtincta* Walker, 1850) of this species was described from Silhet (Bangladesh). It should be noted that the other two junior synonyms (Cicada albiflos Walker, 1850 and Cicada tomentosa Walker, 1858) were described from Jamaica and West Indies, while the senior synonym, *Proarna hilaris* (Germar, 1834) was described from Australasia. Thus if the type locality of the junior synonym (*Cicada subtincta*) is correct it may have been incorrectly synonymised with Proarna hilaris by Stål (1862). Though not officially recorded from India it is highly likely that the junior synonym *C. subtincta* occurs in India.

#### Psalmocharias querula

(Pallas, 1773)

Cicada querula Pallas, 1773
Cephaloxys quadrimacula Walker, 1850
Cicada steveni Stal, 1854

##### Materials

- **Type status:** Holotype. **Taxon:** scientificName: Psalmocharias querula (Pallas, 1773); **Location:** continent: Asia; country: Russia; **Record Level:** basisOfRecord: PreservedSpecimen

##### Distribution

[Metcalf, 1963] Russia; Ural Mountains; Siberia; India; Southern Russia; Orenburg; Transcaucasia; Chkalov; Saratov; Southern Europe; Dagestan; St. Petersburg; Germany; Austria; Sweden; France; Northern India; Tunisia; Persia; Turkey; Turkestan; Afghanistan; Caucasus; Southern France; Algeria; Caspian Region; Quetta-Pishin; Palearctic Region; Northwestern India; Asiatic Russia; Syr Darya; Uzbek; Crimea; Russian Transcaspia; Baluchistan; Eastern Palearctic Region; Armenia; Tashkent; Palestine; Balistan; Iran. [Duffels and van der Laan, 1985] Himalaya; Europe; Afghanistan; Spain; Uzbekistan; U.S.S.R.; Israel; South-East U.S.S.R.; Egypt; Mongolia; Kazakhstan S.S.R.; South of France; Algeria; Iran; Tunisia. [Sanborn, 2014] Tajikistan, Iran, Afghanistan, Turkey, Algeria, France, Israel, Mongolia, Tunisia, USSR, Uzbekistan, Tadzhikistan, Ukraine, Russia, Spain, Kirghizia, Transcaucasia, Crimea, Egypt, Pakistan, India, China, Central Asia, Xinjiang, Turkestan, Europe, Mongoia.

##### Notes

Authority: Pallas 1773

#### Psalmocharias rugipennis

(Walker, 1858)

Cicada rugipennis Walker, 1858
Sena querula var. ______ Distant, 1906

##### Materials

- **Type status:** Holotype. **Occurrence:** catalogNumber: BMNH(E) 1009574; individualCount: 1; sex: female; **Taxon:** scientificName: Psalmocharias rugipennis (Walker, 1858); **Location:** continent: Asia; country: India; locality: Hindostan; **Record Level:** institutionCode: NHMUK; basisOfRecord: PreservedSpecimen
- **Type status:** Other material. **Occurrence:** catalogNumber: BMNH(E) 1009575; recordedBy: W.H. Evans; individualCount: 1; sex: female; **Taxon:** scientificName: Psalmocharias rugipennis (Walker, 1858); **Location:** continent: Asia; country: Pakistan; locality: Quetta, Baluchistan; verbatimElevation: 5500 ft; **Event:** eventDate: 23/06/1930; **Record Level:** institutionCode: NHMUK; basisOfRecord: PreservedSpecimen

##### Distribution

[Metcalf, 1963] Hindustan; India; Pakistan; Baluchistan; Afghanistan; Northwestern India. [Sanborn, 2014] Pakistan, India, Afghanistan.

##### Notes

Authority: Walker 1858a; Species description states type specimen is male but the labelled type specimen in NHMUK is female. This observation agrees with Distant (1892a) which illustrates type specimen as a female.

#### Purana campanula

Pringle, 1955

Purana campanula Pringle, 1955

##### Materials

- **Type status:** Holotype. **Occurrence:** catalogNumber: BMNH(E) 1009415; recordedBy: J.W.S. Pringle; individualCount: 1; sex: male; **Taxon:** scientificName: Purana campanula Pringle, 1955; **Location:** continent: Asia; country: Sri Lanka; locality: Kanneliya (S. P.); **Event:** eventDate: 20/05/1953; **Record Level:** institutionCode: NHMUK; basisOfRecord: PreservedSpecimen

##### Distribution

[Duffles and van der Laan, 1985] Ceylon.

##### Notes

Authority: Pringle 1955

#### Purana guttularis

(Walker, 1858)

Cicada guttularis Walker, 1858

##### Materials

- **Type status:** Holotype. **Occurrence:** catalogNumber: BMNH(E) 1009414; individualCount: 1; sex: female; **Taxon:** scientificName: Purana guttularis (Walker, 1858); **Location:** continent: Asia; country: Myanmar; locality: Burma; **Record Level:** institutionCode: NHMUK; basisOfRecord: PreservedSpecimen

##### Distribution

[Metcalf, 1963] Burma; Eastern India; Philippine Islands; India; Nias; Borneo; Sarawak; Java; Assam; Tenasserim; Siam; Pahang; Malay Peninsula; Sumatra; Kwangtung. [Sanborn, 2014] Malaysia, Peninsular Malaysia, China, Burma, Philippines, Nias Island, Borneo, Sarawak, Java, Singapore, Sabah, Guangdong, Kalimantan, India, Vietnam, Siam, Thailand, Indonesia, Philippine Republic, Myanmar, Brunei, Mindanao, Sumatra.

##### Notes

Authority: Walker 1858b; Metcalf (1963) states "Eastern India, India, Assam" in reference to India orientalis. Lee (2010b) states that the type locality is Myanmar and that records from China, Philippines, Indonesia, Malaysia, and Thailand may have been based on mididentifications of closely allied species. Subsequent references to India in Sanborn (2014) are likely to be due to previous inaccurate literature localities.

#### Purana morrisi

(Distant, 1892)

Leptopsaltria morrisi Distant, 1892

##### Materials

- **Type status:** Holotype. **Occurrence:** catalogNumber: BMNH(E) 1009417; recordedBy: A.W. Morris; individualCount: 1; sex: male; **Taxon:** scientificName: Purana morrisi (Distant, 1892); **Location:** continent: Asia; country: India; locality: Shivarai Hills, Madras Province; **Record Level:** institutionCode: NHMUK; basisOfRecord: PreservedSpecimen

##### Distribution

[Metcalf, 1963] India; Madras. [Sanborn, 2014] India.

##### Notes

Authority: Distant 1892c

#### Purana tigrina

(Walker, 1850)

Dundubia tigrina Walker, 1850
Purana tigrina mjöbergi Moulton, 1923

##### Materials

- **Type status:** Holotype. **Occurrence:** catalogNumber: BMNH(E) 1009413; individualCount: 1; sex: male; **Taxon:** scientificName: Purana tigrina (Walker, 1850); **Location:** continent: Asia; country: India; locality: Malabar; **Record Level:** institutionCode: NHMUK; basisOfRecord: PreservedSpecimen

##### Distribution

[Distant, 1889/92] Continental India: Malabar; Trivandrum (Travankor). [Metcalf, 1963] Madras; Assam (?); India; Malay Peninsula; Travancore; Tibet; Malaya; Borneo; Malabar; Malacca; Singapore Island; Perak; Johore; Penang; Philippine Islands; Sumatra; Burma. [Sanborn, 2014] Pakistan, India, Tibet, Peninsular Malaysia, Malaysia, Borneo, Sarawak, Malabar, India, Tibet,, Philippines, Singapore, Sabah, Java, Madras, Sumatra, Burma, Thailand, Sundaland, Nias Island, Malayan Peninsula, Bunguran Island, Kalimantan Timur, Bunguran Island, Sundaland, Thailand, Tibet, Burma, Indonesia, Philippine Republic, Gogala Thailand, Malabar, Bangladesh.

##### Notes

Authority: Walker 1850

#### Pycna himalayana

(Naruse, 1977)

Suisha himalayana Naruse, 1977

##### Materials

- **Type status:** Holotype. **Occurrence:** individualCount: 1; sex: male; **Taxon:** scientificName: Pycna himalayana (Naruse, 1977); **Location:** continent: Asia; country: Nepal; locality: Ghora Tobela, Langtang Valley, Bagmati Zone; verbatimElevation: 3000 m; **Event:** eventDate: 23/09/1975; **Record Level:** institutionCode: EIHU; basisOfRecord: PreservedSpecimen

##### Distribution

[Duffels and van der Laan, 1985] Nepal. [Sanborn, 2014] Nepal.

##### Notes

Authority: Naruse in Naruse and Takagi 1977; An additional 1 male paratype (EIHU) was designated in the species description.

#### Pycna minor

Liu, 1940

Pycna minor Liu, 1940

##### Materials

- **Type status:** Holotype. **Occurrence:** recordedBy: Carleton; individualCount: 1; sex: male; **Taxon:** scientificName: Pycna minor Liu, 1940; **Location:** continent: Asia; country: India; locality: Koolloo; **Record Level:** institutionCode: MCZ; basisOfRecord: PreservedSpecimen
- **Type status:** Paratype. **Occurrence:** recordedBy: Carleton; individualCount: 1; sex: male; **Taxon:** scientificName: Pycna minor Liu, 1940; **Location:** continent: Asia; country: India; locality: Koolloo; **Record Level:** institutionCode: MCZ; basisOfRecord: PreservedSpecimen
- **Type status:** Paratype. **Occurrence:** recordedBy: Carleton; individualCount: 3; sex: female; **Taxon:** scientificName: Pycna minor Liu, 1940; **Location:** continent: Asia; country: India; locality: Koolloo; **Record Level:** institutionCode: MCZ; basisOfRecord: PreservedSpecimen

##### Distribution

[Liu, 1940] India: Koolloo. [Sanborn, 2014] India.

##### Notes

Authority: Liu 1940

#### Pycna montana

Hayashi, 1978

Pycna montana Hayashi, 1978

##### Materials

- **Type status:** Holotype. **Occurrence:** recordedBy: T. Fujioka; individualCount: 1; sex: male; **Taxon:** scientificName: Pycna montana Hayashi, 1978; **Location:** continent: Asia; country: Nepal; locality: Kambachen [to] Lhonak; verbatimElevation: 3900 - 4550 m; **Event:** eventDate: 16/07/1963; **Record Level:** institutionCode: NSMT; basisOfRecord: PreservedSpecimen

##### Distribution

[Duffels and van der Laan, 1985] Nepal. [Sanborn, 2014] Nepal.

##### Notes

Authority: Hayashi 1978; An additional 1 male paratype (private collection of Dr T. Fujikoa) was designated in the species description.

#### Pycna repanda repanda

(Linneaus, 1758)

Cicada repanda Linnaeus, 1758
Platypleura phalaenoides Walker, 1850
Platypleura interna Walker, 1852
Platyplerua congrex Butler, 1874

##### Materials

- **Type status:** Holotype. **Taxon:** scientificName: Pycna repanda repanda (Linnaeus, 1758); **Location:** continent: Asia; locality: Indiis; **Record Level:** basisOfRecord: PreservedSpecimen

##### Distribution

[Distant, 1889/92; Distant, 1906] Continental India: Kashmeer Valley; North Bengal; Sikkim; Darjeeling; Himalaya; Assam (Margherita and Naga Hills); Khasi Hills; Sylhet; Seebsagar. Burma: Kakhien Hills. [Metcalf, 1963] India; Uttar Pradesh; India (?); Bengal; Northern India; Assam; Java (?); Ceylon; Sylhet; Northern Bengal; Burma; Japan; Europe; Asia; Kashmir; Sikkim; Kyushu; Ryukyu Islands; Formosa; China; Malaya; Indonesia; Oriental Region; Southern Japan; Szechwan; Japan (?); Sikang; Punjab; Honshu. [Duffels and van der Laan, 1985] Himalaya; China; Nepal; Bhutan. [Sanborn, 2014] Cashmere, China, India, Pakistan, Nepal, Hunan, Shaanxi, Jiangxi, Zhejiang, Taiwan, Guizhou, Sichuan, Yunnan, Xizang, Kashmir, Sikkim, Burma, Bhutan, Tibet, Sri Lanka, Japan, Indonesia.

##### Notes

Authority: Linnaeus 1758

#### Pycna verna

Hayashi, 1982

Pycna verna Hayashi, 1982

##### Materials

- **Type status:** Holotype. **Occurrence:** recordedBy: T.C. Maa; individualCount: 1; sex: male; **Taxon:** scientificName: Pycna verna Hayashi, 1982; **Location:** continent: Asia; country: India; locality: Darjeeling, Bengal; verbatimElevation: 1400 m; **Event:** eventDate: 21/04/1938; **Record Level:** institutionCode: BPBM; basisOfRecord: PreservedSpecimen
- **Type status:** Paratype. **Occurrence:** recordedBy: T.C. Maa; individualCount: 2; sex: male; **Taxon:** scientificName: Pycna verna Hayashi, 1982; **Location:** continent: Asia; country: India; locality: Darjeeling, Bengal; verbatimElevation: 1400 m; **Event:** eventDate: 21/04/1938; **Record Level:** institutionCode: BPBM; basisOfRecord: PreservedSpecimen

##### Distribution

[Hayashi, 1982] India: Darjeeling. Nepal: Kathmandu Val, Gokarna Forest (1370 m). [Sanborn, 2014] India, Nepal.

##### Notes

Authority: Hayashi 1982; An additional 7 male paratypes (2 BPBM; 5 OMNH) were designated in the species description, however only the material from India is included in this paper.

#### Quintilia pomponia

Distant, 1912

Quintilia pomponia Distant, 1912

##### Materials

- **Type status:** Holotype. **Occurrence:** catalogNumber: BMNH(E) 1009610; individualCount: 1; sex: female; **Taxon:** scientificName: Quintilia pomponia Distant, 1912; **Location:** continent: Asia; country: India; locality: near Dehra Dun, N. India; **Event:** eventDate: ??/06/1909; **Record Level:** institutionCode: NHMUK; basisOfRecord: PreservedSpecimen

##### Distribution

[Metcalf, 1963] United Provinces; Northern India.

##### Notes

Authority: Distant 1912c

#### Rustia dentivitta

(Walker, 1862)

Cicada dentivitta Walker, 1862
Rustia pedunculata Stål, 1866
Tibicen amussitatus Distant, 1888

##### Materials

- **Type status:** Holotype. **Occurrence:** catalogNumber: BMNH(E) 1009557; recordedBy: Mouhot; sex: male; **Taxon:** scientificName: Rustia dentivitta (Walker, 1862); **Location:** continent: Asia; country: Thailand; locality: (Tha) Chaut; **Record Level:** institutionCode: NHMUK; basisOfRecord: PreservedSpecimen
- **Type status:** Other material. **Occurrence:** catalogNumber: BMNH(E) 1009558; recordedBy: William Doherty; individualCount: 1; sex: female; **Taxon:** scientificName: Rustia dentivitta (Walker, 1862); **Location:** continent: Asia; country: India; locality: Margherita, Upper Assam; **Record Level:** institutionCode: NHMUK; basisOfRecord: PreservedSpecimen

##### Distribution

[Distant, 1889/92] Continental India: Margherita (Assam). Burma: Rangoon. [Metcalf, 1963] Siam; Cambodia; India; Burma; Mysore; Assam; Himalayas; Indochina; Uttar Pradesh. [Duffels and van der Laan, 1985] Nepal. [Sanborn, 2014] Thailand, Cambodia, Indonesia, Borneo, Philippine Republic, Korea, Siam, Cambodia, Assam, Burma, India, Himalaya, Indochina, Nepal, Vietnam, Myanmar.

##### Notes

Authority: Walker 1862

#### Rustia tigrina

(Distant, 1888)

Tibicen tigrina Distant, 1888

##### Materials

- **Type status:** Holotype. **Occurrence:** catalogNumber: BMNH(E) 1009559; individualCount: 1; sex: male; **Taxon:** scientificName: Rustia tigrina (Distant, 1888); **Location:** continent: Asia; country: India; locality: Kulluur; **Event:** eventDate: ??/05/1888; **Record Level:** institutionCode: NHMUK; basisOfRecord: PreservedSpecimen

##### Distribution

[Distant, 1889/92] Continental India: Kulluur. [Metcalf, 1963] Burma; India.

##### Notes

Authority: Distant 1888d

#### Scieroptera crocea crocea

(Guérin-Méneville, 1838)

Cicada crocea Guérin-Méneville, 1838

##### Materials

- **Type status:** Holotype. **Taxon:** scientificName: Scieroptera crocea crocea (Guérin-Méneville, 1838); **Location:** continent: Asia; country: India; locality: Bengal; **Record Level:** basisOfRecord: PreservedSpecimen

##### Distribution

[Distant, 1889/92] India: Bombay. [Metcalf, 1963] Bengal; Asia; Malacca; India; Java; Borneo; Tonkin; Bombay; Sumatra; Malay Archipelago; Sarawak; Malaya; Sikkim; Assam; Burma; Tenasserim; Malay Peninsula. [Sanborn, 2014] China, Hainan, Guangxi, India, Indonesia, Kalimantan, North Vietnam, Malaysia, Java, Sumatra, Myanmar, Vietnam.

##### Notes

Authority: Guérin-Méneville 1838

#### Scieroptera fumigata

(Stål, 1854)

Huechys fumigata Stål, 1854

##### Materials

- **Type status:** Holotype. **Taxon:** scientificName: Scieroptera fumigata (Stål, 1854); **Location:** continent: Asia; locality: India Orientalis; **Record Level:** institutionCode: NHRS; basisOfRecord: PreservedSpecimen
- **Type status:** Other material. **Occurrence:** catalogNumber: BMNH(E) 1009588; recordedBy: William Doherty; individualCount: 1; sex: male; **Taxon:** scientificName: Scieroptera fumigata (Stål, 1854); **Location:** continent: Asia; country: India; locality: Margherita, Assam; **Record Level:** institutionCode: NHMUK; basisOfRecord: PreservedSpecimen

##### Distribution

[Distant, 1889/92] India: "Ind. Orient."; North Bengal; Margherita (Assam). [Metcalf, 1963] Eastern India; Hindustan; India; Bengal; Assam; East Indies; Northern Bengal.

##### Notes

Authority: Stål 1854

#### Scieroptera montana

Schmidt, 1918

Scieroptera montana Schmidt, 1918

##### Materials

- **Type status:** Holotype. **Occurrence:** recordedBy: Fruhstorfer; individualCount: 1; sex: male; **Taxon:** scientificName: Scieroptera montana Schmidt, 1918; **Location:** continent: Asia; country: India; locality: Darjeeling, Juni; **Event:** eventDate: ??/06/??; **Record Level:** institutionCode: MZPW; basisOfRecord: PreservedSpecimen

##### Distribution

[Metcalf, 1963] Bengal.

##### Notes

Authority: Schmidt 1918

#### Scieroptera splendidula cuprea

(Walker, 1870)

Huechys cuprea Walker, 1870
Scieroptera splendidula var. a Distant, 1982

##### Materials

- **Type status:** Holotype. **Occurrence:** sex: female; **Taxon:** scientificName: Scieroptera splendidula cuprea (Walker, 1870); **Location:** continent: Asia; country: Indonesia; locality: Tondano (on Celebes); **Record Level:** basisOfRecord: PreservedSpecimen

##### Distribution

[Distant, 1889/92] India: Sikkim; Khasi Hills. Burma: Momeit. [Metcalf, 1963] Celebes; Sikkim; Assam; Burma; Borneo; Siam; Sarawak; Hindustan; Java; Malaya; India; Bombay; Tenasserim; Philippine Islands; Formosa; Sunda Islands; China. [Sanborn, 2014] Thailand, India, Southeast Asia, Indonesia, China, Japan, Philippine Republic, Taiwan, Celebes, Siam.

##### Notes

Authority: Walker 1870

#### Scieroptera splendidula splendidula

(Fabricius, 1775)

Tettigonia splendidula Fabricius, 1775
Scieroptera splendidula vittata Kato, 1940

##### Materials

- **Type status:** Holotype. **Occurrence:** recordedBy: Dru Drury; **Taxon:** scientificName: Scieroptera splendidula splendidula (Fabricius, 1775); **Location:** continent: Asia; country: China; **Record Level:** basisOfRecord: PreservedSpecimen
- **Type status:** Other material. **Occurrence:** catalogNumber: BMNH(E) 1009589; recordedBy: W.F. Badgley; individualCount: 1; sex: male; **Taxon:** scientificName: Scieroptera splendidula splendidula (Fabricius, 1775); **Location:** continent: Asia; country: India; locality: Assam; **Event:** eventDate: ??/??/1906; **Record Level:** institutionCode: NHMUK; basisOfRecord: PreservedSpecimen

##### Distribution

[Distant, 1889/92] India: Margherita (Assam). Burma: Tenasserim. [Metcalf, 1963] China; Germany; Asia; East Indies; India; Java; Assam; Borneo; Hindustan; Tenasserim; Northern India; Silhet; Nias; Celebes; Laos; Indochina; Sikkim; Malay Region; Oriental Region; Formosa; Sunda Islands; Kwangsi; Northern Bengal; Sumatra; Hainan; Kwangtung; Fukien; Bihar; Uttar Pradesh; Burma; Philippine Islands. [Duffels and van der Laan, 1985] Nepal; China. [Sanborn, 2014] China, Kwangsi, Tien-mushan, Sikhim, Assam, Margherita, Khasi, Hills Burma, Momeit, Tenasserim, Java, Borneo, Celebes, Formosa, Hunan, Peninsular Malaysia, Malaysia, Sumatra, India, Java, Borneo, Sarawak, Langkawi Island, Zhejiang, Fujian, Taiwan, Guangdong, Hunan, Guizhou, Guangxi, Sichuan, Indonesia, Kalimantan, Celebes, Burma, Sabah, Manipur, Meghalaya, Vietnam, Thailand, Tenasserim, Kwangsi, Burma, Hainan, Kwangtung, Nepal, Philippines, Laos, Myanmar.

##### Notes

Authority: Fabricius 1775

#### Scieroptera splendidulavar.c

Distant, 1892

Scieroptera splendidula var. c Distant, 1892

##### Materials

- **Type status:** Holotype. **Taxon:** scientificName: Scieroptera splendidula var. c Distant, 1892; **Location:** continent: Asia; country: India; locality: North India; **Record Level:** basisOfRecord: PreservedSpecimen

##### Distribution

[Metcalf, 1963] Northern India; India.

##### Notes

Authority: Distant 1892c

#### Scieroptera splendidulavar.d

Fabricius, 1775

Scieroptera splendidula var. d Distant, 1892

##### Materials

- **Type status:** Holotype. **Taxon:** scientificName: Scieroptera splendidula var. d Distant, 1892; **Location:** continent: Asia; country: Myanmar; locality: Momeit [Mong Mit]; **Record Level:** basisOfRecord: PreservedSpecimen

##### Distribution

[Distant, 1889/92] Burma: Momeit. [Metcalf, 1963] Burma; India.

##### Notes

Authority: Fabricius 1775; Metcalf (1963) states India in references to Kato (1932) who listed the distribution as "lndia" when referring to Burma as it was considered part of "British India" at the time. Currently there are no known records from modern day India.

#### Subtibicina tigris

Lee, 2012

Subtibicina tigris Lee, 2012

##### Materials

- **Type status:** Holotype. **Occurrence:** recordedBy: R. Oberthur; individualCount: 1; sex: male; **Taxon:** scientificName: Subtibicina tigris Lee, 2012; **Location:** continent: Asia; country: India; locality: Phedong; **Event:** eventDate: 18/04/1883; **Record Level:** institutionCode: IRSNB; basisOfRecord: PreservedSpecimen

##### Distribution

[Lee, 2012] India.

##### Notes

Authority: Lee 2012a

#### Sulphogaeana sulphurea

(Westwood, 1839)

Cicada sulphurea Westwood, 1839
Cicada pulchella Westwood, 1942

##### Materials

- **Type status:** Holotype. **Taxon:** scientificName: Sulphogaeana sulphurea (Westwood, 1839); **Location:** continent: Asia; country: India; **Record Level:** institutionCode: OUMNH; basisOfRecord: PreservedSpecimen
- **Type status:** Other material. **Occurrence:** catalogNumber: BMNH(E) 1009592; recordedBy: F. Kingdon Ward; individualCount: 1; sex: female; **Taxon:** scientificName: Sulphogaeana sulphurea (Westwood, 1839); **Location:** continent: Asia; country: Myanmar; locality: Nam Tamai, Burma; verbatimElevation: 4500 ft; **Event:** eventDate: 7/05/1926; **Record Level:** institutionCode: NHMUK; basisOfRecord: PreservedSpecimen

##### Distribution

[Distant, 1889/92] Continental India: Sikkim; Nepal; Darjeeling. [Metcalf, 1963] India; Himalaya; Asia; Nepal; Northern India; Sikkim; Mysore; Bengal; Assam; Uttar Pradesh. [Duffels and van der Laan, 1985] Nepal; Bhutan. [Sanborn, 2014] India, China, Yunnan, Sikkim, Bangladesh, Nepal.

##### Notes

Authority: Westwood 1839

#### Talainga binghami

Distant, 1890

Talainga binghami Distant, 1890

##### Materials

- **Type status:** Syntype. **Occurrence:** catalogNumber: BMNH(E) 1009618; recordedBy: C.T. Bingham; sex: female; **Taxon:** scientificName: Talainga binghami Distant, 1890; **Location:** continent: Asia; country: Myanmar; locality: Karen Hills, Burma; **Event:** eventDate: ??/03/1887; **Record Level:** institutionCode: NHMUK; basisOfRecord: PreservedSpecimen
- **Type status:** Other material. **Occurrence:** catalogNumber: BMNH(E) 1009600; occurrenceRemarks: Specimen not part of type series, see taxon notes.; recordedBy: R.V. de Salvaza; individualCount: 1; sex: male; **Taxon:** scientificName: Talainga binghami Distant, 1890; **Location:** continent: Asia; country: Vietnam; locality: Chapa., Tonkin; **Event:** eventDate: ??/05-06/1916; **Record Level:** institutionCode: NHMUK; basisOfRecord: PreservedSpecimen

##### Distribution

[Metcalf, 1963] Burma; Indochina; Tonkin; China. [Sanborn, 2014] China, Vietnam, Jiangxi, Yunnan, Burma, Thailand, Indochina, Tonkin, Myanmar.

##### Notes

Authority: Distant 1890b; In the NHMUK there is a male specimen bearing a type label, however the type series is comprised of multiple females from "Burma, Kr. Hills (Bingham)".

#### Talainga japrona

Ollenbach, 1929

Talainga japrona Ollenbach, 1929

##### Materials

- **Type status:** Holotype. **Occurrence:** individualCount: 1; sex: female; **Taxon:** scientificName: Talainga japrona Ollenbach, 1929; **Location:** continent: Asia; country: India; locality: Jakhama, Japro, Naga Hills; verbatimElevation: 5500 ft; **Record Level:** institutionCode: IFRI; basisOfRecord: PreservedSpecimen

##### Distribution

[Metcalf, 1963] Assam; Burma; India.

##### Notes

Authority: Ollenbach 1929

#### Talainga naga

Ollenbach, 1929

Talainga naga Ollenbach, 1929

##### Materials

- **Type status:** Syntype. **Occurrence:** sex: male; **Taxon:** scientificName: Talainga naga Ollenbach, 1929; **Location:** continent: Asia; country: India; locality: Kohima, Naga Hills (on the Manipur Road); verbatimElevation: 5000 ft; **Event:** eventDate: ??/04/1924; **Record Level:** institutionCode: IFRI; basisOfRecord: PreservedSpecimen
- **Type status:** Syntype. **Occurrence:** sex: female; **Taxon:** scientificName: Talainga naga Ollenbach, 1929; **Location:** continent: Asia; country: India; locality: Kohima, Naga Hills (on the Manipur Road); verbatimElevation: 5000 ft; **Event:** eventDate: ??/04/1924; **Record Level:** institutionCode: IFRI; basisOfRecord: PreservedSpecimen

##### Distribution

[Metcalf, 1963] Assam; Burma; India.

##### Notes

Authority: Ollenbach 1929

#### Tanna bhutanensis

Distant, 1912

Tanna bhutanensis Distant, 1912

##### Materials

- **Type status:** Holotype. **Occurrence:** catalogNumber: BMNH(E) 1009434; recordedBy: R. Oberthur; individualCount: 1; sex: male; **Taxon:** scientificName: Tanna bhutanensis Distant, 1912; **Location:** continent: Asia; country: Bhutan; locality: Bhoutan Anglais; **Event:** eventDate: ??/??/1900; **Record Level:** institutionCode: NHMUK; basisOfRecord: PreservedSpecimen

##### Distribution

[Metcalf, 1963] Bhutan; China. [Duffels and van der Laan, 1985] Nepal.

##### Notes

Authority: Distant 1912d

#### Tanna minor

Hayashi, 1978

Tanna minor Hayashi, 1978

##### Materials

- **Type status:** Holotype. **Occurrence:** recordedBy: T. Aoki and S. Yamaguchi; individualCount: 1; sex: male; **Taxon:** scientificName: Tanna minor Hayashi, 1978; **Location:** continent: Asia; country: Nepal; locality: Dana, C. Nepal; verbatimElevation: 1600 m; **Event:** eventDate: 5/06/1974; **Record Level:** institutionCode: NSMT; basisOfRecord: PreservedSpecimen
- **Type status:** Paratype. **Occurrence:** recordedBy: T. Aoki and S. Yamaguchi; individualCount: 1; sex: female; **Taxon:** scientificName: Tanna minor Hayashi, 1978; **Location:** continent: Asia; country: Nepal; locality: Hinku, C. Nepal; verbatimElevation: 2640 m; **Event:** eventDate: 16/06/1974; **Record Level:** institutionCode: NSMT; basisOfRecord: PreservedSpecimen

##### Distribution

[Duffels and van der Laan, 1985] Nepal.

##### Notes

Authority: Hayashi 1978

#### Taungia abnormis

Ollenbach, 1929

Taungia abnormis Ollenbach, 1929

##### Materials

- **Type status:** Holotype. **Occurrence:** recordedBy: W.S. Wood; individualCount: 1; sex: female; **Taxon:** scientificName: Taungia abnormis Ollenbach, 1929; **Location:** continent: Asia; country: Myanmar; locality: Karen Hills, Thandaung; **Record Level:** institutionCode: IFRI; basisOfRecord: PreservedSpecimen

##### Distribution

[Metcalf, 1963] Burma; India.

##### Notes

Authority: Ollenbach 1929; Metcalf (1963) listed India in reference Burma being part of "British India". The type locality (Thandaung) is within modern day Myanmar and the species has not yet been recorded from modern day India.

#### Terpnosia abdullah

Distant, 1904

Terpnosia abdullah Distant, 1904

##### Materials

- **Type status:** Holotype. **Occurrence:** recordedBy: Craddock; sex: male; **Taxon:** scientificName: Terpnosia abdullah Distant, 1904; **Location:** continent: Asia; country: Malaysia; locality: Pahang, Malay Peninsula; **Record Level:** basisOfRecord: PreservedSpecimen

##### Distribution

[Distant, 1906] India: N.W. India and Sikkim. [Metcalf, 1963] Malay Peninsula; India; Sikkim; Northern India; North-western India; Java (?); Pahang; Java; Malaya; Uttar Pradesh. [Sanborn, 2014] Thailand, Malay, Peninsula, India, Sikkim, Malaysia, Java.

##### Notes

Authority: Distant 1904b

#### Terpnosia clio

(Walker, 1850)

Dundubia clio Walker, 1850

##### Materials

- **Type status:** Syntype. **Occurrence:** catalogNumber: BMNH(E) 1009547; sex: male; **Taxon:** scientificName: Terpnosia clio (Walker, 1850); **Location:** locality: Locality unknown; **Record Level:** institutionCode: NHMUK; basisOfRecord: PreservedSpecimen

##### Distribution

[Distant, 1889/92] India: Sikkim. [Metcalf, 1963] India; Sikkim; Mysore; Burma; Tenasserim; Indochina; Yunnan; Uttar Pradesh; Western Himalayas; China. [Duffels and van der Laan, 1985] Nepal; China. [Sanborn, 2014] China, Yunnan, Himalaya, Sikkim, Burma, Tenasserim, Sikkim, India, Nepal, Southeast Asia, Myanmar.

##### Notes

Authority: Walker 1850

#### Terpnosia collina

(Distant, 1888)

Pomponia collina Distant, 1888

##### Materials

- **Type status:** Syntype. **Occurrence:** catalogNumber: BMNH(E) 1009546; sex: male; **Taxon:** scientificName: Terpnosia collina (Distant, 1888); **Location:** continent: Asia; country: India; locality: North Khasi Hills; **Record Level:** institutionCode: NHMUK; basisOfRecord: PreservedSpecimen

##### Distribution

[Distant, 1889/92] Continental India: Khasi Hills; Burma: Ruby Mines. [Metcalf, 1963] Assam; India; Burma. [Duffels and van der Laan, 1985] Nepal.

##### Notes

Authority: Distant 1888c

#### Terpnosia confusa

Distant, 1905

Terpnosia confusa Distant, 1905

##### Materials

- **Type status:** Syntype. **Occurrence:** catalogNumber: BMNH(E) 1009540; sex: male; **Taxon:** scientificName: Terpnosia confusa Distant, 1905; **Location:** continent: Asia; country: India; **Record Level:** institutionCode: NHMUK; basisOfRecord: PreservedSpecimen
- **Type status:** Syntype. **Occurrence:** sex: male; **Taxon:** scientificName: Terpnosia confusa Distant, 1905; **Location:** continent: Asia; country: India; locality: Sikkim; **Record Level:** basisOfRecord: PreservedSpecimen

##### Distribution

[Distant, 1905] India: Sikkim. [Metcalf, 1963] India; Ceylon; Java; Sikkim.

##### Notes

Authority: Distant 1905b

#### Terpnosia elegans

(Kirby, 1891)

Pomponia elegans Kirby, 1891

##### Materials

- **Type status:** Holotype. **Occurrence:** catalogNumber: BMNH(E) 1009542; individualCount: 1; sex: male; **Taxon:** scientificName: Terpnosia elegans (Kirby, 1891); **Location:** continent: Asia; country: Sri Lanka; locality: Kandapola near Kandy; **Record Level:** institutionCode: NHMUK; basisOfRecord: PreservedSpecimen

##### Distribution

[Metcalf, 1963] Ceylon.

##### Notes

Authority: Kirby 1891

#### Terpnosia ganesa

Distant, 1904

Terpnosia ganesa Distant, 1904

##### Materials

- **Type status:** Holotype. **Occurrence:** catalogNumber: BMNH(E) 1009537; recordedBy: P.W. Mackinnon; individualCount: 1; sex: male; **Taxon:** scientificName: Terpnosia ganesa Distant, 1904; **Location:** continent: Asia; country: India; locality: Masuri [Mussoorie]; **Event:** eventDate: 15/04/1903; **Record Level:** institutionCode: NHMUK; basisOfRecord: PreservedSpecimen

##### Distribution

[Metcalf, 1963] Mysore; India; Uttar Pradesh. [Duffels and van der Laan, 1985] Nepal.

##### Notes

Authority: Distant 1904b

#### Terpnosia jenkinsi

Distant, 1912

Terpnosia jenkinsi Distant, 1912

##### Materials

- **Type status:** Syntype. **Occurrence:** catalogNumber: BMNH(E) 1009552; recordedBy: J.T. Jenkins; sex: male; **Taxon:** scientificName: Terpnosia jenkinsi Distant, 1912; **Location:** continent: Asia; country: India; locality: Paresnath, W. Bengal; verbatimElevation: 4000 - 4400 ft; **Event:** eventDate: ??/05/1909; **Record Level:** institutionCode: NHMUK; basisOfRecord: PreservedSpecimen
- **Type status:** Syntype. **Occurrence:** recordedBy: J.T. Jenkins; sex: male; **Taxon:** scientificName: Terpnosia jenkinsi Distant, 1912; **Location:** continent: Asia; country: India; locality: Paresnath, W. Bengal; verbatimElevation: 4000 - 4400 ft; **Record Level:** institutionCode: NZSI; basisOfRecord: PreservedSpecimen

##### Distribution

[Metcalf, 1963] Bengal.

##### Notes

Authority: Distant 1912d; Multiple specimens (number unknown) used in species description.

#### Terpnosia lactea

(Distant, 1887)

Leptopsaltria lactea Distant, 1887

##### Materials

- **Type status:** Holotype. **Occurrence:** catalogNumber: BMNH(E) 1009402; individualCount: 1; sex: male; **Taxon:** scientificName: Terpnosia lactea (Distant, 1887); **Location:** continent: Asia; country: Indonesia; locality: Sumatra; **Event:** eventDate: ??/02/??; **Record Level:** institutionCode: NHMUK; basisOfRecord: PreservedSpecimen

##### Distribution

[Metcalf, 1963] Sumatra; Malay Peninsula; Sikkim; Java; Borneo; Malay States; India; Malaya; Perak; Malacca; Singapore Island; Johore; Penang; Indochina; Brunei; Mentawai Islands; Sipora; Borneo (?). [Sanborn, 2014] Malaysia, Borneo, Sabah, Peninsular Malaysia, Sumatra, Brunei, Java, Sarawak, Thailand, Indonesia, Indochina, Philippines, Palawan, Vietnam, Malay Peninsula, Northern India.

##### Notes

Authority: Distant 1887b

#### Terpnosia maculipes

(Walker, 1850)

Dundubia maculipes Walker, 1850

##### Materials

- **Type status:** Holotype. **Occurrence:** catalogNumber: BMNH(E) 1009536; individualCount: 1; sex: male; **Taxon:** scientificName: Terpnosia maculipes (Walker, 1850); **Location:** continent: Asia; country: India; locality: N. Bengal; **Record Level:** institutionCode: NHMUK; basisOfRecord: PreservedSpecimen

##### Distribution

[Metcalf, 1963] Bengal; India; Northern Bengal; Burma; Mysore; Uttar Pradesh. [Sanborn, 2014] India.

##### Notes

Authority: Walker 1850

#### Terpnosia polei

(Henry, 1931)

Pomponia polei Henry, 1931

##### Materials

- **Type status:** Holotype. **Occurrence:** catalogNumber: BMNH(E) 1009399; individualCount: 1; sex: male; **Taxon:** scientificName: Terpnosia polei (Henry, 1931); **Location:** continent: Asia; country: Sri Lanka; locality: Morningside Estate, Rakwana; **Event:** eventDate: 8/05/1929; **Record Level:** institutionCode: NHMUK; basisOfRecord: PreservedSpecimen
- **Type status:** Paratype. **Occurrence:** catalogNumber: BMNH(E) 1009400; recordedBy: J. Pole; individualCount: 1; sex: female; **Taxon:** scientificName: Terpnosia polei (Henry, 1931); **Location:** continent: Asia; country: Sri Lanka; locality: Maskeliya; **Event:** eventDate: ??/05/??; **Record Level:** institutionCode: NHMUK; basisOfRecord: PreservedSpecimen
- **Type status:** Paratype. **Occurrence:** individualCount: 2; sex: male; **Taxon:** scientificName: Terpnosia polei (Henry, 1931); **Location:** continent: Asia; country: Sri Lanka; locality: Morningside Estate, Rakwana; **Record Level:** institutionCode: CNMS; basisOfRecord: PreservedSpecimen
- **Type status:** Paratype. **Occurrence:** recordedBy: J. Pole; individualCount: 1; sex: female; **Taxon:** scientificName: Terpnosia polei (Henry, 1931); **Location:** continent: Asia; country: Sri Lanka; locality: Maskeliya; **Record Level:** institutionCode: CNMS; basisOfRecord: PreservedSpecimen

##### Distribution

[Metcalf, 1963] Ceylon.

##### Notes

Authority: Henry 1931

#### Terpnosia ransonneti

(Distant, 1888)

Pomponia ransonneti Distant, 1888
Pomponia greeni Kirby, 1891

##### Materials

- **Type status:** Holotype. **Occurrence:** catalogNumber: BMNH(E) 1009554; individualCount: 1; sex: male; **Taxon:** scientificName: Terpnosia ransonneti (Distant, 1888); **Location:** continent: Asia; country: Sri Lanka; locality: Colombo; **Record Level:** institutionCode: NHMUK; basisOfRecord: PreservedSpecimen

##### Distribution

[Distant, 1906] India: Mussooree. [Metcalf, 1963] Ceylon; Mysore; Indochina; India; Uttar Pradesh. [Duffels and van der Laan, 1985] Ceylon. [Sanborn, 2014] Japan, Vietnam, Sri Lanka, India.

##### Notes

Authority: Distant 1888c

#### Terpnosia ridens

Pringle, 1955

Terpnosia ridens Pringle, 1955

##### Materials

- **Type status:** Holotype. **Occurrence:** catalogNumber: BMNH(E) 1009541; recordedBy: J.W.S. Pringle; individualCount: 1; sex: male; **Taxon:** scientificName: Terpnosia ridens Pringle, 1955; **Location:** continent: Asia; country: Sri Lanka; locality: Inginiyagala, E. Province; **Event:** eventDate: 10/06/1953; **Record Level:** institutionCode: NHMUK; basisOfRecord: PreservedSpecimen

##### Distribution

[Duffles and van der Laan, 1985] Ceylon.

##### Notes

Authority: Pringle 1955

#### Terpnosia stipata

(Walker, 1850)

Dundubia stipata Walker, 1850
Dundubia clonia Walker, 1850

##### Materials

- **Type status:** Holotype. **Occurrence:** catalogNumber: BMNH(E) 1009544; sex: male; **Taxon:** scientificName: Terpnosia stipata (Walker, 1850); **Location:** continent: Asia; country: Sri Lanka; **Record Level:** institutionCode: NHMUK; basisOfRecord: PreservedSpecimen

##### Distribution

[Distant, 1889/92] Ceylon: Ritagala (2500 ft). [Metcalf, 1963] Ceylon. [Duffels and van der Laan, 1985] Ceylon. [Sanborn, 2014] Sri Lanka.

##### Notes

Authority: Walker 1850

#### Tibeta zenobia

(Distant, 1912)

Melampsalta zenobia Distant, 1912
Melampsalta kulingana Kato, 1938

##### Materials

- **Type status:** Syntype. **Occurrence:** catalogNumber: BMNH(E) 1009615; sex: male; **Taxon:** scientificName: Tibeta zenobia (Distant, 1912); **Location:** continent: Asia; country: Nepal; locality: Gowchar; **Record Level:** institutionCode: NHMUK; basisOfRecord: PreservedSpecimen
- **Type status:** Syntype. **Taxon:** scientificName: Tibeta zenobia (Distant, 1912); **Location:** continent: Asia; country: Nepal; locality: Thankote; **Record Level:** basisOfRecord: PreservedSpecimen
- **Type status:** Syntype. **Taxon:** scientificName: Tibeta zenobia (Distant, 1912); **Location:** continent: Asia; country: Nepal; locality: Nagorkote; **Record Level:** basisOfRecord: PreservedSpecimen

##### Distribution

[Distant, 1916] Nepal: Gowchar; Thankote; Nagorkote. [Metcalf, 1963] Nepal. [Duffels and van der Laan, 1985] Nepal. [Sanborn, 2014] China, Jiangxi, Zhejiang, Yunnan, Xizang, Vietnam.

##### Notes

Authority: Distant 1912c

#### Tibicina casyapae

(Distant, 1888)

Tibicen casyapae Distant, 1888

##### Materials

- **Type status:** Holotype. **Occurrence:** catalogNumber: BMNH(E) 1009603; recordedBy: Leech; individualCount: 1; sex: male; **Taxon:** scientificName: Tibicina casyapae (Distant, 1888); **Location:** continent: Asia; country: India; locality: Cashmere Valley; verbatimElevation: 6300 ft; **Record Level:** institutionCode: NHMUK; basisOfRecord: PreservedSpecimen

##### Distribution

[Metcalf, 1963] Kashmir; India; Northern India; Afghanistan. [Duffels and van der Laan, 1985] Afghanistan; Kashmir. [Sanborn, 2014] Afghanistan, India, Pakistan.

##### Notes

Authority: Distant 1888c; Species description states female but the type specimen in NHMUK is male.

#### Tibicina reticulata

(Distant, 1888)

Tibicen reticulatus Distant, 1888

##### Materials

- **Type status:** Holotype. **Occurrence:** catalogNumber: BMNH(E) 1009601; individualCount: 1; sex: male; **Taxon:** scientificName: Tibicina reticulata (Distant, 1888); **Location:** continent: Asia; country: Pakistan; locality: Gilgit; **Record Level:** institutionCode: NHMUK; basisOfRecord: PreservedSpecimen

##### Distribution

[Metcalf, 1963] Kashmir; India; Punjab; Oriental Regions; Uttar Pradesh. [Sanborn, 2014] Pakistan, India.

##### Notes

Authority: Distant 1888c; Species description describes the type specimen as a female, however it is male (Distant 1892a; Pl. XIV, Figs. 21, 21a-b).

#### Tosena albata

Distant, 1878

Tosena var. albata Distant, 1878
Tosena melanoptera var. c Distant, 1906

##### Materials

- **Type status:** Holotype. **Occurrence:** catalogNumber: BMNH(E) 1009377; individualCount: 1; sex: male; **Taxon:** scientificName: Tosena albata Distant, 1878; **Location:** continent: Asia; country: India; locality: N.W. Himalaya; **Record Level:** institutionCode: NHMUK; basisOfRecord: PreservedSpecimen

##### Distribution

[Metcalf, 1963] Himalaya; Northern India; Northwestern Himalayas; India. [Sanborn, 2014] Thailand, Nepal, India, Himalayas, Vietnam, Burma.

##### Notes

Authority: Distant 1878a

#### Tosena dives

(Westwood, 1842)

Cicada dives Westwood, 1842
Huechys transversa Walker, 1858

##### Materials

- **Type status:** Holotype. **Taxon:** scientificName: Tosena dives (Westwood, 1842); **Location:** continent: Asia; country: Bangladesh; locality: Sylhet, East Indies; **Record Level:** institutionCode: OUMNH; basisOfRecord: PreservedSpecimen
- **Type status:** Other material. **Occurrence:** individualCount: 1; sex: male; **Taxon:** scientificName: Tosena dives (Westwood, 1842); **Location:** locality: Locality unknown; **Record Level:** institutionCode: NHMUK; basisOfRecord: PreservedSpecimen

##### Distribution

[Distant, 1889/92] Continental India: Sikkim; Darjeeling; Sylhet. [Metcalf, 1963] Sylhet; East Indies; Himalayas; India; Hindustan; Sikkim; Bengal; Northern Bengal; Assam. [Duffels and van der Laan, 1985] Nepal. [Sanborn, 2014] Bhutan.

##### Notes

Authority: Westwood 1842

#### Tosena mearesiana

(Westwood, 1842)

Cicada mearesiana Westwood, 1842

##### Materials

- **Type status:** Holotype. **Occurrence:** recordedBy: D. Meares; **Taxon:** scientificName: Tosena mearesiana (Westwood, 1842); **Location:** continent: Asia; country: India; locality: Himalayas; **Record Level:** institutionCode: NHMUK; basisOfRecord: PreservedSpecimen

##### Distribution

[Distant, 1889/92] Continental India: Himalaya; Sikkim. [Metcalf, 1963] Himalayas; Sylhet; India; Northern India; Sikkim; Bengal; Malay Archipelago (?). [Duffels and van der Laan, 1985] Nepal.

##### Notes

Authority: Westwood 1842

#### Tosena melanoptera

(White, 1846)

Cicada (Tosena) melanoptera White, 1846
Tosena fasciata Moulton, 1923 (nec Fabricius, 1787)
Tosena melanopteryx Kirkaldy, 1909
Tosena fasciata var. d Distant, 1889

##### Materials

- **Type status:** Syntype. **Occurrence:** individualCount: 1; **Taxon:** scientificName: Tosena melanoptera (White, 1846); **Location:** continent: Asia; country: India; locality: N. India; **Record Level:** institutionCode: NHMUK; basisOfRecord: PreservedSpecimen
- **Type status:** Syntype. **Taxon:** scientificName: Tosena melanoptera (White, 1846); **Location:** continent: Asia; country: Bangladesh; locality: Silhet; **Record Level:** institutionCode: NHMUK; basisOfRecord: PreservedSpecimen

##### Distribution

[Distant, 1889/92] India: N.W. Himalaya; Sikkim; Darjeeling; N. Khasi Hills; Seebsagar; Sylhet (Bangladesh). Burma: Bhamo. [Metcalf, 1963] Assam; Northern India; Sylhet; Sibsagar; Sikkim; India; Burma; Tonkin; Bengal; Indochina; Himalayas; Upper Burma; Northwestern Himalayas. [Sanborn, 2014] China, Guangxi, Xizang, India, Sikkim, Burma, Bangladesh, Himalaya, Thailand, Vietnam, Indochina, Nepal, Laos, Myanmar.

##### Notes

Authority: White 1846

#### Zaphsa princeps

Lee & Emery, 2014

Zaphsa princeps Lee & Emery, 2014

##### Materials

- **Type status:** Holotype. **Occurrence:** catalogNumber: MNHN (EH) 16437; individualCount: 1; sex: male; **Taxon:** scientificName: Zaphsa princeps Lee & Emery, 2014; **Location:** continent: Asia; country: India; locality: Javadu Hills, Kavalore; verbatimElevation: 600 m; **Event:** eventDate: ??/06/2006; **Record Level:** institutionCode: MNHN; basisOfRecord: PreservedSpecimen
- **Type status:** Paratype. **Occurrence:** individualCount: 2; sex: female; **Taxon:** scientificName: Zaphsa princeps Lee & Emery, 2014; **Location:** continent: Asia; country: India; locality: Javadu Hills, Kavalore; verbatimElevation: 600 m; **Event:** eventDate: ??/06/2006; **Record Level:** institutionCode: AMS; basisOfRecord: PreservedSpecimen
- **Type status:** Paratype. **Occurrence:** individualCount: 3; sex: male; **Taxon:** scientificName: Zaphsa princeps Lee & Emery, 2014; **Location:** continent: Asia; country: India; locality: Javadu Hills, Vellore Kavalore; verbatimElevation: 600 m; **Event:** eventDate: ??/06/2006; **Record Level:** institutionCode: AMS; basisOfRecord: PreservedSpecimen

##### Distribution

[Lee and Emery, 2014] India.

##### Notes

Authority: Lee and Emery 2014b

### Checklist 2 - Species removed from the fauna of India, Bangladesh, Bhutan, Myanmar, Nepal and Sri Lanka

#### Abricta brunnea

(Fabricius, 1798)

Tettigonia brunnea Fabricius, 1798

##### Materials

- **Type status:** Holotype. **Occurrence:** recordedBy: Dom. Daldorff; **Taxon:** scientificName: Abricta brunnea (Fabricius, 1798); **Location:** continent: Africa; country: Mauritius; locality: Isle de France; **Record Level:** basisOfRecord: PreservedSpecimen

##### Distribution

[Metcalf, 1963] Mauritius; Italian Somaliland; Bengal. [Sanborn, 2014] Mauritius, Reuinion Island, Réunion Island.

##### Notes

Authority: Fabricius 1798; Not from India: the record of "Bengal" stems from the incorrect synonymy with Cicada maculicollis Guérin-Méneville 1838 by Stål (1866b).

#### Abricta pusilla

(Fabricius, 1803)

Tettigonia pusilla Fabricius, 1803

##### Materials

- **Type status:** Holotype. **Occurrence:** recordedBy: Dom. Billardiere; individualCount: 1; sex: female; **Taxon:** scientificName: Abricta pusilla (Fabricius, 1803); **Location:** continent: Asia; country: Indonesia; locality: Amboina; **Record Level:** institutionCode: ZMUC; basisOfRecord: PreservedSpecimen

##### Distribution

[Metcalf, 1963] Amboina; India; Molucca Islands.

##### Notes

Authority: Fabricius 1803; Not from India: Incorrectly listed from "Amboina, India" by Atkinson (1886), which is in Indonesia.

#### Birrima varians

(Germar, 1834)

Cicada varians Germar, 1834

##### Materials

- **Type status:** Holotype. **Occurrence:** recordedBy: Hope; **Taxon:** scientificName: Birrima varians (Germar, 1834); **Location:** continent: Oceania; locality: Australasia; **Record Level:** basisOfRecord: PreservedSpecimen

##### Distribution

[Metcalf, 1963] Australia; Assam; Silhet; Queensland. [Sanborn, 2014] Queensland, Australia, New South Wales, Australia.

##### Notes

Authority: Germar 1834; Not from India: Listed from Silhet (Assam) by Walker (1852) and then Atkinson (1884) in error when examining misidentified specimens of Dundubia vaginata (Metcalf 1963). Birrima varians is found in Australia.

#### Chremistica bimaculata

(Olivier, 1790)

Cicada bimaculata Olivier, 1790
Cicada atrovirens Guérin-Méneville, 1838

##### Materials

- **Type status:** Holotype. **Taxon:** scientificName: Chremistica bimaculata (Olivier, 1790); **Location:** continent: Asia; country: Indonesia; locality: Java; **Record Level:** basisOfRecord: PreservedSpecimen

##### Distribution

[Metcalf, 1963] Java; China; Ceylon; Philippine Islands; Cambodia; Tonkin; Malay States; Malaya; Indochina; Borneo. [Sanborn, 2014] Borneo, Sabah, Java, Cambodia, Tonkin, Philippines, Sarawak, Peninsular Malaysia, Cambodia, Boulard, Malaysia, Sundaland, Thailand.

##### Notes

Authority: Olivier 1790; Not from Sri Lanka: An error resulting from Cicada viridis (Fabricius, 1803) being synonymised in error with, and thereby linking the distributions of Cicada atrovirens Guérin-Méneville, 1838 and Chremistica mixta (Kirby, 1891) by Distant (1892a). Of these only Chremistica mixta (Kirby, 1891) is from Sri Lanka.

#### Chremistica ochracea ochracea

(Walker, 1850)

Fidicina ochracea Walker, 1850
Cicada ferrifera Walker, 1850
Dundubia fasciceps Stål, 1854

##### Materials

- **Type status:** Holotype. **Occurrence:** sex: male; **Taxon:** scientificName: Chremistica ochracea ochracea (Walker, 1850); **Location:** continent: Asia; locality: Locality unknown; **Record Level:** basisOfRecord: PreservedSpecimen

##### Distribution

[Metcalf, 1963] China; Formosa; Japan; Macao; Kwangtung; India; Malaya. [Sanborn, 2014] China, Fujian, Taiwan, Guangdong, Guangxi, India, Macao, Hong Kong, Japan, Malaysia, Thailand.

##### Notes

Authority: Walker 1850; Unlikely to be from India: Metcalf (1963) stated India in reference to Kato (1927) who listed a distribution without referencing specimens and this is likely an error. Subsequent "India" records in Sanborn (2014), by Boulard (2001a) and Sanborn et al. (2007) are likely a result of the Metcalf (1963) inclusion.

#### Chremistica tridentigera

(Breddin, 1905)

Cicada tridentigera Breddin, 1905

##### Materials

- **Type status:** Lectotype. **Occurrence:** individualCount: 1; sex: male; **Taxon:** scientificName: Chremistica tridentigera (Breddin, 1905); **Location:** continent: Asia; country: Malaysia; locality: Banguey; **Record Level:** institutionCode: MZH; basisOfRecord: PreservedSpecimen

##### Distribution

[Metcalf, 1963] Banguey [Sanborn, 2014] Kalimantan, Brunei, Southeast Asia, Malaysian Archipelago, Philippines, India, Sri Lanka, Borneo, Sabah, Sarawak, Banguey Island, Sumatra, Sundaland.

##### Notes

Authority: Breddin 1905; Additional paralectotypes designated in the species description: 1 male, 3 female (ZMH); 7 male (NHMUK). Not from India: Sanborn (2014) states India in reference to Bregman (1985), however India is listed for the Chremistica tridentigera group as a whole in reference to Chremistica seminiger (Distant, 1909), only known from southern India, and not Chremistica tridentigera sensu stricto Breddin (1905), which is known from Banguey island and Kalimantan, Borneo.

#### Chremistica viridis

(Fabricius, 1803)

Tettigonia viridis Fabricius, 1803

##### Materials

- **Type status:** Holotype. **Taxon:** scientificName: Chremistica viridis (Fabricius, 1803); **Location:** continent: South America; locality: Locality unknown; **Record Level:** basisOfRecord: PreservedSpecimen

##### Distribution

[Metcalf, 1963] Kedah; South America [error]; America [error]; Java; Philippine Islands; Sumatra; Surinam; Ceylon; Indochina; Siam; Malay Peninsula. [Sanborn, 2014] Vietnam, Southeast Asia, Thailand, Philippine Republic, Indonesia, Malaysia, Indochina, Sri Lanka.

##### Notes

Authority: Fabricius 1803; Type locality listed as "America meridionali" [South America], however Metcalf (1963) states this was an error. Not from Sri Lanka: Errors synonymizing Chremistica viridis (Fabricius, 1803), with Cicada atrovirens Guérin-Méneville, 1838 and Chremistica mixta (Kirby, 1891) by Distant (1892a), of which only Chremistica mixta (Kirby, 1891) is from Sri Lanka.

#### Cicada complex

Walker, 1850

Cicada complex Walker, 1850

##### Materials

- **Type status:** Holotype. **Occurrence:** sex: male; **Taxon:** scientificName: Cicada complex Walker, 1850; **Location:** locality: Locality unknown; **Record Level:** basisOfRecord: PreservedSpecimen

##### Distribution

[Metcalf, 1963] Locality Unknown; India.

##### Notes

Authority: Walker 1850; Not from India: Metcalf (1963) incorrectly stated India in reference to Dohrn (1859) who listed it with an unknown locality. The species was described by Walker (1850) without a known locality. The type specimen is not in the NHMUK and this species has not been mentioned in literature since Distant (1906a). This species was not included in the Fauna of British India by Distant (1906c).

#### Cicada orni

Linnaeus, 1758

Cicada orni Linnaeus, 1758
Cicada pallida var. a Petagna, 1787
Tettigonia punctata Fabricius, 1798
Tettigonia orni var. b Billberg, 1820 (nom. nud.)
Macroprotopus oleae Costa, 1877
Cicada orni lesbosiensis Boulard, 2000

##### Materials

- **Type status:** Holotype. **Taxon:** scientificName: Cicada orni Linnaeus, 1758; **Location:** continent: Europe; country: Italy; locality: Italia; **Record Level:** basisOfRecord: PreservedSpecimen

##### Distribution

[Metcalf, 1963] Europe. [Sanborn, 2014] France, Romania, Spain, Italy, Europe, Iran, Turkey, Albania, Austria, Czechoslovakia, Egypt, Germany, Greece, Hungary, Israel, Jordan, Lebanon, Switzerland, Spain, Balearic Island, Tunisia, USSR, Yugoslavia, Albania, Cyprus, Czechoslovakia, Crimea, Bulgaria, Turkmenistan, North Africa, Portugal, Armenia, Azerbaijan, Georgia, Turkmenia, Croatia, Mediterranean, Lesbos Island, Macedonia, Corsica, Caucasus, Slovenia, Istria, Dalmatia, Iberian Peninsula, Ukraine, Middle East, Western Asia, Southern Europe.

##### Notes

Authority: Linnaeus 1758; Not from India: While this species has a wide distribution across Europe and the Mediterranean region, the isolated records from "India" (Goeze 1778) and "Africa", are assumed to be errors until proven otherwise.

#### Cryptotympana acuta

(Signoret, 1849)

Cicada acuta Signoret, 1849
Cicada vicina Signoret, 1849
Fidicina nivifera Walker, 1850
Fidicina bicolor Walker, 1852

##### Materials

- **Type status:** Holotype. **Occurrence:** recordedBy: Patrie; sex: male; **Taxon:** scientificName: Cryptotympana acuta (Signoret, 1849); **Location:** continent: Asia; country: Indonesia; locality: Java; **Record Level:** basisOfRecord: PreservedSpecimen
- **Type status:** Other material. **Occurrence:** recordedBy: Z.O. Preanger R.; individualCount: 1; sex: male; **Taxon:** scientificName: Cryptotympana acuta (Signoret, 1849); **Location:** continent: Asia; country: Indonesia; locality: Soekapoerakolot; **Event:** eventDate: ??/03-05/1899; **Record Level:** institutionCode: NHMUK; basisOfRecord: PreservedSpecimen

##### Distribution

[Distant, 1889/92] Continental India: "Bhutan Duars ". [Metcalf, 1963] Java; Assam; East Bengal; China; Philippine Islands; Timor; India; Sumatra; Borneo; Hong Kong Island; Lombok; Bhutan; Malay States; Palawan; Malaya; Bengal; Sulu Islands; Moluccas; Amboina; Sumbawa; British India; Kwangtung; North Borneo (?); India (?); Sunda Islands; Negros. [Sanborn, 2014] China, Guangzhou, Philippines, Kalimantan, Timor, Indonesia, Malaysia, Bhutan, India, Banda Islands, Java, Bali, Malay Peninsula, Lesser Sunda Islands, Canton, Bhutan, Lonbok, Timor.

##### Notes

Authority: Signoret 1849; Not from India: Hayashi (1987a) states this species is restricted to Java and Bali. Signoret (1849) [translated] states: "The Museum has a copy with the label of Montevideo, but I think there is a mistake".

#### Cryptotympana atrata

(Fabricius, 1775)

Tettigonia atrata Fabricius, 1775
Tettigonia pustulata Fabricius, 1787
Cicada nigra Olivier, 1790
Fidicina bubo Walker, 1850
Cryptotympana sinensis Distant, 1887
Cryptotympana dubia Haupt, 1917
Cryptotympana coreanus Kato, 1925
Cryptotympana santoshonis Matsumura, 1927
Cryptotympana wenchewensis Ouchi, 1938
Cryptotypmana pustulata castanea Liu, 1940
Cryptotympana pustulata fukienensis Liu, 1940

##### Materials

- **Type status:** Holotype. **Taxon:** scientificName: Cryptotympana atrata (Fabricius, 1775); **Location:** continent: Asia; country: China; **Record Level:** basisOfRecord: PreservedSpecimen

##### Distribution

[Metcalf, 1963] China; South America [error]; Java; Hupeh; Hong Kong Island; Philippine Islands; India; Japan; Shantung; Australia; Malay Archipelago; Australia (?); Northern China; Java (?); Formosa; Hopeh; Oriental Region; Kwangtung; Malaya; Tonkin (?); Annam (?); Kiangsu; Chekiang; Hainan Island; Jehol; Manchoukuo; Southern China; Honan; Eastern China; Anhwel; Fukien; Shansi; Eastern Asia. [Sanborn, 2014] Korea, China, Tsingtau, Peiping, Lushan, Canton, Hongkong, Soochow, Hangchow, Malaysia, Australia, Formosa, Japan, Santosho, Shantung, Taiwan, Indo-China, Hunan, Macao, Hong Kong, Guangdong, Hebei, Zhejiang, Yunnan, Liaoning, Inner Mongolia, Shaanxi, Shandong, Shatosho, Anhui, Hebei, Henan, Jiangsu, Jiangxi, Zhejiang, Fujian, Hainan, Guizhou, Guangxi, Sichuan, Laos, Vietnam, Philippines, Shandong, Zhejiang, Jiangsu, Southeast Asia, Thailand.

##### Notes

Authority: Fabricius 1775; Not from India: Atkinson (1886) stated "but likely found in India" but no records have been made to specimens from India. Metcalf (1963) also report Cryptotympana bubo (Walker, 1850), a junior synonym of C. atrata, as from India according to Atkinson (1886) but this was a typo by Metcalf.

#### Cryptotympana facialis facialis

(Walker, 1858)

Cicada facialis Walker, 1858
Cryptotympana facialis formosana Kato, 1925
Cryptotympana japonensis Kato, 1925
Cryptotympana japonensis riukiuensis Kato, 1925
Cryptotympana okinawana Matsumura, 1927
Cryptotympana facialis yonakunina Ishihara, 1968

##### Materials

- **Type status:** Syntype. **Occurrence:** individualCount: 3; sex: male; **Taxon:** scientificName: Cryptotympana facialis (Walker, 1858); **Location:** continent: Asia; country: Thailand; locality: Siam; **Record Level:** basisOfRecord: PreservedSpecimen

##### Distribution

[Metcalf, 1963] Siam; China; Japan; Northern China; Shantung; Ryukyu Islands; Java; Palaearctic China; Oriental Region; Loochoo Islands; Formosa; Kansu; Thailand; Honshu; Okinawa; Kyushu. [Duffels and van der Laan, 1985] Ryukyu Islands; Kuroshima Island; Japan. [Sanborn, 2014] Japan, Tsushima Island, Goto Islands, Koshiki Islands, Osumi Islands, Tokara Islands, Ryukyus, Taiwan, China, Tsinan, Formosa, Siam, Santosho, Liu-kiu Island, Indo-China, Thailand, Shandong, Liaoning, Hebei, Henan, Shandong, Anhui, Jianxi, Zhejiang, Guangxi.

##### Notes

Authority: Walker 1858b; Unlikely to be from India: Incorrectly listed in India by Matsumura (1907) and Matsumura (1917). According to Metcalf (1963) Matsumura referred to a specimen misidentified as Cryptotympana intermedia (actually Cryptotympana japonensis Kato, 1925) and likely stated the locality information associated with C. intermedia and not C. japonensis.

#### Cryptotympana holsti holsti

Distant, 1904

Cryptotympana holsti Distant, 1904
Cryptotympana vitalisi Distant, 1917
Cryptotympana fusca Kato, 1925
Cryptotympana capillata Kato, 1925
Cryptotympana holsti inornata Matsumura, 1927
Cryptotympana kagiana Matsumura, 1927

##### Materials

- **Type status:** Holotype. **Occurrence:** sex: male; **Taxon:** scientificName: Cryptotympana holsti holsti Distant, 1904; **Location:** continent: Asia; country: Taiwan; locality: Central Formosa; **Record Level:** institutionCode: NHMUK; basisOfRecord: PreservedSpecimen

##### Distribution

[Metcalf, 1963] Formosa, Japan, China, Indochina, Southern China, Annam, Jehol, Tonkin. [Duffels and van der Laan, 1985] Taiwan, Indo-China, China. [Sanborn, 2014] Formosa, Taiwan, China, Indo-China, Vietnam, Cambodia, Laos, Fujian, Taiwan, Guangdong, Hunan, Guangxi, Guizhou, Sichuan, Yunnan, India, Hainan, Liudau Island, South China.

##### Notes

Authority: Distant 1904a; Not from India: Sanborn (2014) stated India in reference to Hua (2000). There are no other records which suggest India, leading us to believe that there has been a mistranslation of Indo-China (Vietnam) to mean India and China. Further evidence is required before this species can be included in the fauna of this region.

#### Cryptotympana takasagona

Kato, 1925

Cryptotympana takasagona Kato, 1925
Cryptotympana intermedia Schumacher, 1915 (nec Signoret, 1849)
Cryptotympana argenteus Kato, 1925

##### Materials

- **Type status:** Holotype. **Taxon:** scientificName: Cryptotympana takasagona Kato, 1925; **Location:** continent: Asia; country: Taiwan; locality: Locality unknown; **Record Level:** basisOfRecord: PreservedSpecimen

##### Distribution

[Metcalf, 1963] Formosa; India; Ceylon; Java; China; Ryukyu Islands; Central Japan; Japan; Jehol. [Sanborn, 2014] China, Fujian, Taiwan, Japan.

##### Notes

Authority: Kato 1925; Not from India: Metcalf (1963) stated "India, Ceylon" in reference to Schumacher 1915 who listed the "wide distribution" of Cryptotympana intermedia. Kato (1925) subsequently described this as a new species - C. takasagona, which has not been recorded on the Indian subcontinent.

#### Cryptotympana varicolor

Distant, 1904

Cryptotympana varicolor Distant, 1904
Cryptotympana sumbawensis Jacobi, 1941

##### Materials

- **Type status:** Holotype. **Occurrence:** sex: female; **Taxon:** scientificName: Cryptotympana varicolor Distant, 1904; **Location:** continent: Asia; country: Indonesia; locality: Sumbawa Island; **Record Level:** institutionCode: MNHN; basisOfRecord: PreservedSpecimen
- **Type status:** Other material. **Occurrence:** individualCount: 1; sex: female; **Taxon:** scientificName: Cryptotympana varicolor Distant, 1904; **Location:** continent: Asia; country: Indonesia; locality: Sumbawa; **Record Level:** institutionCode: NHMUK; basisOfRecord: PreservedSpecimen

##### Distribution

[Metcalf, 1963] Sumbawa; Ceylon; Palawan. [Duffels and van der Laan, 1985] Ceylon. [Sanborn, 2014] Sumatra, Sumbawa, Lesser Sunda Islands, Sumbawa.

##### Notes

Authority: Distant 1904c; Not from Sri Lanka: Hayashi (1987a) states "In BM, there is a male specimen of varicolor collected from Ceylon bearing the "Type" label and Distant's determination; this is not identical to varicolor Distant but with exalbida, as discussed in part 1 of this paper." Subsequent records in Duffels and van der Laan (1985), from Pringle (1955) are as a result of this misidentification.

#### Diceropyga obtecta

(Fabricius, 1803)

Tettigonia obtecta Fabricius, 1803
Dundubia bicaudata Walker, 1858

##### Materials

- **Type status:** Holotype. **Taxon:** scientificName: Diceropyga obtecta (Fabricius, 1803); **Location:** continent: Asia; country: Indonesia; locality: Amboina; **Record Level:** basisOfRecord: PreservedSpecimen

##### Distribution

[Metcalf, 1963] Amboina; Ceram; India; Sula Island; Aru Islands; Batjan; Gilolo; Ternate; Northern India; Northern Bengal; Nepal; Assam; Sumatra; New Guinea; Salawati; Duke of York Islands (Bismark Archipelago); Malay Archipelago; Obi Islands; New Britain; Lifu; Halmahera Island; Papua; Dutch New Guinea; Queensland; Northern Queensland; Buru; Southern New Guinea; Netherlands New Guinea; Aroe Islands; Territory of Papua; Admiralty Islands; Molucca Islands. [Sanborn, 2014] Moluccas, Waigeo, Gebe Island, Maluku, Selatan, Buru, Seram, Ambon, Sula, Molucca Islands, South Maluku.

##### Notes

Authority: Fabricius 1803; Not from India: Metcalf (1963) listed "India, N. India, N. Bengal, Nepal, Assam" in reference to Atkinson (1884), however this record stems from a misidentification by Atkinson.

#### Hamza ciliaris

(Linnaeus, 1758)

Cicada ciliaris Linnaeus, 1758
Cicada ocellata De Geer, 1773
Cicada varia Olivier, 1790
Tettigonia marmorata Fabricius, 1803
Platypleura arcuata Walker, 1858
Platypleura catocaloides Walker, 1870
Platypleura bouruensis Distant, 1898
Platypleura lyricen Kirkaldy, 1913
Hamza uchiyamae Matsumura, 1927

##### Materials

- **Type status:** Holotype. **Taxon:** scientificName: Hamza ciliaris (Linnaeus, 1758); **Location:** continent: Asia; locality: Indiis; **Record Level:** basisOfRecord: PreservedSpecimen

##### Distribution

[Metcalf, 1963] India; Sweden; South America; Tranquebar; Cape of Good Hope; Amboina; Natal; Ceram [?]; Amboina [?]; Ternate; Philippine Islands; Ceram; Morty; South Africa; Java; China; Molucca Islands; Cochin China; Indo-Australian Archipelago; Malay Archipelago; Indo-China. [Sanborn, 2014] Indo-China, Banda, Ambione, Indonesia, Buru, Buru Island, Caroline Islands, Palau Islands, Ambon, Ambiona, Morotai, Ceram, Saparau, Anguar Island, Mindanao, Molucca Islands, Maluku, Banda Islands, Timor,Kai Island, South Maluku, Sula Islands, North Maluku,Vietnam, Philippines, Malayian Archipelago, India, Panay, Philippines, Palau, Indonesia, Banggai Archipelago.

##### Notes

Authority: Linnaeus 1758; Not from India: Metcalf (1963) stated India in reference to the type specimen location that was listed as "Indiis". Duffels (1991) states that this species is from Indonesia, and that the Indian listing by Metcalf is eronious. Thus subsequent references to India in Sanborn (2014) are likely to be due to previous inaccurate literature localities.

#### Huechys incarnata incarnata

(Germar, 1834)

Cicada incarnata Germar, 1834
Cicada sanguinolenta Brulle, 1835 (nec Fabricius, 1775)
Cicada germari Guerin-Meneville, 1838

##### Materials

- **Type status:** Holotype. **Occurrence:** recordedBy: de Haan; **Taxon:** scientificName: Huechys incarnata incarnata (Germar, 1834); **Location:** continent: Asia; locality: India orientali; **Record Level:** basisOfRecord: PreservedSpecimen

##### Distribution

[Metcalf, 1963] India; Java; Asia; Locality Unknown; Molucca Islands; Sumatra; Amboina; Celebes; Malay Archipelago; Java (?); Sumbawa. [Sanborn, 2014] Sulawesi, West Melanesia.

##### Notes

Authority: Germar 1834; Unlikely to be from India: Metcalf (1963) states "India", however the species description states "India orientali" with no specific location listed. As all subsequent specimens have been recorded from the Indonesian region.

#### Huechys phaenicura

(Germar, 1834)

Cicada phaenicura Germar, 1834
Huechys phaenicura balabakensis Haupt, 1924
Huechys phaenicura palawanensis Haupt, 1924

##### Materials

- **Type status:** Holotype. **Taxon:** scientificName: Huechys phaenicura (Germar, 1834); **Location:** continent: Asia; locality: India orientali; **Record Level:** basisOfRecord: PreservedSpecimen

##### Distribution

[Metcalf, 1963] India; Asia; Locality Unknown; Eastern India; Philippine Islands; Java; Malay Archipelago; Sikkim; Luzon; Samar. [Sanborn, 2014] Philippines, Palawan, Samar, Luzon, Balabac Island, Malaysia, Indonesia, Java, India, Laos, Brazil, Mindanao, Samar.

##### Notes

Authority: Germar 1834; Unlikley to be from India: Metcalf (1963) states "India", "Eastern India" and "Sikkim", however the species description states "India orientali" with no specific location listed. Furthermore, Distant (1892a) states that "According to Mr. Atkinson this species is "reported from India, Sikkim," but at present it seems extremely doubtful that it is found on the Continent at all. I certainly have seen it in none of the many collections examined from India, Burma, Tenasserim, or the Malay Peninsula, and it is probably an insular species". Thus the subsequent "India" locality in Sanborn (2014), in reference to Lee (2009a), is likely to be a continuation of this earlier error.

#### Macrosemia juno

(Distant, 1905)

Platylomia juno Distant, 1905

##### Materials

- **Type status:** Holotype. **Occurrence:** recordedBy: R.P. Gros-Jean; sex: male; **Taxon:** scientificName: Macrosemia juno (Distant, 1905); **Location:** continent: Asia; country: China; locality: Ta-tsien-lou, Se-Tchouen; **Record Level:** institutionCode: MNHN; basisOfRecord: PreservedSpecimen

##### Distribution

[Metcalf, 1963] Szechwan, China, Se-Tchouen, Central China [Duffels, 1985] China [Sanborn, 2014] China, Szechuan, Hunan, Zhejiang, Fujian, Taiwan, Guangdong, Guangxi, Sichuan, Xizang, India, Malaysia.

##### Notes

Authority: Distant 1905c; Not from India: Sanborn (2014) stated India in reference to Hua (2000). There are no other records that suggest India, leading us to believe that there has been a mistranslation of Indo-China (Vietnam) to mean India and China.

#### Megapomponia imperatoria

(Westwood, 1842)

Cicada imperatoria Westwood, 1842

##### Materials

- **Type status:** Holotype. **Taxon:** scientificName: Megapomponia imperatoria (Westwood, 1842); **Location:** continent: Asia; locality: East Indies and the Indian Islands; **Record Level:** institutionCode: OUMNH; basisOfRecord: PreservedSpecimen

##### Distribution

[Metcalf, 1963] East Indies; Indian Islands; Nepal; India; Assam; Malaya; Borneo; Sumatra; Penang; Cambodia; Laos; Siam; Malay Peninsula; Java; Malay Islands; Malay States; Sarawak; Malacca; Perak; Singapore Island; Johore. [Duffels and van der Laan, 1985] Nepal. [Sanborn, 2014] Thailand, India, Nepal, Malaysia, Borneo, Cambodia, Laos, Sarawak, Indonesia, Indian Islands, Peninsular Malaysia.

##### Notes

Authority: Westwood 1842; Multiple localities listed in species description. Not from India: Metcalf (1963), in error, listed "India, Assam, Nepal" in reference to Walker (1850). The type description does not give a specific locality (East Indies and the Indian Islands), while recent literature indicates that this species is confined to the Malaysian Peninsula (Lee 2014). Subsequent references to India (and Nepal) in Metcalf (1963), Duffels and van der Laan (1985) and Sanborn (2014) are likely to be based on this previous error.

#### Mogannia hebes

(Walker, 1858)

Cephaloxys hebes Walker, 1858
Mogannia spurcata Walker, 1858
Mogannia hebes var. a Kato, 1925
Mogannia hebes var. b Kato, 1925
Mogannia hebes var. c Kato, 1925
Mogannia flavescens Kato, 1925
Mogannia delta Kato, 1925
Mogannia hebes var. d Kato, 1925
Mogannia ritozana Matsumura, 1927
Mogannia ritozana dorsovitta Matsumura, 1927
Mogannia katonis Matsumura, 1927
Mogannia subfusca Kato, 1928
Mogannia hebes concolor Kato, 1932
Mogannia janea Hua, 2000

##### Materials

- **Type status:** Holotype. **Occurrence:** sex: male; **Taxon:** scientificName: Mogannia hebes (Walker, 1858); **Location:** continent: Asia; country: China; locality: North China; **Record Level:** basisOfRecord: PreservedSpecimen

##### Distribution

[Metcalf, 1963] Northern China; China; Formosa; Tonkin; Ryukyu Islands; Hong Kong Island; Malaya; India; Japan; Indochina; Korea; Java; Kwangtung; Kotosho Island; Fukien; Kiangsu; Anhwei; Chekiang; Manchuria; Chusan Island; Eastern China; Kwangsi; Szechwan; Sikang. [Sanborn, 2014] China, Manchuria, Nanking, Soochow, Chekiang, Foochow, Amoy, Canton, Hongkong, Hangchow, Korea, Formosa, Taiwan, Hunan, Zhejiang, Macao, Korea, India, Myanmar, China, Chejiang, Fujian, Guangdong, Guangxi, Jiangsi, Sichuan, Yunnan, Inner Mongolia, Henan, Hubei, Anhui, Jiangsu, Jiangxi, Zhejiang, Fujian, Taiwan, Guangdong, Hong Kong, Sichuan, Vietnam, Taiwan, Southeast Asia, Liudau Island, Lanya Island, Fujian.

##### Notes

Authority: Walker 1858b; Unlikely to be from India: Matsumura (1910) stated this species to be from "Indien" but no specimens have been recorded from this region.

#### Nabalua mascula

(Distant, 1889)

Leptopsaltria mascula Distant, 1889

##### Materials

- **Type status:** Holotype. **Occurrence:** sex: male; **Taxon:** scientificName: Nabalua mascula (Distant, 1889); **Location:** continent: Asia; country: Malaysia; locality: Kina Balu Mountain, North Borneo; **Record Level:** basisOfRecord: PreservedSpecimen

##### Distribution

[Metcalf, 1963] North Borneo; Borneo; Johore; Malay Peninsula; India. [Sanborn, 2014] Borneo, Sarawak, Sabah, Johore.

##### Notes

Authority: Distant 1889c; Unlikely to be from India: Only Kato (1932) listed India as a locality, without reference to an examined specimen. All other references list Borneo, Johore and Malay Peninsula.

#### Platypleura stridula

(Linnaeus, 1758)

Cicada stridula Linnaeus, 1758
Cicada catenata Drury, 1773
Cicada nigrolinea De Geer, 1773
Platypleura stridula var. a Stål, 1866

##### Materials

- **Type status:** Lectotype. **Occurrence:** individualCount: 1; **Taxon:** scientificName: Platypleura stridula (Linnaeus, 1758); **Location:** continent: Asia; locality: Indiis; **Record Level:** institutionCode: UUZM; basisOfRecord: PreservedSpecimen

##### Distribution

[Metcalf, 1963] India; Cape of Good Hope; South Africa; Natal; Transvaal; Africa. [Sanborn, 2014] South Africa, Zambezi, Zambia, Australia.

##### Notes

Authority: Linnaeus 1758; Not from India: Type locality stated as "Indiis", which was reported as India in Metcalf (1963), however all other specimens have been collected from southern Africa.

#### Platypleura takasagona

Matsumura, 1917

Platypleura takasagona Matsumura, 1917

##### Materials

- **Type status:** Syntype. **Occurrence:** individualCount: 2; sex: male; **Taxon:** scientificName: Platypleura takasagona Matsumura, 1917; **Location:** continent: Asia; country: Taiwan; locality: Kiirun, Formosa; **Record Level:** institutionCode: EIHU; basisOfRecord: PreservedSpecimen

##### Distribution

[Metcalf, 1963] Formosa; Britih India; Assam; Burma; China; Japan; Hyukyu Islands; Honshu. [Sanborn, 2014] China, Taiwan.

##### Notes

Authority: Matsumura 1917; Unlikely to be from India: Incorrectly listed in India and Burma by Schumacher (1915). According to Metcalf (1963), Schumacher referred to a specimen misidentified as Pycna repanda (actually Platypleura takasagona) and likely stated the locality information associated with P. repanda and not P. takasagona.

#### Platypleura testacea

(Walker, 1858)

Zammara testacea Walker, 1858

##### Materials

- **Type status:** Holotype. **Occurrence:** catalogNumber: BMNH(E) 1009498; individualCount: 1; sex: female; **Taxon:** scientificName: Platypleura testacea (Walker, 1858); **Location:** locality: Locality unknown; **Record Level:** institutionCode: NHMUK; basisOfRecord: PreservedSpecimen

##### Distribution

[Metcalf, 1963] Oman; Iran; India. [Sanborn, 2014] Iran, India, Oman.

##### Notes

Authority: Walker 1858a; Unsure of number of specimens used in description, however only one female specimen in the NHMUK collection. Not from India: Myers (1928) claimed the locality of this species to be "India (locality unknown)" which is incorrect as it is restricted to the Arabian penninsula. Subsequent reference to India in Sanborn (2014) by Mozaffarian and Sanborn (2010) likely due to previous inaccurate literature.

#### Pomponia adusta

(Walker, 1850)

Cicada adusta Walker, 1850
Cicada buddha Kirkaldy, 1909

##### Materials

- **Type status:** Holotype. **Occurrence:** sex: male; **Taxon:** scientificName: Pomponia adusta (Walker, 1850); **Location:** continent: Asia; country: Indonesia; locality: Java; **Record Level:** basisOfRecord: PreservedSpecimen

##### Distribution

[Metcalf, 1963] Java; Siam; Malay Peninsula; Borneo; Sumatra; Burma; Assam. [Sanborn, 2014] Malaysia, Langkawi Island, Java, Sumatra, Borneo, Peninsular Malaysia, Siam.

##### Notes

Authority: Walker 1850; Not from India: Metcalf (1963) incorrectly listed the species from Burma and Assam in reference to Moulton (1929) who was suggesting possible distributions, however the species has been recorded from Malaysia and Indonesia.

#### Scieroptera formosana

Schmidt, 1918

Scieroptera formosana Schmidt, 1918
Scieroptera formosana ater Kato, 1925
Scieroptera formosana trigutta Kato, 1926
Scieroptera formosana albifascia Kato, 1926

##### Materials

- **Type status:** Holotype. **Occurrence:** recordedBy: S. Sauter; individualCount: 1; sex: female; **Taxon:** scientificName: Scieroptera formosana Schmidt, 1918; **Location:** continent: Asia; country: Taiwan; locality: Alikang, Formosa; **Event:** eventDate: ??/06/??; **Record Level:** institutionCode: MZPW; basisOfRecord: PreservedSpecimen

##### Distribution

[Metcalf, 1963] Formosa; Japan; China; Philippine Islands; Malay Archipelago; Borneo; Java; India; Burma; Celebes; Chekiang; Eastern China. [Sanborn, 2014] China, Schumacher China, Jiangxi, Zhejiang, Fujian, Taiwan, Guangdong, Hubei, Hunan, Guangxi, Sichuan, India, Burma, Philippines, Malaysia, Taiwan, Vietnam, Southeast Asia.

##### Notes

Authority: Schmidt 1918; Unlikely to be from India: Incorrectly listed in India and Burma by Matsumura (1917). According to Metcalf (1963) Matsumura referred to a specimen misidentified as Scieroptera formosana (but in fact Scieroptera splendidula) and likely stated the locality information associated with S. splendidula and not S. formosana.

#### Scieroptera sumatrana sumatrana

Schmidt, 1918

Scieroptera sumatrana Schmidt, 1918

##### Materials

- **Type status:** Syntype. **Occurrence:** recordedBy: Dr H. Dohrn; sex: male; **Taxon:** scientificName: Scieroptera sumatrana sumatrana Schmidt, 1918; **Location:** continent: Asia; country: Indonesia; locality: Soekaranda, Sumatra; **Event:** eventDate: ??/01/1894; **Record Level:** institutionCode: MZPW; basisOfRecord: PreservedSpecimen
- **Type status:** Syntype. **Occurrence:** recordedBy: Dr H. Dohrn; sex: female; **Taxon:** scientificName: Scieroptera sumatrana sumatrana Schmidt, 1918; **Location:** continent: Asia; country: Indonesia; locality: Soekaranda, Sumatra; **Event:** eventDate: ??/01/1894; **Record Level:** institutionCode: MZPW; basisOfRecord: PreservedSpecimen

##### Distribution

[Metcalf, 1963] Sumatra (Soekaranda); China; Malay Archipelago; Sarawak; Northern India; Assam; Tenasserim; India; Burma; Java; Borneo; Celebes; Oriental Region; Formosa; Indochina; Nias; Malay Peninsula; Celebes (?). [Sanborn, 2014] Vietnam, Malaysia, Indonesia, Java, Sumatra, Myanmar, India.

##### Notes

Authority: Schmidt 1918; Unlikely to be from India and Burma: Incorrectly listed in India and Burma by Moulton (1923). According to Metcalf (1963) Moulton referred to a specimen misidentified as Scieroptera splendidula (actually Scieroptera sumatrana) and likely stated the locality information associated with *S. splendidula* and not *S. sumatrana*.

#### Tacua speciosa speciosa

(Illiger, 1800)

Tettigonia speciosa Illiger, 1800
Cicada indica Donovan, 1800
Tettigonia gigantea Weber, 1801

##### Materials

- **Type status:** Holotype. **Taxon:** scientificName: Tacua speciosa speciosa (Illiger, 1800); **Location:** continent: Asia; country: Indonesia; locality: Sumatra; **Record Level:** basisOfRecord: PreservedSpecimen

##### Distribution

[Metcalf, 1963] Bengal; Sumatra; India; Java; Asia; Borneo; Hindustan; Europe; Eastern India; Sunda Islands; Penang; Sarawak; Malay States; Malaya; Malacca; Singapore Island; Perak; Johore; East Indies (?); Bengal (?); Malay Peninsula. [Sanborn, 2014] Malaysia, Borneo, Sarawak, Sumatra, Java, Sabah, Peninsular Malaysia, Malausia, Indonesia.

##### Notes

Authority: Illiger 1800; Unlikely to be from India: Metcalf (1963), among others, states India in reference to the type locality of Cicada indica Donovan, 1800 which is a junior synonym of Tacua speciosa speciosa (Illiger, 1800). Distant (1892a), however noted that "According to Donovan, a single specimen of this species was found in Bengal by Mr. Fichtel, and deposited in the Imperial Cabinet at Vienna, but that habitat I consider liable to the greatest doubt." Cicada indica is illustrated in Fig. 1.

#### Tanna japonensis japonensis

(Distant, 1892)

Pomponia japonensis Distant, 1892
Leptopsaltria japonica Horvath, 1892
Tanna (?) *sasaii* Matsumura, 1939
Tanna japonensis kimotoi Kato, 1943
Tanna japonensis nigrofusca Kato, 1940
Tanna obliqua Liu, 1940

##### Materials

- **Type status:** Syntype. **Occurrence:** recordedBy: Pryer; sex: male; **Taxon:** scientificName: Tanna japonensis japonensis (Distant, 1892); **Location:** continent: Asia; country: Japan; **Record Level:** basisOfRecord: PreservedSpecimen
- **Type status:** Syntype. **Occurrence:** sex: male; **Taxon:** scientificName: Tanna japonensis japonensis (Distant, 1892); **Location:** continent: Asia; country: Japan; locality: Tokoe; **Record Level:** basisOfRecord: PreservedSpecimen

##### Distribution

[Metcalf, 1963] Japan; Laos; Hokkaido; Honshu; Kyushu; Shikokll; India; Malay Archipelago; Quelpart Island; Korea; Southern Manchuria; Manchuria; China; Kwantung; Central Japan; Chekiang; Awaji-shima; Formosa. [Sanborn, 2014] China, Manchuria, Hangchow, Japan, Korea, Hunan, Laos, Guangxi, Sichuan, Xizang, Querpart Islands, India, Anhui, Hubei, Jiangxi, Zhejiang, Fujian.

##### Notes

Authority: Distant 1892c; Not from India: Distant (1916) listed the species (without a specimen) from India but no subsequent specimens have been collected from India. Subsequent listing of "India" in Sanborn (2014) in reference to Hua (2000) may have been a subsequent error from Distant (1916) and Metcalf (1963).

#### Terpnosia psecas

(Walker, 1850)

Dundubia psecas Walker, 1850

##### Materials

- **Type status:** Holotype. **Occurrence:** sex: female; **Taxon:** scientificName: Terpnosia psecas (Walker, 1850); **Location:** continent: Asia; country: Indonesia; locality: Java; **Record Level:** institutionCode: NHMUK; basisOfRecord: PreservedSpecimen

##### Distribution

[Metcalf, 1963] Java; Ceylon; Sikkim; Borneo; India; British India; Sumatra; Siam (?); Sunda Islands; Burma. [Duffels and van der Laan, 1985] Ceylon. [Sanborn, 2014] Java, Peninsular Malaysia, Ceylon, Borneo, Sarawak, Siam, Sabah.

##### Notes

Authority: Walker 1850; Distant (1906a) refers to a male specimen, but lists two locations. Unlikely to be from India or Sri Lanka: Lee (2012b) states that the type specimen of **Terpnosia psecas** (Walker, 1850) is a female from Java. Male specimens identified by former authors were not true *T. psecas* but were *T. elegans* (Kirby, 1891) from Sri Lanka. The possible confusion with India has not been resolved due to specimen unavailability Kato (1932), however it is highly unlikely given the reported range (Indonesia and Malaysia). Subsequent references to India and Sri Lanka in Duffels and van der Laan (1985) and Sanborn (2014) are likely in reference to these previous misidentifications.

#### Unduncus connexa

(Distant, 1910)

Lemuriana connexa Distant, 1910

##### Materials

- **Type status:** Holotype. **Occurrence:** recordedBy: Moulton; sex: female; **Taxon:** scientificName: Unduncus connexa (Distant, 1910); **Location:** continent: Asia; country: Malaysia; locality: Lawas, Sarawak; **Record Level:** basisOfRecord: PreservedSpecimen

##### Distribution

[Metcalf, 1963] Borneo; Sarawak; Ceylon; India. [Sanborn, 2014] Borneo, Sabah, Sarawak, Oriental Region.

##### Notes

Authority: Distant 1910; Unlikely to be from India: Incorrectly listed in Ceylon and India by Moulton (1912). According to Metcalf (1963) Moulton incorrectly referred to a specimen of Lemuriana connexa as Abroma nubifurca and likely stated locality information associated with A. nubifurca and not L. connexa.

#### Zouga fornicata

(Linnaeus, 1758)

Cicada fornicata Linnaeus, 1758
Cicada fornicata Olivier, 1790
Cicada dimidiata Olivier, 1790
Carineta leuconeura Walker, 1850

##### Materials

- **Type status:** Holotype. **Taxon:** scientificName: Zouga fornicata (Linnaeus, 1758); **Location:** continent: Africa; country: South Africa; locality: Indiis; **Record Level:** basisOfRecord: PreservedSpecimen

##### Distribution

[Metcalf, 1963] India; South America [?]; Cape of Good Hope; Africa; Southern Africa; South Africa. [Sanborn, 2014] South Africa.

##### Notes

Authority: Linnaeus 1758; Not from India: Type specimen recorded from "Indiis", which Metcalf 1963 reported as India, however all subsequent specimens have only been collected from southern Africa.

## Discussion

In this paper we have presented a major taxonomic update over Distant's (Distant 1906c, Distant 1916) faunistic work on cicadas of "British India", which included all of South Asia - i.e. the Indian subcontinent. The current species total for modern political India and Bangladesh stands at 189 species and increases to 256 species when including Bhutan, Myanmar, Nepal and Sri Lanka from the present study (Table 2) and Pakistan (Ahmed and Sanborn 2010). This represents a substantial increase from Distant (1916) figure of 172 species for “British India”. No doubt this modern figure will increase further as cicadas are sampled more extensively and studied more intensively using modern techniques such as acoustic and molecular analyses, unavailable in Distant's time. Many of the cicada groups may contain cryptic species that will be discovered using these methods, which is a common experience in Cicadidae (e.g. Price et al. 2007, Price et al. 2010, Popple 2013, Marshall et al. 2015, Hertach et al. 2015, Wade et al. 2015) especially in recently neglected biodiversity hotspots such as India. We hope that this current taxonomic update will facilitate species discovery and further systematic and ecological work on cicadas of the Indian region.

The cicada fauna inventoried above for the Indian region represents approximately 17% of the currently recognized global cicada genera (73 of 434 genera: data derived from Sanborn 2014). At a country level the generic diversity of India (including Bangladesh) ranks highest in the world (64 of 434 genera), followed by China (61 of 434 genera). The species diversity also compares favourably with that of other countries in the region which have recently been studied (Table 3). Of the 30 species currently recorded from Pakistan (Ahmed and Sanborn 2010, Ahmed et al. 2010) 21 are recorded from India, while 9 are either not present or yet to be recorded. Although India has larger landmass compared to most of its neighboring countries, the biogeographic placement of modern India and the topographic and climatic diversity of its bioregions may have contributed more to its rich cicada fauna than the size of the landmass. However, this needs to be explored further with phylogenetic / phylogeographic studies.

India is rapidly developing and expanding its scientific infrastructure and personnel, and investing heavily in scientific research. There is also growing interest in biodiversity studies and conservation among professional biologists and citizen scientists, which will help in exploration and mapping of the current state of Indian biodiversity. As a result, we expect rapid scientific developments involving cicadas of India. To facilitate this development, we have launched a website on Indian cicadas (http://www.indiancicadas.org/), which will act as a central repository of reference images representing spot records, and various species attributes such as cicada songs and other phenotypic variation. Acoustic recordings of Indian cicadas may also be found at: http://bio.acousti.ca/). Following the publication of this paper, we aim to provide newly generated taxonomic and biological information on Indian cicadas through the Cicadas of India website, where updated information may be readily accessed in a centralised database.

## Figures and Tables

**Table 1.** Summary of institutional abbreviations used in the checklists. Data sourced from Evenhuis (2015).

**Abbreviation**	**Institution**
AMS	Australian Museum, Sydney, Australia
BPBM	Bernice P. Bishop Museum, Honolulu, USA
CAS	California Academy of Sciences, San Francisco, California, USA
CNMS	National Museum, Colombo, Sri Lanka
EIHU	Hokkaido University, Sapporo, Hokkaido, Japan
ERC-ZSI	Regional Museum of Eastern Regional Centre, Zoological Survey of India, Shillong Meghalaya, India
IFRI	Indian Forest Research Institute, Dehra Dun, Uttar Pradesh, India
IRSNB	Institut Royal des Sciences Naturelles de Belgique, Brussels, Belgium
IZUI	Institut für Zoologie der Universitat Innsbruck, Innsbruck, Austria
MCZ	Museum of Comparative Zoology, Harvard University, Cambridge, Massachusetts, USA
MNHN	Muséum National d'Histoire Naturelle, Paris, France
MSNG	Museo Civico di Storia Naturale di Genova
MZH	Finnish Museum of Natural History, Helsinki, Finland
MZLU	Museum of Zoology, Lund University, Lund, Sweden
MZPW	Museum and Institute of Zoology, Polish Academy of Science, Warszawa, Poland
NHMK	Natural History Museum, University of Karachi, Karachi, Pakistan
NHMUK	Natural History Museum, London, UK (formerly BMNH)
NHMW	Naturhistorisches Museum Wien, Austria
NHRS	Naturhistoriska Riksmuseet, Stockholm, Sweden
NSMT	National Science Museum (Natural History), Tokyo, Japan
NZSI	National Zoological Collection, Zoological Survey of India, Kolkata, India
OUMNH	University Museum of Natural History, Oxford, UK
SEMC	Snow Entomological Museum, University of Kansas, Lawrence, Kansas, USA
UUZM	Uppsala University, Uppsala, Sweden
ZMAN	Zoologisch Museum, Instituut voor Taxonomische Zoologie, Universiteit van Amsterdam, Amsterdam, Netherlands
ZMUC	Zoological Museum, University of Copenhagen, Copenhagen, Denmark

**Table 2.** Checklist summary of the species recorded in each country of the Indian subcontinent. Bold values are totals for each grouping (tribe or subfamily).

**Species**	**India and Bangladesh**	**Bhutan**	**Myanmar**	**Nepal**	**Sri Lanka**
** CICADETTINAE **	**26**		**11**	**6**	**3**
** Carinetini **			**1**		
*Karenia ravida* Distant, 1888			x		
** Cicadettini **	**6**			**1**	**1**
*Cicadetta intermedia* (Ollenbach, 1929)	x				
*Cicadetta minuta* (Ollenbach, 1929)	x				
*Emathia aegrota* Stål, 1866	x				
*Linguacicada continuata* (Distant, 1888)	x				
*Melampsalta literata* (Distant, 1888)	x				
*Panka simulata* Distant, 1905					x
*Pauropsalta exaequata* (Distant, 1892)	x				
*Tibeta zenobia* (Distant, 1912)				x	
** Huechysini **	**13**		**9**	**2**	
*Graptotettix guttatus* Stål, 1866	x			x	
*Graptotettix thoracicus* Distant, 1892			x		
*Huechys beata* Chou et al., 1997	x				
*Huechys haematica* Distant, 1888	x		x		
*Huechys incarnata testacea* (Fabricius, 1787)	x				
*Huechys sanguinea philaemata* (Fabricius, 1803)	x		x		
*Huechys sanguinea sanguinea* (De Geer, 1773)	x		x		
*Huechys thoracica* Distant, 1879	x		x		
*Scieroptera crocea crocea* (Guérin-Méneville, 1838)	x		x		
*Scieroptera fumigata* (Stål, 1854)	x				
*Scieroptera montana* Schmidt, 1918	x				
*Scieroptera splendidula cuprea* (Walker, 1870)	x		x		
*Scieroptera splendidula splendidula* (Fabricius, 1775)	x		x	x	
*Scieroptera splendidula* var. *c* Distant, 1892	x				
*Scieroptera splendidula* var. *d* Distant, 1892			x		
** Parnisini **	**3**			**1**	
*Bijaurana sita* Distant, 1912	x				
*Bijaurana typica* Distant, 1912				x	
*Lycurgus subvittus* (Walker, 1850)	x				
*Quintilia pomponia* Distant, 1912	x				
** Taphurini **	**4**			**2**	**2**
*Abroma apicalis* Ollenbach, 1929	x				
*Abroma bengalensis* Distant, 1906	x			x	
*Abroma maculicollis* (Guérin-Méneville, 1838)	x				x
*Abroma nubifurca* (Walker, 1858)					x
*Lemuriana apicalis* (Germar, 1830)	x			x	
** Tettigomyiini **			**1**		
*Kumanga sandaracata* (Distant, 1888)			x		
** CICADINAE **	**159**	**19**	**70**	**40**	**19**
** Cicadatrini **	**23**		**6**	**2**	
*Cicadatra acberi* (Distant, 1888)	x				
*Cicadatra anoea* (Walker, 1850)	x				
*Cicadatra gingat* China, 1926	x				
*Cicadatra inconspicua* Distant, 1912	x				
*Cicadatra raja* Distant, 1906	x				
*Cicadatra sankara* (Distant, 1904)	x				
*Cicadatra walkeri* Metcalf, 1963	x				
*Cicadatra xantes* (Walker, 1850)	x				
*Mogannia aurea* Fraser, 1942	x				
*Mogannia conica conica* (Germar, 1830)	x		x	x	
*Mogannia cyanea* Walker, 1858	x		x		
*Mogannia effecta effecta* Distant, 1892	x			x	
*Mogannia effecta* var. *a* Distant, 1892	x				
*Mogannia effecta* var. *b* Distant, 1892	x				
*Mogannia funebris* Stål, 1865	x		x		
*Mogannia funebris* var. *a* Distant, 1892			x		
*Mogannia nasalis nasalis* (White, 1844)	x				
*Mogannia obliqua* Walker, 1858	x		x		
*Mogannia venutissima* var. *a* Stål, 1865	x				
*Mogannia venutissima* var. *b* Stål, 1865	x				
*Mogannia venutissima venutissima* Stål, 1865	x				
*Mogannia viridis* (Signoret, 1847)	x		x		
*Psalmocharias querula* (Pallas, 1773)	x				
*Psalmocharias rugipennis* (Walker, 1858)	x				
** Cicadini **	**38**	**5**	**12**	**13**	**8**
*Aetanna tigroides* (Walker, 1858)	x				
*Basa singularis* (Walker, 1858)	x				
*Calcagninus divaricatus* Bliven, 1964	x				
*Calcagninus nilgirensis* (Distant, 1887)	x				
*Calcagninus picturatus* (Distant, 1888)	x				
*Cicada conspurcata* (Fabricius, 1777)	x				
*Cicada olivierana* Metcalf, 1963	x				
*Euterpnosia crowfooti* (Distant, 1912)	x			x	
*Euterpnosia madhava* (Distant, 1881)	x	x			
*Gudaba maculata* Distant, 1912	x				
*Gudaba marginatus* (Distant, 1897)			x		
*Leptopsaltria andamanensis* Distant, 1888	x				
*Leptopsaltria samia* (Walker, 1850)	x				
*Leptopsaltria tuberosa* (Signoret, 1847)	x			x	
*Maua quadrituberculata tavoyana* (Ollenbach, 1929)			x		
*Tanna thalia* (Walker, 1850)	x			x	
*Paranosia andersoni* (Distant, 1892)	x	x	x	x	
*Paratanna parata* Lee, 2012	x				
*Pomponia cinctimanus* (Walker, 1850)	x				
*Pomponia cyanea* Fraser, 1948	x				
*Pomponia linearis* (Walker, 1850)	x	x	x	x	
*Pomponia picta* (Walker, 1870)	x			x	x
*Pomponia ramifera* (Walker, 1850)	x				
*Pomponia secreta* Hayashi, 1978				x	
*Pomponia solitaria* Distant, 1888	x				
*Pomponia surya* Distant, 1904	x	x			
*Pomponia urania* (Walker, 1850)	x				
*Pomponia zebra* Bliven, 1964	x				
*Purana campanula* Pringle, 1955					x
*Purana guttularis* (Walker, 1858)			x		
*Purana morrisi* (Distant, 1892)	x				
*Purana tigrina* (Walker, 1850)	x		x		
*Rustia dentivitta* (Walker, 1862)	x		x	x	
*Rustia tigrina* (Distant, 1888)	x		x		
*Tanna bhutanensis* Distant, 1912		x		x	
*Tanna minor* Hayashi, 1978				x	
*Taungia abnormis* Ollenbach, 1929			x		
*Terpnosia abdullah* Distant, 1904	x				
*Terpnosia clio* (Walker, 1850)	x		x	x	
*Terpnosia collina* (Distant, 1888)	x		x	x	
*Terpnosia confusa* Distant, 1905	x				x
*Terpnosia elegans* (Kirby, 1891)					x
*Terpnosia ganesa* Distant, 1904	x			x	
*Terpnosia jenkinsi* Distant, 1912	x				
*Terpnosia lactea* (Distant, 1887)	x				
*Terpnosia maculipes* (Walker, 1850)	x		x		
*Terpnosia polei* (Henry, 1931)					x
*Terpnosia ransonneti* (Distant, 1888)	x				x
*Terpnosia ridens* Pringle, 1955					x
*Terpnosia stipata* (Walker, 1850)					x
** Cryptotympanini **	**9**	**1**	**6**	**2**	**2**
*Chremistica germana* (Distant, 1888)			x		
*Chremistica mixta* (Kirby, 1891)					x
*Chremistica ribhoi* Hajong & Yaakop, 2013	x				
*Chremistica seminiger* (Distant, 1909)	x				
*Cryptotympana aquila* (Walker, 1850)			x		
*Cryptotympana auropilosa* Hayashi, 1987			x		
*Cryptotympana corvus* (Walker, 1850)	x	x		x	
*Cryptotympana edwardsi* Kirkaldy, 1902	x				
*Cryptotympana exalbida* Distant, 1891	x				x
*Cryptotympana insularis* Distant, 1887	x				
*Cryptotympana intermedia* (Signoret, 1849)	x		x	x	
*Cryptotympana limborgi* Distant, 1888			x		
*Cryptotympana mandarina* Distant, 1891			x		
*Cryptotympana recta* (Walker, 1850)	x				
*Cryptotympana vesta* (Distant, 1904)	x				
** Dundubiini **	**37**	**5**	**25**	**6**	**3**
*Champaka spinosa* (Fabricius, 1787)	x		x		
*Dundubia emanatura* Distant, 1889	x				
*Dundubia ensifera* Bloem & Duffels, 1976	x				
*Dundubia feae* (Distant, 1892)			x		
*Dundubia hastata* (Moulton, 1923)	x				
*Dundubia laterocurvata* Beuk, 1996	x		x		
*Dundubia myitkyinensis* Beuk, 1996			x		
*Dundubia nagarasingna* Distant, 1881	x		x		
*Dundubia oopaga* (Distant, 1881)	x		x		
*Dundubia rufivena rufivena* Walker, 1850	x		x		
*Dundubia terpsichore* (Walker, 1850)	x		x		
*Dundubia vaginata vaginata* (Fabricius, 1787)	x		x		
*Haphsa bindusara* (Distant, 1881)			x		
*Haphsa durga* (Distant, 1881)	x				
*Haphsa karenensis* Ollenbach, 1929			x		
*Haphsa nicomache* (Walker, 1850)	x			x	
*Haphsa scitula* (Distant, 1888)	x		x		
*Haphsa stellata* Lee, 2009	x				
*Khimbya cuneata* (Distant, 1897)			x		
*Khimbya diminuta* (Walker, 1850)	x		x		
*Khimbya evanescens* (Walker, 1858)	x		x		
*Khimbya immsi* Distant, 1912	x				
*Khimbya sita* (Distant, 1881)	x				
*Lethama locusta* (Walker, 1850)	x				
*Macrosemia assamensis* (Distant, 1905)	x				
*Macrosemia beaudouini* (Boulard, 2003)			x		
*Macrosemia saturata* (Walker, 1858)	x	x		x	
*Macrosemia saturata* var. *a* (Distant, 1891)	x				
*Macrosemia saturata* var. *b* (Distant, 1891)	x				
*Macrosemia tonkiniana* (Jacobi, 1905)			x		
*Macrosemia umbrata* (Distant, 1888)	x	x	x	x	
*Mata kama* (Distant, 1881)	x			x	
*Mata rama* Distant, 1912		x			
*Megapomponia intermedia* (Distant, 1905)			x		
*Meimuna cassandra* Distant, 1912	x				
*Meimuna gamameda* (Distant, 1902)					x
*Meimuna microdon* (Walker, 1850)	x				
*Meimuna pallida* Ollenbach, 1929	x				
*Meimuna silhetana* (Distant, 1888)	x				
*Meimuna tavoyana* (Distant, 1888)			x		
*Meimuna tripurasura* (Distant, 1881)	x				x
*Meimuna velitaris* (Distant, 1897)			x		
*Platylomia amicta* (Distant, 1889)	x				
*Platylomia brevis* Distant, 1912	x			x	
*Platylomia ficulnea* (Distant, 1892)	x		x		
*Platylomia insignis* Distant, 1912		x			
*Platylomia larus* (Walker, 1858)	x				x
*Platylomia malickyi* Beuk, 1998			x		
*Platylomia operculata* Distant, 1913			x		
*Platylomia radha* (Distant, 1881)	x	x	x	x	
*Platylomia vibrans* (Walker, 1850)	x				
*Zaphsa princeps* Lee & Emery, 2014	x				
** Fidicinini **	**1**				
*Proarna hilaris* (Germar, 1834)	x				
** Gaeanini **	**12**	**3**	**7**	**2**	**1**
*Ambragaeana stellata stellata* (Walker, 1858)	x				
*Ambragaeana stellata* var. *a* (Distant, 1892)	x				
*Balinta delinenda* (Distant, 1888)	x				
*Balinta octonotata octonotata* (Westwood, 1845)	x	x	x	x	
*Balinta octonotata* var. *a* Distant, 1892	x		x		
*Balinta octonotata* var. *b* Distant, 1892			x		
*Balinta sanguiniventris* Ollenbach, 1929	x				
*Balinta tenebricosa tenebricosa* (Distant, 1888)			x		
*Balinta tenebricosa* var. *a* Distant, 1892			x		
*Callogaeana annamensis* var. *b* (Distant, 1892)	x				
*Callogaeana festiva festiva* (Fabricius, 1803)	x	x			
*Gaeana atkinsoni* Distant, 1889	x				
*Gaeana consors* Atkinson, 1884	x		x		
*Gaeana maculata maculata* (Drury, 1773)	x		x		x
*Sulphogaeana sulphurea* (Westwood, 1839)	x	x		x	
** Lahugadini **	**1**				
*Lahugada dohertyi* (Distant, 1891)	x				
** Leptopsaltriini **	**3**	**1**	**1**	**1**	
*Manna tenuis* Lee & Emery, 2013	x				
*Mosaica irregularis* Lee & Emery, 2013	x				
*Neoterpnosia donghai* Lee & Emery, 2014	x				
*Neoterpnosia oberthuri* (Distant, 1912)		x			
*Neoterpnosia versicolor* (Distant, 1912)			x	x	
** Platypleurini **	**21**	**2**	**6**	**5**	**5**
*Oxypleura atkinsoni* (Distant, 1912)			x		
*Platypleura affinis affinis* (Fabricius, 1803)	x				
*Platypleura affinis distincta* Atkinson, 1884	x				
*Platypleura andamana* Distant, 1878	x				
*Platypleura assamensis* Atkinson, 1884	x	x			
*Platypleura badia* Distant, 1888			x		
*Platypleura basialba* (Walker, 1850)	x				
*Platypleura basiviridis* Walker, 1850	x				
*Platypleura bufo* (Walker, 1850)	x				
*Platypleura capitata* (Olivier, 1790)	x				x
*Platypleura cervina* Walker, 1850	x				
*Platypleura coelebs* Stål, 1863	x				
*Platypleura hampsoni* (Distant, 1887)	x				
*Platypleura inglisi* Ollenbach, 1929	x				
*Platypleura insignis* Distant, 1879			x		
*Platypleura intermedia* Liu, 1940					x
*Platypleura mackinnoni* Distant, 1904	x			x	
*Platypleura nobilis nobilis* (Germar, 1830)	x		x		
*Platypleura octoguttata octoguttata* (Fabricius, 1798)	x				x
*Platypleura polita polita* (Walker, 1850)	x				
*Platypleura polita* var. *a* (Distant, 1889)	x				
*Platypleura sphinx* Walker, 1850	x				
*Platypleura watsoni* (Distant, 1897)			x		
*Platypleura westwoodi* Stål, 1863					x
*Pycna himalayana* (Naruse, 1977)				x	
*Pycna minor* Liu, 1940t	x				
*Pycna montana* Hayashi, 1978				x	
*Pycna repanda repanda* (Linnaeus, 1758)	x	x	x	x	x
*Pycna verna* Hayashi, 1982	x			x	
** Polyneurini **	**3**		**1**	**2**	
*Angamiana aetherea* Distant, 1890	x				
*Formotosena montivaga* (Distant, 1889)	x				
*Polyneura ducalis* Westwood, 1840	x		x	x	
*Polyneura laevigata* Chou & Yao, 1986				x	
** Sonatini **	**5**	**1**	**1**	**3**	
*Distantalna splendida splendida* (Distant, 1878)	x		x		
*Hyalessa expansa* (Walker, 1858)	x			x	
*Hyalessa mahoni* (Distant, 1906)	x				
*Hyalessa melanoptera* (Distant, 1904)	x				
*Hyalessa obnubila* (Distant, 1888)	x	x		x	
*Hyalessa stratoria* (Distant, 1905)				x	
** Talaingini **	**2**		**3**		
*Talainga binghami* Distant, 1890			x		
*Talainga japrona* Ollenbach, 1929	x		x		
*Talainga naga* Ollenbach, 1929	x		x		
** Tosenini **	**4**	**1**	**2**	**4**	
*Tosena albata* Distant, 1878	x		x	x	
*Tosena dives* (Westwood, 1842)	x	x		x	
*Tosena mearesiana* (Westwood, 1842)	x			x	
*Tosena melanoptera* (White, 1846)	x		x	x	
** TIBICININAE **	**4**				
** Tibicinini **	**4**				
*Paharia lacteipennis* (Walker, 1850)	x				
*Subtibicina tigris* Lee, 2012	x				
*Tibicina casyapae* (Distant, 1888)	x				
*Tibicina reticulata* (Distant, 1888)	x				
**TOTAL**	**189**	**19**	**81**	**46**	**22**

**Table 3.** Regional comparison of species and generic diversity, ordered by the number of valid species recorded in each country.

**Country**	**Species**	**Genera**	**Citation**
India & Bangladesh	189	64	***present study***
Thailand	148	35	Sanborn et al. 2007
Vietnam	111	36	Lee 2008
Myanmar	81	34	***present study***
Laos	60	33	Lee 2014
Nepal	46	28	***present study***
Pakistan	30	13	Ahmed and Sanborn 2010, Ahmed et al. 2010
Cambodia	25	16	Lee 2010
Sri Lanka	22	12	***present study***
Bhutan	19	16	***present study***
